# Engineering a modular FAP-targeting ferritin-based drug nanocarrier for enhanced glioblastoma theranostics

**DOI:** 10.7150/thno.125403

**Published:** 2026-01-21

**Authors:** Yi-Hsiang Tseng, Jia-Yu Lin, Chia-Pao Chuang, Hsiao-Ching Su, Teh-Wei Wang, Kuo-Chen Wei, Feng-Ting Huang, Chiun-Wei Huang

**Affiliations:** 1 Department of Biochemical Science & Technology, National Taiwan University, Taipei, 106319 Taiwan.; 2 Project Division of Generative AI Utilization Aging Cells, The Institute of Medical Science, The University of Tokyo, Tokyo, Japan.; 3 Neuroscience Research Center and Department of Neurosurgery, Linkou Chang Gung Memorial Hospital, Taoyuan, 33305 Taiwan.; 4 School of Medicine, Chang Gung University, Taoyuan, 33302 Taiwan.; 5 Department of Medical Research and Development, Linkou Chang Gung Memorial Hospital, Taoyuan, 33305 Taiwan.

**Keywords:** glioblastoma, fibroblast activation protein, ferritin-based drug carrier, sortase-mediated ligation, PET imaging

## Abstract

**Rationale:** Glioblastoma multiforme (GBM) is an aggressive brain tumor marked by diffuse infiltration, a complex microenvironment, and poor drug delivery due to the blood-brain barrier. Fibroblast activation protein (FAP), widely expressed by cancer-associated fibroblasts (CAFs), emerges as a promising yet underexploited target for drug delivery.

**Methods:** Here, a modular ferritin-based drug carrier (FDC) functionalized with an optimized FAP-targeting ligand using site-specific sortase A-mediated ligation was developed. This approach ensures precise surface modification while preserving ferritin's structure and drug-loading capacity. Monomethyl auristatin E (MMAE), a potent cytotoxin, is stably encapsulated to create a dual-targeting nanocarrier aimed at FAP and transferrin receptor 1.

**Results:** In orthotopic glioma mouse models, the resulting FDC enables pH-responsive MMAE release, enhances tumor targeting and cellular uptake, reduces tumor burden, prolongs survival, and minimizes systemic toxicity compared to free MMAE. Furthermore, spatial transcriptomic analyses and immunohistochemistry data reveal that this therapeutic approach reshapes the tumor microenvironment by enhancing the spatial proximity between CAFs and cytotoxic immune cells and activating multiple immune pathways.

**Conclusions:** This study presents a precision-engineered nanoplatform for FAP-targeted GBM therapy, provides novel insights into the stromal-immune dynamics of GBM under therapeutic pressure and supports the rationale for combining CAF modulation with immunotherapy to achieve durable tumor control.

## Introduction

Glioblastoma (GBM) stands as the most prevalent and aggressive type of brain tumor, categorized as a grade IV tumor within the World Health Organization (WHO) classification system 1-4. Malignant gliomas represent the foremost cause of brain tumor-related fatalities across all age groups, including both children and adults 5-7. Despite considerable advancements in combination chemoradiotherapies, GBM remains largely incurable due to its pronounced capacity for cell invasion and infiltrative nature, resulting in incomplete surgical resections 8-10. This leads to a recurring problem and subsequent development of drug and radiation resistance during adjuvant treatments 11-13. Consequently, the life expectancy for GBM patients typically hovers around 10-12 months, with a disheartening 5-year survival rate of only 5.8 % 4, 14-17. As such, an urgent need exists for innovative strategies in GBM treatment 18.

Gliomas are a diverse group of clinically and molecularly distinct tumors, each containing varied malignant cell populations that share transcriptional features with other tumor types 19-21. Fibroblast activation protein (FAP), a membrane-bound serine protease, is a key target for diagnostic and therapeutic research 22. FAP is generally expressed at very low or undetectable levels in most normal adult tissues. Its expression becomes transiently elevated only under specific remodeling or inflammatory conditions, such as wound healing, myocardial infarction-associated cardiac repair, or chronic inflammation (e.g., rheumatoid arthritis synovium) 23-26. In contrast, glioblastomas and other high-grade gliomas exhibit pronounced FAP expression within the tumor stroma and perivascular niches, supporting its use as a highly selective target for tumor-directed drug delivery 23, 27, 28. Clinical studies show that radiolabeled FAPIs, such as ⁶⁸Ga-FAPI-04 and ⁶⁸Ga-FAPI-46, provide high tumor-to-background contrast in variety of solid tumors 29-33. Although FAP-targeting tracers hold potential as tumor imaging agents, their lack of therapeutic function may restrict their clinical impact 34, 35. Labeling these tracers with therapeutic radioisotopes for targeted radioligand therapy (RLT) is a possible strategy, but RLT's effectiveness is often limited, due to the insufficient tumor penetration and tumor heterogeneity 35-38. Additionally, the radioisotope's half-life should align with the targeting ligand's biological half-life, which is rarely the case. FAP-targeting tracers, being small molecules, exhibit rapid clearance in vivo, further complicating their use.

Conventional cancer treatments often involve systemic chemotherapy, which can cause severe side effects and toxicity by harming healthy tissues. Developing efficient drug delivery systems is critical to enhance therapeutic efficacy while minimizing adverse effects 39-43. In this study, we initially attempted to link FAP-targeting peptides with chemotherapeutic drugs like doxorubicin (DOX) and monomethyl auristatin E (MMAE) to create pro-drugs. However, the drugs' intrinsic properties (e.g., hydrophilicity, hydrophobicity, bulky structures and linker cleavage efficiency) significantly altered the pharmacokinetic and pharmacodynamic (PK/PD) profiles of the pro-drugs compared to the small-molecule FAP-targeting peptides, reducing therapeutic efficacy.

Conversely, human ferritin heavy chain 1 (HFn), a ferritin subunit, is essential for safely storing iron in cells 44-46 and has recently emerged as a promising nanocarrier due to its biocompatibility, ease of purification, and consistent production quality, making it suitable for clinical nanomedicine applications 47-49. Comprising 24 subunits, HFn forms a compact, hollow sphere approximately 12 nm in diameter, ideal for nanocarrier use. Notably, HFn binds to transferrin receptor 1 (TfR1), which is overexpressed in many cancers, facilitating cellular uptake via endocytosis 50-53. Evidence also suggests HFn may interact with TfR1 or similar receptors on brain endothelial cells or neurons, potentially enabling blood-brain barrier (BBB) crossing through receptor-mediated mechanisms, highlighting its potential for brain cancer treatment and central nervous system (CNS) drug delivery 54. Leveraging these unique traits, this study seeks to precisely attach FAP-targeting peptidomimetics to the N-terminal of ferritin using an evolved sortase A (eSrtA)-based ligation approach, enhancing its potential as a nanocarrier in ferritin-based drug carriers (FDCs) 55.

To investigate the molecular mechanisms underlying the observed therapeutic effects, we performed spatial transcriptomic analysis using the 10x Genomics Visium HD platform. The spatial transcriptomic analysis provides compelling evidence that therapy initiates profound structural and immunological remodeling within the GBM tumor microenvironment. Key features of this remodeling include the contraction of the tumor core, expansion of immune-active peripheries, widespread induction of interferon-stimulated genes, and deep infiltration of cytotoxic T lymphocytes. Gaining deeper insight into the fibroblast landscape, including their status and distribution across various pathological stages, will yield valuable temporal and spatial data. This knowledge could help to refine strategies for improving long-term prognostic outcomes in glioma therapy and beyond.

## Materials and Methods

### Materials

**Figure 1:**
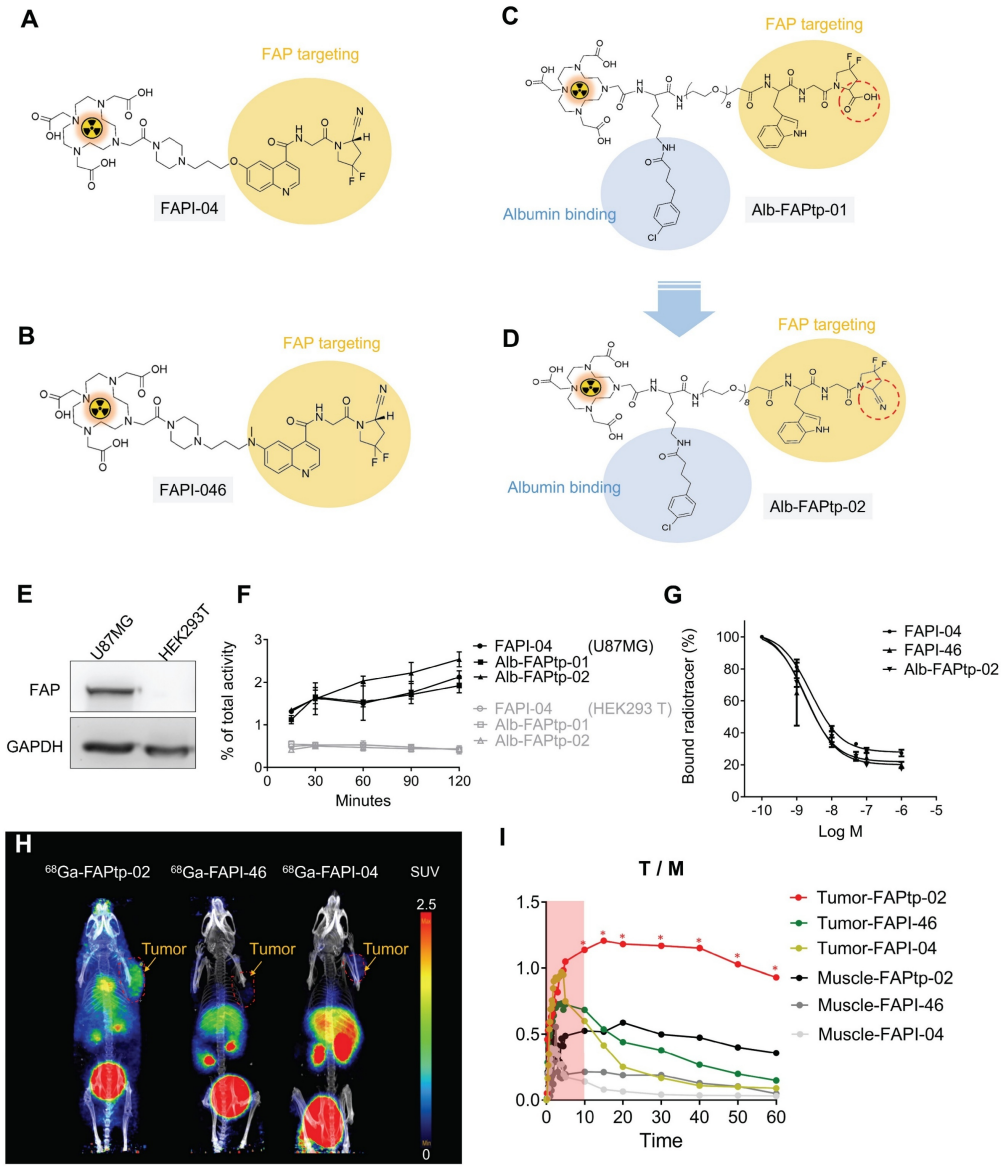
Representative chemical structures and functional groups of (A) FAPI-04, (B) FAPI-46, and (C) Alb-FAPtp-01 and (D) the developed peptide tracer (Alb-FAPtp-02). (E) FAP expression level in U-87MG and HEK293T. (F) Cellular uptake of ⁶⁸Ga-FAPI-04, ⁶⁸Ga-FAPI-46, and ⁶⁸Ga-Alb-FAPtp-02 in U-87 MG and HEK293T cells. No significant. (G) Competitive binding assay showing inhibition of tracer uptake in U-87 MG cells by non-radiolabeled FAPI-04, confirming FAP-specific binding (n = 3, mean ± s.d.).** (**H**)** *In vivo* dynamic PET imaging analysis comparison of FAP tracers. Representative dynamic PET/CT imaging of ⁶⁸Ga-Alb-FAPtp-02, ⁶⁸Ga-FAPI-46 and ⁶⁸Ga-FAPI-04 in mice with glioma xenografts. (I) the corresponding time-activity curves of radiotracers for U-87 MG tumors and muscle. Statistical significance was determined using one-way ANOVA followed by Tukey's HSD post hoc test. *p < 0.05.

All chemicals used in this study were of analytical grade and were obtained from commercial suppliers, including Aldrich (St. Louis, MO, USA), and were used as received without additional purification. The FAPI-04 precursor was sourced from KriSan Biotech Co. (Tainan, Taiwan), a PICS/GMP-compliant pharmaceutical manufacturer, whereas the FAPI-46 precursor was purchased from MedChemExpress (MCE, New Jersey, USA). The FAP-targeting peptide DOTA-Alb-FAPtp-02 (Figure 1C), chemically defined as DOTA-Lys (4-p-chlorophenyl) butyric acid)-PEG8-Trp-Gly-4,4-difluoro-2-cyanopyrrolidine) , was designed in-house and synthesized by Mission Biotech (Taipei, Taiwan). The ^68^GaCl_3_ solution was obtained by eluting a ^68^Ge/^68^Ga generator (ITM Medical Isotopes, Munich, Germany) with 0.05 N HCl. Radiochemical purity was assessed using an analytical reverse-phase HPLC system equipped with a dual-wavelength UV detector (Waters 2487, Waters, Milford, MA, USA) and a Synergi Hydro-RP column (4 μm, 80Å, 150 × 4.6 mm; Phenomenex, Torrance, CA, USA). Chromatographic separation was performed at a flow rate of 1 mL/min using a gradient elution program consisting of solvent A (0.1% trifluoroacetic acid in water) and solvent B (0.1% trifluoroacetic acid in acetonitrile). The gradient was held at 98% A / 2% B for the first 2 min, followed by a linear increase to 65% B over 30 min. The FAP ligation peptides Trp-Gly-(4,4-difluoro-Pro)-Ala-Trp-Gly-(4,4-difluoro-Pro)-Ala-Arg-Gly-Asp-Gly-Glu-Ala-PEG_12_-Leu-Pro-Glu-Thr-Gly-Gly (FAPtp) were designed by us and purchased from APOLO Biochemical, Inc (Hsinchu, Taiwan).

### Radiochemistry

The ^68^Ga labeling procedure was performed as previously described, yielding radiochemical purity > 95% 56. Briefly, radiolabeling with ^68^Ga was carried out following established protocols, consistently achieving radiochemical purity greater than 95%. In brief, generator-eluted ^68^GaCl₃ (0.05 M HCl) was combined with 250 µL of 0.1 M ammonium acetate buffer to produce ^68^Ga-acetate (activity range 555-370 MBq/mL). The mixture pH was adjusted to 4.0-4.5 and incubated with a DOTA-conjugated peptide solution (25-35 µg in 20 µL of 0.1 M ammonium acetate buffer). The reaction vessel was sealed and heated at 95 °C for 5-10 min to synthesize the ^68^Ga-DOTA-peptide complex. The total preparation time was approximately 20-25 min. Radiochemical purity, assessed via radio-TLC, exceeded 95%, with a molar activity of 18.51 ± 1.35 MBq/nmol. HPLC analysis confirmed that the radiochemical purity was consistently above 95%. The ^68^Ga labeling quality results are listed in Table S2.

### Cellular Culture

U-87 MG (human glioblastoma) and HEK-293T (human embryonic kidney) cells were obtained from the Bioresource Collection and Research Center (BCRC, Hsinchu, Taiwan). Cells were cultured in minimum essential medium (MEM) supplemented with 10% fetal bovine serum (FBS), 2 mM L-glutamine, 0.1 mM nonessential amino acids, 1 mM sodium pyruvate, and 100 U/mL penicillin-streptomycin. Murine glioblastoma GL261 cells, a generous gift from Dr. Kuo-Chen Wei (Chang Gung Memorial Hospital, Linkou, Taiwan), were cultured in DMEM containing 10% FBS and 100 U/mL penicillin-streptomycin. All cells were cultured in a humidified incubator with 5% CO_2_ at 37 °C. All media and reagents were purchased from Thermo Fisher Scientific (Waltham, MA, USA).

### Cell-based Uptake and Competition Assays

U-87 MG and HEK-293T cells were plated in 24-well plates and cultured until they reached approximately 80-90% confluency. Cellular uptake was assessed by incubating cells with radiolabeled tracers (18.5 kBq) in 0.5 mL of Hank's balanced salt solution (HBSS) for 15, 30, 60, 90, and 120 min. For competitive binding assays, U-87 MG cells (2 × 10⁵ cells per well) were incubated with approximately 18.5 kBq of the ^68^Ga-labeled tracer in the presence of increasing concentrations of unlabeled FAPI-04 at 37 °C for 1 h. Following incubation, cells were washed twice with 1 mL of ice-cold phosphate-buffered saline (PBS) and subsequently lysed using 0.2 M NaOH containing 1% SDS. Cell-associated radioactivity was measured using a gamma counter (Cobra II, Packard Instruments, Downers Grove, IL, USA) and normalized to the total applied radioactivity, expressed as a percentage of counts per minute (CPM). All experimental conditions were performed in triplicate and independently repeated three times. Binding affinities were determined by nonlinear regression analysis of the competition curves using GraphPad Prism 6 software.

### pET28b-pre-G-HFn Plasmid Construction

To engineer the ferritin plasmid with polyglycine at the N-terminus, the polyglycine-encoding sequence was inserted into the N-terminus of the ferritin using the Q5® Site-Directed Mutagenesis Kit (NEB, E0554S), following the manufacturer's instructions. The resulting plasmid, pET28b-pre-G-HFn plasmid, was verified by Sanger sequencing.

### Recombinant Protein Expression and Purification

The pET28b-pre-G-HFn plasmid was transformed into *E. coli BL21(DE3)* cells and the bacteria were grown in LB medium at 37 °C until the optical density at 600 nm (OD₆₀₀) reached 0.7-0.8. Protein expression was induced with 0.2 mM isopropyl β-D-1-thiogalactopyranoside (IPTG) at 37 °C for 4 h. The bacterial cells were harvested and lysed by sonication. After centrifugation, the supernatant was heat-treated at 80 °C for 10 min to denature and remove unwanted proteins. The pre-G-HFn was then precipitated with 60% ammonium sulfate saturation, dissolved in PBS buffer, and dialyzed to remove residual salts. To remove the N-terminal methionine, pre-G-HFn, which contains the TEV cleavage site (ENLYFQG) behind methionine, was incubated with TEV protease at a 25:1 substrate-to-enzyme ratio. The processed protein was further purified by size-exclusion chromatography using a Sephacryl S-300 HR (GE Healthcare, 17-0599-01). Finally, G-HFn was concentrated using a 100 kDa centrifugal filter and stored in 10% glycerol at -80 °C. G-HFn with an eSortase A N-terminal tag was buffer-exchanged into PBS containing 10 mM CaCl₂ (pH 7.5). Subsequently, 1 mM peptides were mixed with 100 μM G-HFn subunits and incubated with 1 μM eSortase A overnight at 37 °C. After incubation, conjugated particles were separated from free peptides using a 100 kDa centrifugal filter. Conjugation efficiency was analyzed using the ImageJ software to quantify the pixel intensity of the G-HFn conjugated and unconjugated G-HFn bands. The percentage of conjugated G-HFn was calculated as follows: Conjugation (%) = [Conjugated Band Intensity / (Conjugated + Unconjugated Band Intensity)] × 100%.

### Characterization of FAPtp-HFn

The morphology of FAPtp-HFn was examined using a Hitachi H-7650 transmission electron microscope (TEM) at an acceleration voltage of 200 kV. For analysis, the protein samples (0.1-0.5 mg/mL) were applied to carbon-coated 200-mesh grids, stained with 2% phosphotungstic acid (PTA), washed with ddH_2_O, and air dried. Hydrodynamic diameter and size distribution in the solution were analyzed by a ZetaSizer Nano Z-S (Malvern Instruments).

### Preparation of MMAE@G-HFn

To encapsulate MMAE into the G-HFn nanocage, 1 μM G-HFn was disassembled by adjusting the solution to pH 2.0 using 0.5 M HCl. MMAE was then added to the protein solution at a final concentration of 500 μM. After a 5-min incubation, the G-HFn nanocages were reassembled by raising the pH to 7.4 using 0.5 M NaOH. The resulting solution was dialyzed for 16 h to remove unencapsulated MMAE molecules. Finally, MMAE@G-HFn was freeze-dried to concentrate the protein for subsequent ligation.

### Quantification of MMAE in MMAE@FAPtp-HFn

MMAE content in MMAE@FAPtp-HFn was quantified using high-performance liquid chromatography (HPLC) with a Waters 600 pump, 717 autosampler, and diode array detector (DAD). Chromatographic separation was performed on a C18 reversed-phase column (YMC-Pack ODS-AM, 250 × 4.6 mm, S-5 μm, 12 nm), and MMAE was detected using UV absorbance at 220 nm. The area under the peak (AU × min) of the injected sample was recorded and compared to a standard calibration curve of MMAE to determine MMAE concentration.

### MMAE Release Assay

MMAE@FAPtp-HFn nanoparticles were incubated in PBS at either pH 7.4 or pH 5.0 at 37 °C. At designated time points, 150 μL of release medium was collected and concentrated using a 100 kDa centrifugal filter. The resulting flow-through was collected and analyzed by HPLC. The amount of MMAE released at each time point was quantified using a standard calibration curve generated from free MMAE.

### Labeling of G-HFn and FAPtp-HFn

Fluorescein isothiocyanate (FITC) was dissolved in DMSO (1 mg/mL) and added to G-HFn and FAPtp-HFn in 100 mM carbonate buffer (pH 9.0). The mixture was incubated overnight at 4 °C, followed by purification using a PD MiniTrap G25 column (Cytiva, Uppsala, Sweden) to remove unreacted FITC. The FITC concentration was determined by measuring 484 nm excitation and 535 nm emission. Sulfo-Cyanine7 (Cy7) was dissolved in DMSO (10 mg/mL) and added to G-HFn and FAPtp-HFn. The mixture was incubated for 2 h at room temperature and purified similarly. Cy7 concentration was measured at 756 nm excitation and 779 nm emission. The concentration of the HFn protein was quantified using the Bradford assay.

*Cellular uptake and distribution***:** The cellular uptake and distribution of FAPtp-HFn were analyzed using a fluorescence microscopy. GL261 cells were incubated with FITC-labeled G-HFn or FAPtp-HFn nanoparticles at an equivalent FITC concentration (1 μM) at 37 °C for 5 min. After incubation, cells were washed twice with HBSS buffer to remove unbound nanoparticles. Finally, the nuclei were stained with Hoechst and visualized under a fluorescence microscope.

### *In Vitro* Cytotoxicity of MMAE@FAPtp-HFn

GL261 and U-87 MG cells were seeded in 96-well plates at a density of 5,000 cells per well in 100 μL of culture medium and allowed to adhere overnight. Subsequently, cells were treated with various concentrations of MMAE@FAPtp-HFn or free MMAE for 48 h. Following the treatment period, the culture medium was refreshed and supplemented with CCK-8 reagent. After a 1-hour incubation, cell viability was determined by recording the absorbance at 450 nm using a microplate reader.

### Xenograft Tumor Models

Animal studies were conducted according to guidelines approved by the IACUC of National Taiwan University (No. NTU-111-EL-00157) and the Laboratory Animal Center, Chang Gung Memorial Hospital (No. 2023121309). Male C57BL/6 mice, aged 6-8 weeks, were obtained the National Center for Biomodels in Taiwan. Housing conditions included a 12 h light/dark periodicity, with water and food available *ad libitum* throughout the study period. Subcutaneous tumor xenografts were generated by injecting 1 × 10^6^ GL261 cells into the right flank. Mice were subjected to *in vivo* imaging experiments when tumors reached a volume of approximately 800-900 mm³ (calculated as 0.5 × length × width^2^). In contrast, for longitudinal tumor growth monitoring, PET/CT acquisition was initiated at a tumor volume of 200 mm³. For GL261 glioma models, 1.5 × 10^4^ cells in 2 μL were stereotactically injected into the brains of C57BL/6 mice under isoflurane anesthesia (1-2% in oxygen) at a depth of 3.5 mm, 0.5 mm posterior and 2 mm lateral to the bregma. For U-87 MG models, 1 × 10^5^ cells in 2 μL were implanted at the same coordinates in nude mice under identical conditions. Tumor progression was monitored weekly by T_2_-weighted MRI.

### Small-animal PET/CT Imaging of Tumor

Small-animal PET/CT scans were performed using a nanoPET/CT system (Mediso Medical Imaging Systems). To evaluate tumor-targeting efficiency, mice received a rapid bolus injection of radiotracers (^68^Ga-FAPI-04, ^68^Ga-FAPI-46, or ^68^Ga-Alb-FAPtp-02) through the tail vein (100-150 μL in PBS, ~8-10 MBq). Dynamic PET acquisitions were recorded for 1 h following tracer administration. Animals were anesthetized with 2.0% isoflurane (Benson Medical Industries, Markham, ON, Canada) throughout the procedure. To ensure data accuracy, scatter, random, and attenuation corrections were applied to all PET images. Corresponding CT data, acquired with a transaxial field of 5.0 cm and an axial field of 8.5 cm, were collected using the same scanner. Image fusion and analysis were then conducted through PMOD version 4.0 (PMOD Technologies Ltd.). Regions of interest (ROIs) for the tumors were delineated by applying a threshold at 50% of the range between the peak and baseline activity within the integrated CT coronal sections. Muscle ROIs were drawn over the thigh region. Quantitative analysis of tumors and organs was performed using the mean standardized uptake value (SUVmean). For visualization, maximum intensity projection (MIP) images were reconstructed based on the highest voxel values. Tumor-to-background ratios were calculated by comparing tracer uptake in tumors with that in normal organs including muscle, heart, lung, liver, kidney, and brain.

### *In Vivo* IVIS Tumor-Targeting Imaging

Cy7-labeled FAPtp-HFn and Cy7-G-HFn were administered via tail vein injection, and imaged at 30 min, 1 h, 2 h, 4 h, and 24 h using IVIS system. The filter sets used for imaging had excitation and emission wavelengths of 710 nm and 760 nm, respectively. For *ex vivo* analysis, the mice were sacrificed 24 h post-injection, and various organs, including the tumor, brain, heart, lungs, muscles, liver, pancreas, bones, kidneys, and spleen, were dissected. The Cy7 fluorescence signals from the tumors and organs were acquired and quantified using the IVIS imaging system.

### *In Vivo* Evaluation of Therapeutic Efficacy of MMAE@FAPtp-HFn

For orthotopic glioma models, GL261 tumor cells were stereotactically implanted into the brains of mice. Animals were randomly divided into three groups: control, MMAE, and MMAE@FAPtp-HFn. Each group received intravenous tail vein injections of the respective treatment at a dosage of 0.15 mg/kg. Therapeutic efficacy was evaluated by survival analysis, MRI, and histological examination. Treatment endpoints were defined as severe tumor burden confirmed by MRI or >15% body weight loss. At sacrifice, tumors and major organs (heart, liver, kidney) were harvested, paraffin-embedded, and analyzed by hematoxylin and eosin (H&E) staining.

*MRI Analysis:* T_2_-weighted images were acquired using a RARE sequence (in-plane resolution = 70 µm, slice thickness = 0.5 mm, 12 coronal slices). Fat suppression was applied to enhance tumor contrast, and tumor volumes were quantified by manual ROI delineation using 3D Slicer 57.

### Brain Dissection, Embedding, and Sectioning

Control and MMAE@FAPtp-HFn-treated mice were euthanized following AVMA guidelines. Mice were initially anesthetized using 4-5% isoflurane and underwent transcardial perfusion with phosphate-buffered saline (PBS). Following the extraction of the brains, the tissues were stabilized in 4% paraformaldehyde (PFA). Subsequently, the specimens were processed for inclusion in formalin-fixed paraffin-embedded (FFPE) blocks for histological analysis. Coronal sections (5 µm thick) were mounted onto 10X Genomics Visium HD Spatial Gene Expression Slides (lot no: 173767), which contain millions of 2 × 2 µm spatially barcoded oligonucleotides within a 6.5 × 6.5 mm capture area. Slides were stored at -80 °C until further processing.

### Library Preparation and Sequencing

Spatial gene expression libraries were prepared using the Visium HD Reagent Kit and Visium Mouse Transcriptome Probe Kit v2 (10X Genomics), following the manufacturer's protocol for FFPE samples. Probe hybridization, ligation, and amplification were performed to capture transcriptomic data from high-resolution spatial features. Libraries were sequenced on an Illumina NovaSeq 6000 platform using 150 bp paired-end reads. Sequencing quality metrics indicated high data fidelity, with valid barcodes at 90.8%, valid UMIs at 99.9%, and Q30 scores exceeding 92% across barcode, probe, and UMI reads. Approximately 77.9% of spatial bins were located under tissue, and 99.0% of reads mapped to tissue-covered areas. Full sequencing quality metrics are provided in Table S5.

### Data Processing and Cluster Annotation

Raw sequencing data were processed using Space Ranger HD (v3.1.1) to align reads to the mm10 reference genome and generate spot-level gene expression matrices. Spatial coordinates and expression profiles were visualized using Loupe Browser HD (v8.1.2). Integrated analysis of control and treated samples was performed using reciprocal PCA (RPCA) and UMAP embedding via Seurat (v5.3.0) 58. Tumor regions were segmented into 12 clusters based on spatial location and gene expression signatures.

### CAF and Cytotoxic Effector Co-expression Mapping

Spots with enriched expression of cytotoxic effector-related genes (*Gzma, Gzmb, Gzmk, Prf1, Nkg7, Ifng*) were designated as cytotoxic effector spots. CAF-associated genes (*Acta2, Fap, Col1a1, Tnc, Pdgfra, Pdgfrb, Pdpn, S100a4*) were used to define CAF spots, which were subsequently mapped across tumor regions. Co-expression patterns of CAF and cytotoxic effector signatures were visualized using Loupe Browser (10x Genomics) to assess potential immune-stromal interactions.

### Differential Gene Expression (DEGs) and GO-Analysis

DEGs between treated and control samples were identified using log₂ fold change > 0.3 and FDR < 0.05. Multiple testing correction was performed using the Benjamini-Hochberg method. GO enrichment analysis was performed on overlapping upregulated DEGs from central, peripheral, and microglia-rich regions to identify immune-related biological processes.

*AUCell-Based Scoring of Immune Activity:* AUCell scoring was used to quantify immune-related pathway activity, including immunogenic cell death (mm). Sparse count matrices were extracted using GetAssayData, and ICD (immunogenic cell death) scores were computed using AUCell_buildRankings and AUCell_calcAUC (AUCell v1.16.0) 59. Scores were scaled and added to the metadata for downstream analysis. AUCell scores were extracted and grouped by sample condition. Violin plots were generated to compare ICD scores (*Calr, Hmgb1, Hsp90aa1, Hsp90b1, Hspa1a, Hspa1b, Ifnb1, Irf1, Irf7, Stat1, Cxcl9, Cxcl10, Ccl5, Il1b, Eif2ak3, Atf6, Ddit3, Xbp1, Tap1, B2m, Psmb8, Psmb9*) between control and treated groups. Statistical significance was assessed using the Wilcoxon rank-sum test. UMAP projections of AUCell scores were used to visualize spatial enrichment of ICD activity.

### Immunohistochemistry (IHC) and Quantitative Image Analysis

Immunohistochemical staining was performed on formalin-fixed, paraffin-embedded (FFPE) tissue sections cut at a thickness of 10 μm using the automated Leica BOND-MAX system (Leica Biosystems). Following deparaffinization, heat-induced epitope retrieval was performed for 20 min. Bond Epitope Retrieval Solution 1 (pH 6.0) was used for the following primary antibodies: Granzyme B (ab255598, 1:1600; Abcam), and CXCL10 (10937-1-AP, 1:50; Proteintech). Bond Epitope Retrieval Solution 2 (pH 9.0) was used for CD8α (ab316778, 1:400; Abcam) and HMGB1 (ab79823, 1:4000; Abcam). Detection was carried out using the BOND Polymer Refine Detection System (DS9800, Leica Biosystems) with 3,3'-diaminobenzidine (DAB) visualization and hematoxylin counterstaining.

Whole-slide images were acquired and imported into QuPath software (version 0.6.0) for quantitative analysis. Regions of interest (ROIs) were defined within the tumor area, and quantification strategies were tailored to each marker based on specific staining patterns. For CD8α, cells were identified using the same algorithm, with data presented as the percentage of positive cells relative to the total number of detected cells. For CXCL10, DAB-positive staining was segmented from the background using a trained pixel classifier and expressed as a percentage of the total tissue area. For HMGB1 and Granzyme B, cell detection was performed followed by classification of staining intensity into low (1+), medium (2+), and high (3+). The H-score was calculated using the formula: [1 × (% cells 1+)] + [2 × (% cells 2+)] + [3 × (% cells 3+)], ranging from 0 to 300.

### Statistical Analysis

Quantitative data are expressed as the mean ± s.d. Statistical analyses were performed using GraphPad Prism 6. An unpaired Student's *t*-test was employed to assess the differences between the experimental and control cohorts. Statistical significance was defined as *P* < 0.05.

## Results

### Re-design and Evaluation of FAP-Targeting Tracers

Previously, we developed a novel ^68^Ga-labeled FAP-targeting peptide tracer, ⁶⁸Ga-Alb-FAPtp-01, which exhibited superior tumor accumulation, retention, and biodistribution compared to the established ⁶⁸Ga-FAPI-04 56. However, the 2-cyanopyrrolidine group is critical for selective binding in FAPI tracers. Building on this, we re-designed the next-generation ⁶⁸Ga-Alb-FAPtp-02 tracer, incorporating 2-cyanopyrrolidine, and evaluated its stability, cellular uptake, FAP targeting, tumor retention, and biodistribution with FAPIs (Figure 1A-D).

To assess the performance of FAP-targeted tracers, ^68^Ga^3+^ was incorporated into FAPI-04, FAPI-46, and a newly designed Alb-FAPtp-02 peptide bearing a DOTA chelator, using established radiolabeling protocols. The radiochemical purity of all tracers remained above 95% per radio-HPLC analysis, with a mean molar activity of 13.37 ± 1.32 MBq/nmol. As detailed in Table S1, log *P* value for ^68^Ga-FAPI-04 (-2.92), ^68^Ga-FAPI-46 (-2.74), and ^68^Ga-Alb-FAPtp-02 (-2.88) indicated high hydrophilicity. Given the high purity of the labeled compounds, they were sterilized via 0.22 µm syringe filtration and immediately applied in subsequent in vitro binding assays and *in vivo* imaging without additional purification. Western blotting demonstrated that U-87 MG glioma cells expressed markedly higher levels of FAP than HEK293T cells (Figure 1E). Cellular uptake experiments in U-87 MG cells, seeded and incubated overnight, revealed similar tracer accumulation across all candidates during the first 30 min (Figure 1F).^ 68^Ga-Alb-FAPtp-02 showed a modestly higher uptake (> 2%) between 30 and 120 min, though this difference was not statistically significant. In contrast, HEK293T cells displayed only minimal, non-specific uptake for all tracers. It should be noted that the assay did not distinguish between surface binding and internalization. Competitive binding studies further determined IC₅₀ values of 1.73 nM, 1.93 nM, and 2.56 nM for ^68^Ga-FAPI-04,^ 68^Ga-FAPI-46, and ^68^Ga-Alb-FAPtp-02, respectively (Figure 1G). Although the overall uptake in U-87 MG cells was relatively modest (~2% of the total applied radioactivity), this result is consistent with the use of cells expressing endogenous levels of FAP. Unlike the commonly used HT-1080-FAP overexpression models, U-87 MG cells offer a more physiologically relevant system, thereby lending further credibility to the tracer binding and competition assay results.

To assess tumor specificity and pharmacokinetics, the novel FAP-targeting PET tracer ⁶⁸Ga-Alb-FAPtp-02 was evaluated in heterotopic U-87 MG tumor-bearing mice, alongside clinically established tracers ⁶⁸Ga-FAPI-04 and ⁶⁸Ga-FAPI-46 (Figure 1H). One-hour dynamic PET imaging was conducted, and quantitative analysis using SUV value revealed that, unlike the rapid systemic clearance of ⁶⁸Ga-FAPI-04 and ⁶⁸Ga-FAPI-46, ⁶⁸Ga-Alb-FAPtp-02 exhibited slower blood clearance and prolonged tumor retention, leading to superior tumor-to-background contrast (Figure 1I) and corresponding activity covering areas of each tracer are 65.15% (⁶⁸Ga-Alb-FAPtp-02) vs. 23.46% (⁶⁸Ga-FAPI-46) vs. 16.85% (⁶⁸Ga-FAPI-04), respectively (Figure S1). These enhanced pharmacokinetic properties are attributed to its albumin-binding design, which increases in vivo stability by minimizing renal excretion and proteolytic degradation, while a hydrophilic PEG linker reduced nonspecific uptake in clearance organs such as the liver and kidneys. Biodistribution analysis (time-activity curves) of major organs confirmed efficient glomerular filtration and faster clearance than ⁶⁸Ga-FAPI-04, despite a significantly prolonged circulatory half-life (up to 4 h versus ~10 min), enabling productive FAP binding in a relevant biological orientation (Figure S2). Minimal off-target accumulation was noted, with only weak signals in bone and negligible uptake in muscle or brain, confirming high FAP specificity.

### *In vivo* Dose Optimization of ⁶⁸Ga-Alb-FAPtp-02 for Tumor-Targeted PET Imaging

**Figure 2:**
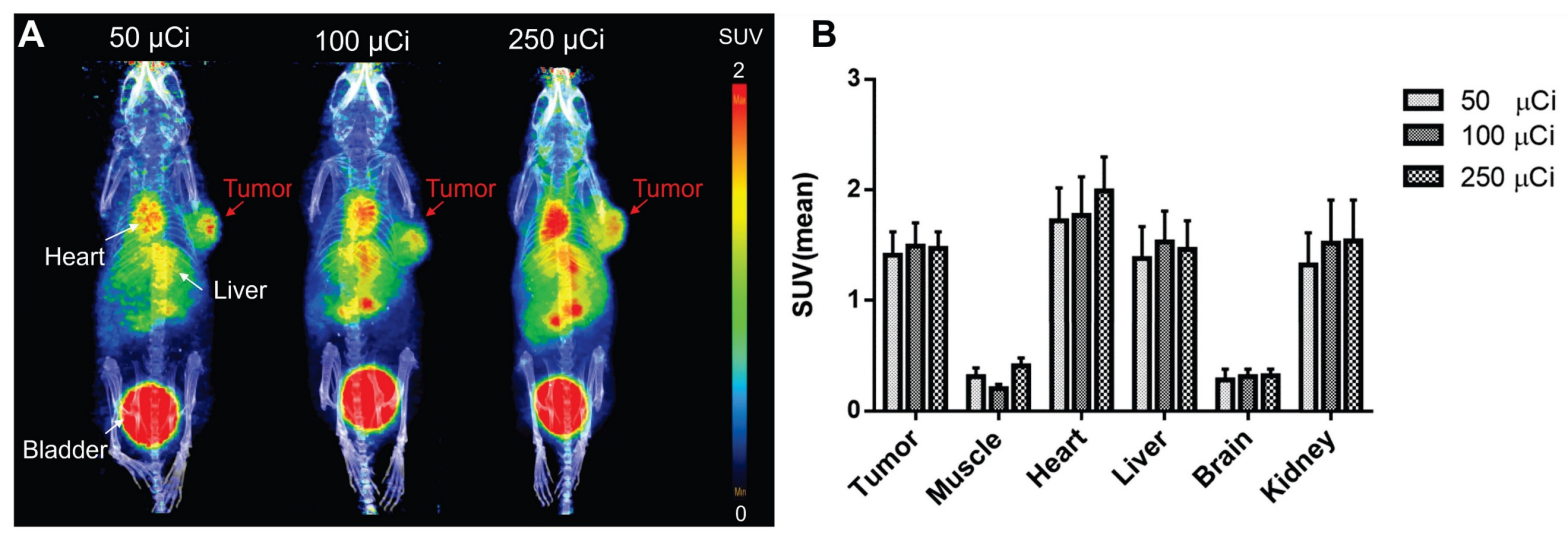
Representative PET/CT images and quantitative biodistribution of ^68^Ga-Alb-FAPtp-02 in tumor-bearing mice across three dose levels (50, 100, and 250 µCi). (A) *In vivo* PET/CT Visualization: Maximum intensity projection (MIP) PET/CT images of mice show the spatial distribution of the radiotracer at various injection activities. Significant tracer accumulation is observed in the tumor (located on the right flank), as well as the heart, liver, and bladder. (B) Quantitative Biodistribution: The bar graph displays the mean Standardized Uptake Value (SUVmean) across selected tissues, including the tumor, muscle, heart, liver, brain, and kidney. Data are presented as mean ± s.d. for all groups.

Dose optimization studies using 50, 100, and 250 μCi showed robust tumor uptake across all levels, with the 100 μCi dose demonstrating optimal pharmacokinetics by reducing nonspecific binding and background signal, especially in muscle, leading to tumor-to-background ratios of 6-7-fold (Figure 2). Uptake peaked at 30 min, slightly declined until 3 h, and then stabilized, indicating sustained tumor retention (Figure S3-4). Importantly, even at the lowest dose (50 μCi), tumor uptake (Figure 2) and imaging contrast were strong and significantly outperformed those of the most potent, clinically used FAPI-46 tracer (Figure 1I) (SUV mean 1.5 vs. 0.38 at 30 min p.i.). This highlights the tracer's efficiency and its potential to reduce radiation exposure. Its heterogeneous tumor distribution may serve as a phenotypic imaging biomarker, with longitudinal monitoring offering potential prognostic insights and reflecting dynamic changes in tumor FAP expression. These promising findings support the potential of ⁶⁸Ga-Alb-FAPtp-02 as a sensitive, predictive, and possibly theragnostic tool for guiding FAP-targeted imaging and treatment strategies.

### Site-Specific Conjugation of FAP-Targeting Peptides to Ferritin Nanocages via Sortase-Mediated Ligation

Encouraged by the favorable PET imaging results, we explored a FAP-activated peptide prodrug strategy (FAPtp-DOX)(Figure S5). Although PET imaging confirmed efficient tumor accumulation of FAPtp-DOX, its therapeutic benefit was limited, indicating inefficient drug release at the tumor site (Figure S3-8). To enhance potency, a FAPtp-MMAE construct was subsequently developed; however, its highly hydrophobic nature led to poor biodistribution and diminished tumor uptake (Figure S9). These observations underscore a key limitation of small-molecule prodrug designs—namely, that the physicochemical properties of the payload can substantially compromise delivery efficiency and therapeutic outcome. Therefore, we shifted to a FDCs platform, which offers superior biocompatibility, enhanced payload versatility, and pH-responsive drug release properties suitable for efficient intracellular delivery of potent cytotoxins such as MMAE.

To construct FAP-targeting FDCs, an initial strategy involved genetically fusing a FAP-targeting peptide (Trp-Gly-Pro-Ala-Trp-Gly-Pro-Ala) to the N-terminus of human ferritin (HFn) for expression in *E. coli*. However, the resulting fusion protein exhibited poor solubility, predominantly aggregating into the insoluble fraction (Figure S10A).

**Figure 3:**
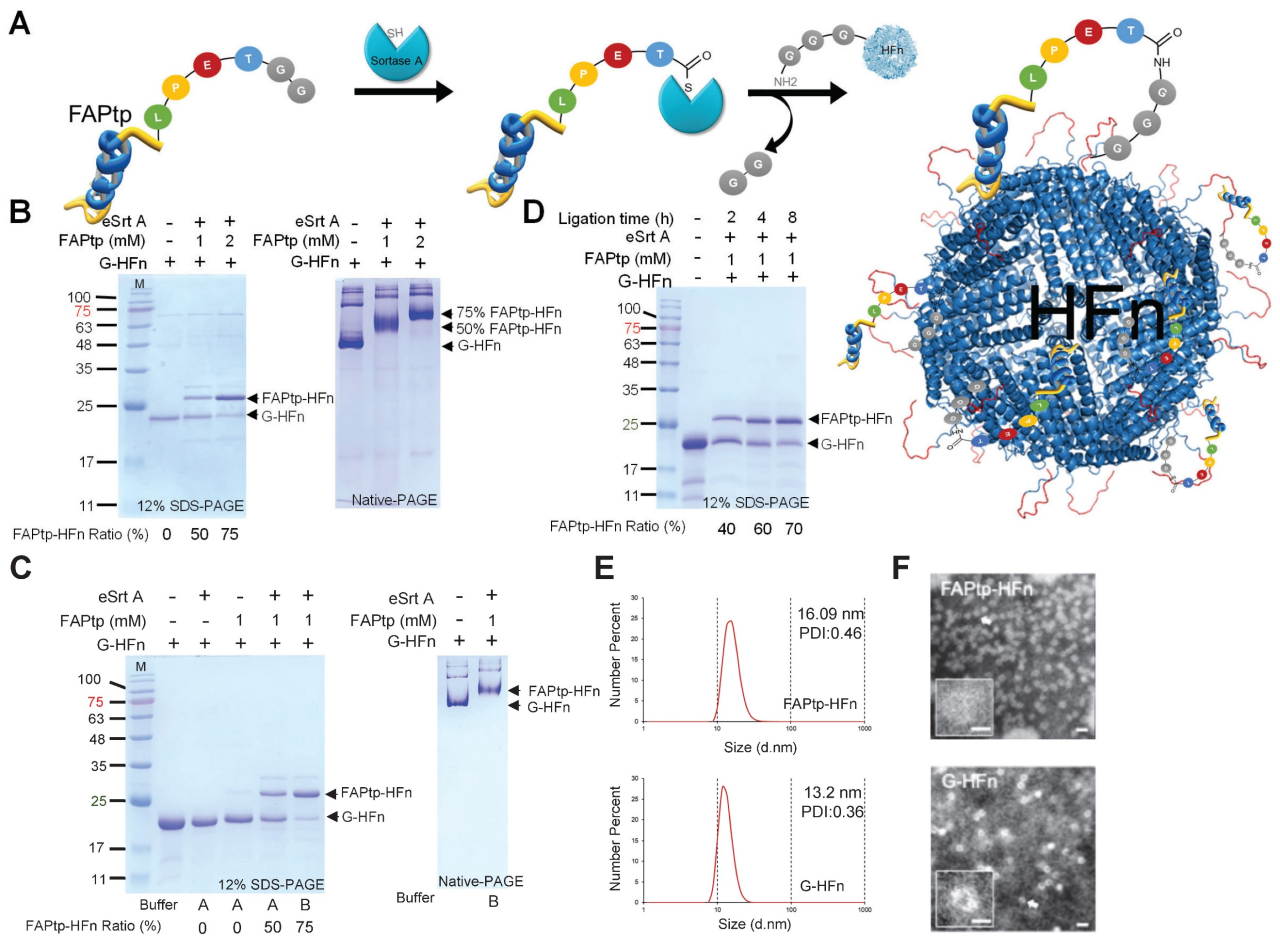
** Characterization of HFn following sortase-mediated ligation**. (A) Schematic representation of the sortase-mediated ligation process. (B) Representative SDS-PAGE and native-PAGE analyses of HFn ligation with FAPtp peptide at different peptide concentrations. FAPtp-HFn ratio: Percentage of FAPtp-HFn conjugation calculated as the intensity of the FAPtp-HFn band relative to the total intensity of conjugated and unconjugated G-HFn. (C) Effect of buffer composition on G-HFn ligation efficiency. Buffer A: PBS + Sortase A buffer. Buffer B: PBS + CaCl_2_. (D) Representative SDS-PAGE and native-PAGE analyses of HFn ligation with FAPtp peptide over a time course (2, 4, and 8 h). (E) Dynamic light scattering analysis of G-HFn and FAPtp-HFn. (F) TEM images of G-HFn and FAPtp-HFn (scale bar: 20 nm).

To overcome this limitation and enable the incorporation of synthetic functional groups—such as polyethylene glycol (PEG) linkers and difluorinated proline (diF-Pro)—a post-purification peptide conjugation strategy was implemented using sortase A (SrtA)-mediated ligation (Figure 3A). SrtA is a bacterial transpeptidase that catalyzes site-specific transpeptidation by recognizing the LPXTGG motif and ligating it to substrates bearing an N-terminal oligoglycine tag.

A recombinant evolved sortase A (eSrtA) was expressed and purified from *E. coli* (Figure S10B). To generate the sortase ligation acceptor, human ferritin (pre-G-HFn) was engineered with an N-terminal methionine followed by a TEV cleavage sequence and glycine. TEV treatment removed the methionine, yielding G-HFn with an exposed N-terminal glycine required for sortase-mediated ligation (Figure S10C). A fluorinated FAP-targeting peptide (FAPtp-pep) was conjugated to HFn nanocages at 37°C for 16 h. By adjusting the peptide concentration, the conjugation ratio could be precisely tuned, achieving ~50% and ~75% modification with 1 mM and 2 mM peptide, respectively (Figure 3B). Native-PAGE analysis confirmed uniform attachment of peptides to G-HFn nanocages, demonstrating effective and controllable modification. To further optimize the buffer conditions for sortase-mediated ligation, the standard sortase A reaction buffer was replaced with phosphate-buffered saline (PBS) supplemented with calcium chloride (CaCl₂), resulting in a notable increase in ligation efficiency. At a fixed FAPtp-pep concentration of 1 mM, the conjugation ratio increased from approximately 50% to 75%, indicating that the modified buffer conditions significantly enhanced the performance of sortase A-mediated ligation (Figure 3C). Conjugation could also be modulated by reaction time: at 1 mM FAPtp-pep, the modification ratio increased from 40%, 60%, to 70% after 2, 4, and 8 h, respectively (Figure 3D). Structural analyses indicated that the nanocages retained their integrity after modification. Dynamic light scattering (DLS) showed a modest increase in hydrodynamic diameter from 13.2 nm for G-HFn to 16.09 nm for FAPtp-HFn. (Figure 3E), while TEM confirmed the preservation of the characteristic spherical morphology (Figure 3F). Furthermore, storage stability assays confirmed that the FAPtp-functionalized nanocages maintained consistent physicochemical properties without significant aggregation for up to three weeks at -20°C (Table S3). Collectively, these results demonstrate that the optimized sortase-mediated ligation provides efficient, site-specific conjugation of fluorinated FAP-targeting peptides to ferritin nanocages with controllable modification levels and maintained structural integrity. This modular approach supports the development of FAPtp-HFn as a versatile and effective platform for FAP-targeted drug delivery.

### Dual-Targeting Capability and BBB Penetration of FAPtp-HFn Nanocarriers

**Figure 4:**
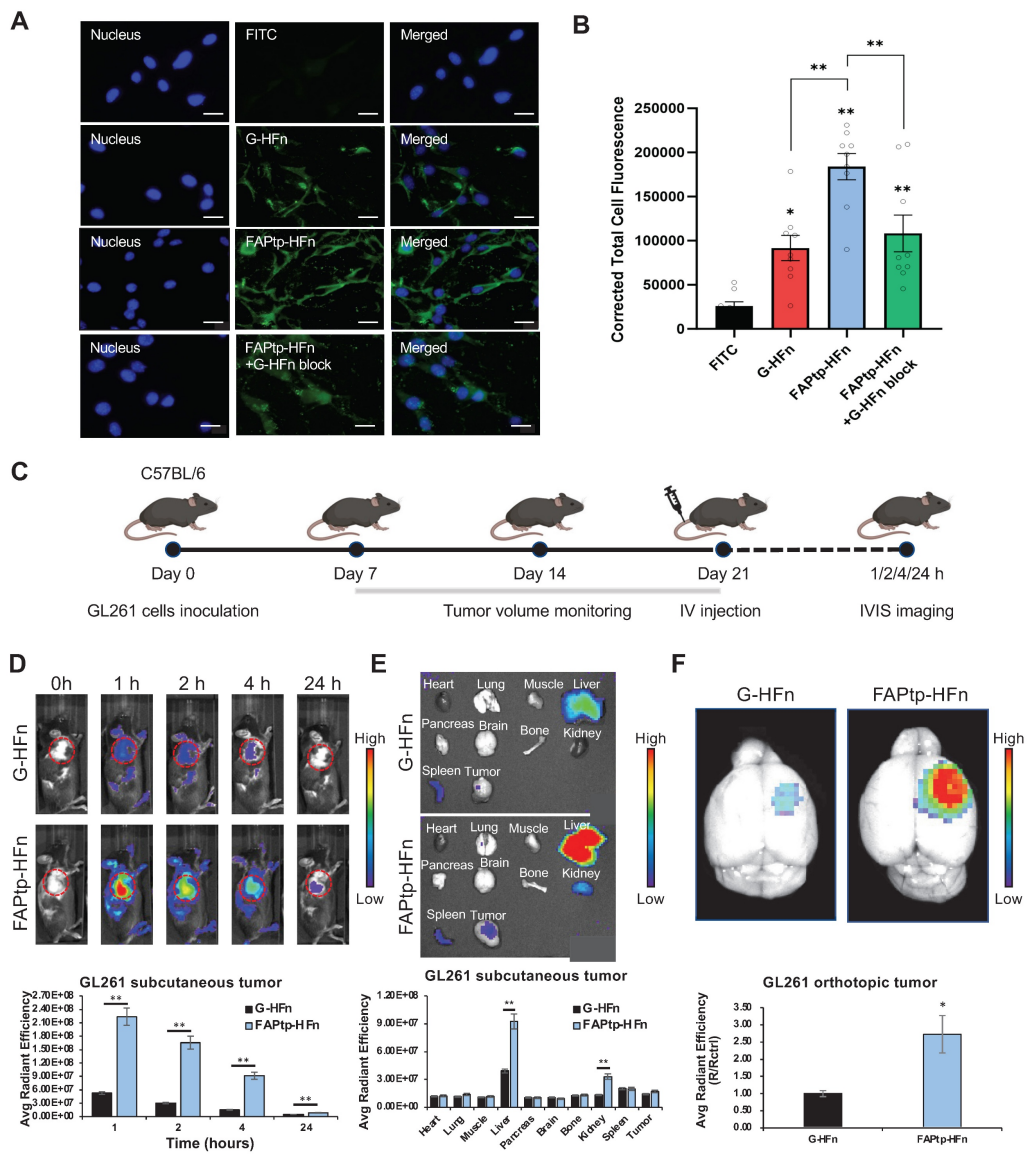
** Evaluation of the targeting efficiency of modified HFn in tumor cells.** (A) Fluorescence microscopy images showing the binding of FITC, G-HFn, FAPtp-HFn, and FAPtp-HFn with G-HFn as a TfR1 blocking agent (n = 9, three technical replicates per biological replicate). (Scale bar: 20 µm) (B) Corrected total cell fluorescence of FITC, G-HFn, FAPtp-HFn, and FAPtp-HFn with G-HFn as a blocking agent (n = 9). Data are presented as mean ± s.d. Statistical significance was determined by one-way ANOVA followed by Tukey's HSD post hoc test. *p < 0.05, **p < 0.01. *In vivo* tumor targeting and therapeutic potential of modified HFn. (C) Schematic representation of the imaging schedule for evaluating tumor-targeting efficiency. (D) *In vivo* tumor-targeting capability of FAPtp-HFn in a GL261 subcutaneous glioma mouse model (n = 3). (E) Biodistribution of FAPtp-HFn in GL261 tumor-bearing mice at 24 h post-intravenous injection (n = 3). (F) Fluorescence signals in orthotopic tumors of GL261-bearing mice. Comparative analysis of fluorescence intensity in orthotopic tumors treated with Cy7-labeled G-HFn (n = 3) or FAPtp-HFn (n = 3) at 24 h post-injection, normalized to cerebellar signal. Statistical significance was determined using one-way ANOVA followed by Tukey's HSD post hoc test. *p < 0.05, **p < 0.01.

The tumor-targeting performance of peptide-modified ferritin nanocarriers was first evaluated *in vitro*. FITC-labeled FAPtp-HFn nanoparticles were incubated with GL261 glioblastoma cells, which endogenously express both TfR1 and FAP (Figure S11). Fluorescence microscopy revealed rapid cellular uptake, with FITC-FAPtp-HFn exhibiting nearly two-fold stronger intracellular signals than FITC-labeled G-HFn within just 5 minutes (Figure 4A) and consistently outperformed free dye across all modification densities (Figure S12). Crucially, this rapid accumulation occurred despite the fact that N-terminal modification with a non-targeting control peptide inherently induces a transient lag in uptake kinetics (Figure S13). This suggests that the high affinity of the FAP-targeting peptide effectively overcomes the steric hindrance of conjugation. Pre-treatment with excess unlabeled G-HFn to block TfR1 binding markedly reduced the fluorescence of FITC-FAPtp-HFn to levels comparable to G-HFn (Figure 4B), confirming that the enhanced binding specificity arises from the synergistic dual-targeting effect of TfR1 recognition and FAP peptide modification.

The targeting efficacy and biodistribution of FAPtp-HFn were further assessed *in vivo*. In subcutaneous GL261 tumor-bearing mice, intravenous administration of Cy7-labeled FAPtp-HFn led to rapid and sustained tumor accumulation, with peak fluorescence at 1 h and detectable signals up to 24 h post-injection. Compared with Cy7-G-HFn, Cy7-FAPtp-HFn achieved ~4.4-, 5.7-, 6.3-, and 1.8-fold higher tumor fluorescence intensities at 1, 2, 4, and 24 h, respectively (Figure 4C and 4D). *Ex vivo* imaging revealed liver, spleen, and kidney as the main clearance organs for both nanocarriers (Figure 4E).

Importantly, in an orthotopic GL261 glioma model, Cy7-FAPtp-HFn demonstrated efficient BBB penetration and selective tumor accumulation, with fluorescence intensity ~2.7-fold higher than Cy7-G-HFn and ~4-fold higher than in contralateral brain tissue (Figure 4F, S14), consistent with TfR1-mediated transcytosis. Comparable tumor-targeting and biodistribution were also observed in human U-87 MG glioblastoma models using FAPtp-HFn nanocarriers (Figure S15), further supporting translational potential.

Collectively, these findings highlight the ability of FAPtp-HFn nanocarriers to achieve efficient tumor cell binding, selective BBB penetration, and dual-targeting through TfR1 and FAP, underscoring their promise for glioblastoma imaging and drug delivery.

### *In vitro* Cytotoxicity and pH-Responsive Drug Release of MMAE@FAPtp-HFn Nanocages

**Figure 5:**
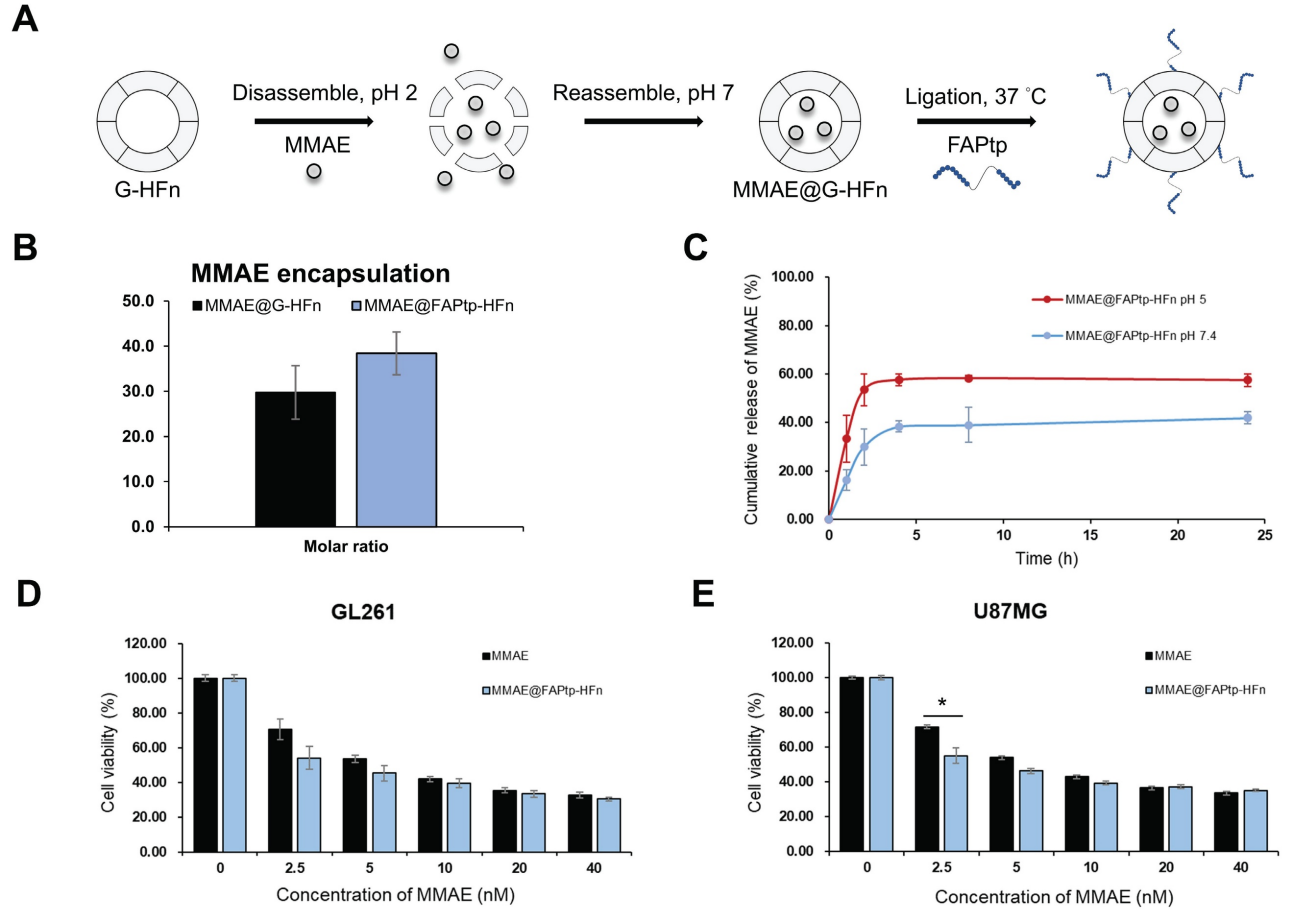
** Encapsulation and release of MMAE from modified HFn.** (A) Schematic representation of MMAE@FAPtp-HFn preparation. (B) Encapsulation of MMAE into FAPtp-HFn using a pH-mediated loading method (n = 3; *p* > 0.05). (C) Diagram illustrating the pH-responsive release of MMAE from FAPtp-HFn at pH 5 and pH 7. (D) Cytotoxicity of MMAE@FAPtp-HFn in GL261 cancer cells (n = 3). (E) Cytotoxicity of MMAE@FAPtp-HFn in U-87MG cancer cells (n = 3). Data are presented as mean ± s.d. Statistical significance was determined using one-way ANOVA followed by Tukey's HSD post hoc test. *p < 0.05, **p < 0.01.

To evaluate the potential of FAPtp-HFn nanocages as chemotherapeutic carriers, MMAE—a potent microtubule inhibitor commonly used in antibody-drug conjugates—was encapsulated into the nanocages using a pH-mediated loading strategy prior to peptide conjugation (Figure 5A). A standard calibration curve was established using high-performance liquid chromatography (HPLC) to quantify the drug loading efficiency (Figure S16). Post-encapsulation and peptide ligation, biophysical assessments confirmed that the resulting MMAE@FAPtp-HFn nanocages retained their native morphology and structural integrity (Figure S17). On average, each nanocage successfully encapsulated approximately 38.5 ± 5 MMAE molecules, with an encapsulation efficiency comparable to that of unmodified G-HFn (Figure 5B).

The release kinetics of MMAE@FAPtp-HFn nanocages were evaluated at pH 7.4 and pH 5.0 to mimic the conditions of systemic circulation and the lysosomal compartment, respectively. This setup allowed for a comparative characterization of the drug release profile in physiological versus acidic environments. The results demonstrated a slow and sustained release of MMAE at neutral pH over 24 h, indicating stable drug retention. In contrast, under acidic conditions, a markedly faster and continuous release was observed, consistent with a pH-responsive release behavior (Figure 5C).

We further examined therapeutic effectiveness by performing CCK-8 assays on two glioblastoma cell lines: the murine GL261 and the human U-87 MG. The cytotoxicity of MMAE@ FAPtp-HFn was comparable to that of free MMAE, confirming that encapsulation did not compromise the drug's bioactivity (Figure 5D-E). This effect remained robust across all peptide modification densities, showing no significant variation (Figure S18). Nano live-cell tomographic microscopy further confirmed that MMAE@FAPtp (40 nM) induced morphological alterations and promoted cell death in a time-dependent manner (Figure S19).

### MMAE@FAPtp-HFn Nanocarriers Enhance Therapeutic Efficacy in Orthotopic Glioblastoma Models

**Figure 6:**
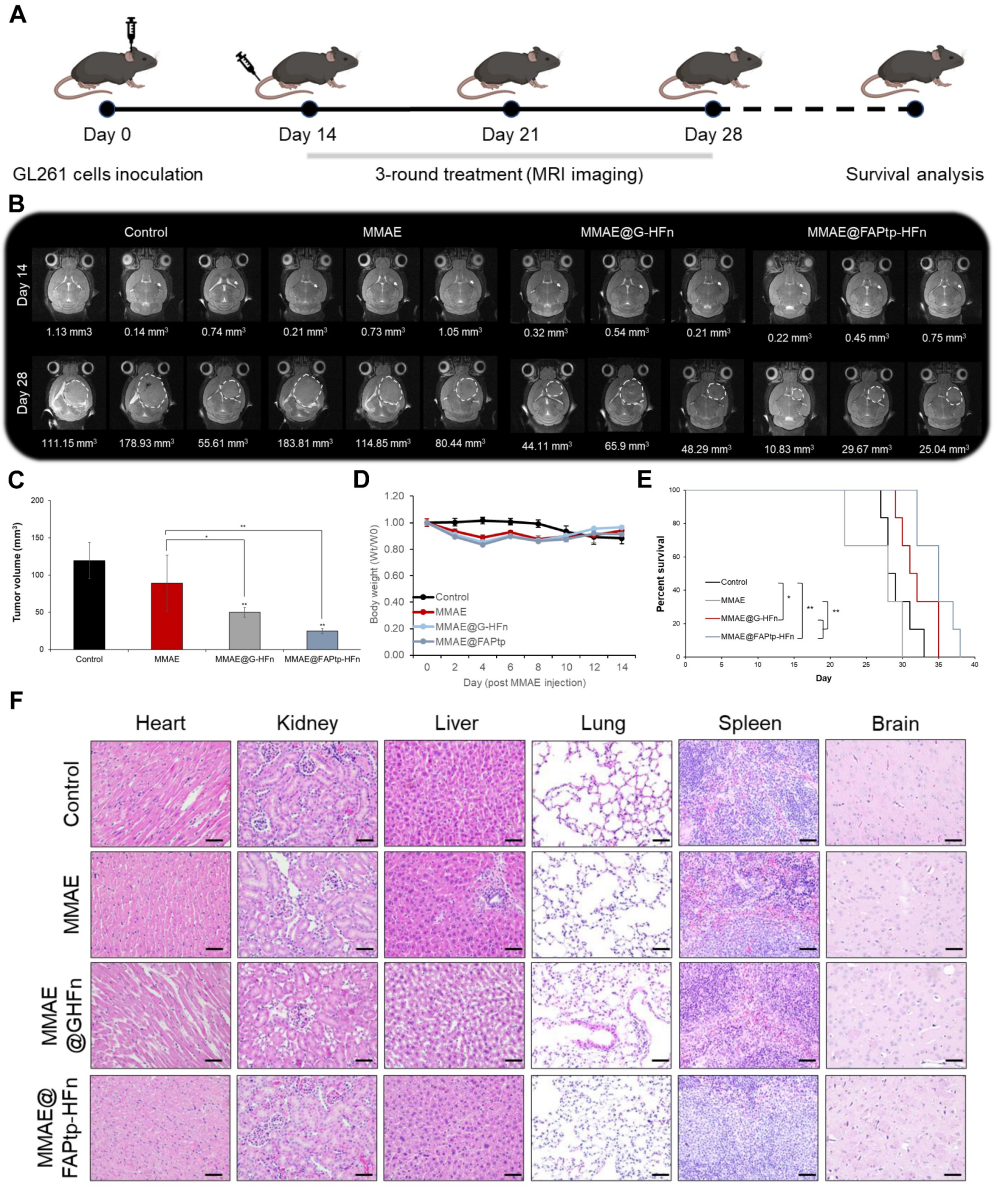
**Evaluation of antitumor efficacy and systemic toxicity in GL261 tumor-bearing mice.** (A) Schematic representation of the study design. GL261 glioma cells were inoculated at Day 0. A 3-round treatment regimen began on Day 14, with MRI monitoring at Days 14 and 28, followed by a survival analysis. (B) Representative T_2_-weighted coronal MRI images of mice brains from four groups: Control, MMAE (free drug), MMAE@G-HFn (non-targeted nanocages), and MMAE@FAPtp-HFn (targeted nanocages). White dashed circles indicate the tumor margins. Numerical values below the images represent the calculated tumor volume in mm^3^. (C) Bar chart showing mean tumor volume at Day 28. The targeted MMAE@FAPtp-HFn group shows a significant reduction in tumor growth compared to all other groups. (D) Body Weight: Line graph tracking relative body weight (W_t_ / W_0_) over 14 days post-injection, showing no significant weight loss, which suggests minimal systemic toxicity. (E) Survival analysis: The percentage of surviving mice over the course of the study is represented via Kaplan-Meier survival curves. The MMAE@FAPtp-HFn treated group exhibits a significantly prolonged survival period compared to the control and free MMAE groups. (F) Representative H&E-stained histological sections of major organs (heart, kidney, liver, lung, spleen, and brain) at the end of the study. No obvious pathological changes or inflammatory infiltrates are observed across the treatment groups, indicating a favorable safety profile. Data are presented as mean ± s.d. Statistical significance was determined using one-way ANOVA followed by Tukey's HSD post hoc test (C) or Kaplan-Meier method followed by the log-rank test (E). *p < 0.05; **p < 0.01.

Prior to therapeutic evaluation of the MMAE-loaded, FAP-targeted ferritin drug carrier, we sought to directly and quantitatively assess intratumoral drug delivery. To this end, liquid chromatography-tandem mass spectrometry (LC-MS/MS) was performed using a stable isotope-labeled internal standard (*d_8_*-MMAE), enabling accurate quantification of MMAE in intracranial tumor tissues. This analysis revealed markedly enhanced drug accumulation with the FAP-targeted ferritin formulation (23.09 ng/g) compared with free MMAE (2.3 ng/g) and the non-targeted ferritin control (8.71 ng/g) (Figure S20). Complementarily, desorption electrospray ionization mass spectrometry imaging (DESI-MSI) was employed to spatially resolve drug distribution within brain sections, while IVIS imaging demonstrated that MMAE@FAPtp-HFn shared a similar biodistribution profile with FAPtp-HFn. Collectively, these results showed strong signals predominantly localized to tumor regions, thereby confirming effective payload delivery to the tumor parenchyma (Figure S21-22).To evaluate the therapeutic potential of the FAPtp-HFn nanocarrier platform *in vivo*, C57BL/6 mice bearing orthotopic GL261 glioma tumors were randomized into three treatment groups: saline control (n = 6), free MMAE (0.15 mg/kg, n = 6), and MMAE@FAPtp-HFn (same concentration as free MMAE, n = 6). All formulations were administered intravenously, and tumor progression was monitored longitudinally using T_2_-weighted magnetic resonance imaging (MRI) (Figure 6A).

By day 28, mice treated with MMAE@FAPtp-HFn showed a significant reduction in both tumor volume (24.80 mm³, n = 6) and tumor growth rate (1.03 mm³/day), compared to the saline group (119.53 mm³; 4.78 mm³/day, n = 6) and the free MMAE group (89.06 mm³; 5.33 mm³/day, n = 6) and MMAE@G-HFn (51.47 mm³; 2.38 mm³/day, n = 6) (Figure 6B and 6C). The high hydrophobicity of MMAE may underlie its poor tolerability and restricted use in its free form, unless combined with antibody or nanoparticle carriers. This inherent property likely results in hepatic metabolism and clearance, with negligible penetration across the BBB, thus preventing effective delivery to tumor sites.

Importantly, systemic toxicity was substantially reduced in the nanocarrier-treated group. Two mice in the early test of high dose MMAE cohort (0.5mg/kg) had to be euthanized early due to > 20% body weight loss after only two doses, whereas mice treated with MMAE@FAPtp-HFn exhibited only transient weight loss, followed by full recovery (Figure 6D).

Survival analysis revealed that mice treated with MMAE@FAPtp-HFn had a median survival of 35 days, compared to 31 days for MMAE@G-HFn and 28.5 days for the untreated group and 28 days for those receiving free MMAE (Figure 6E). To assess potential tissue toxicity, hematoxylin and eosin (H&E) staining was performed on major organs, including the heart, kidney, liver, lung, spleen and brain. In the control group, all examined organs exhibited normal histological architecture without any signs of damage. In the free MMAE group, mild histopathological alterations were observed, including slight myocardial separation, thickening of the renal tubular epithelium, hepatic vacuolization, alveolar wall thickening, and expansion of the splenic white pulp. In the MMAE@FAPtp-HFn group, these toxic effects were markedly alleviated. Most organs retained normal histological architecture with only minimal changes, highlighting the ability of the nanocarrier system to significantly reduce systemic toxicity (Figure 6F).

### Spatial Transcriptomics Uncovers GBM Microenvironmental Remodeling Induced by Treatment

**Figure 7:**
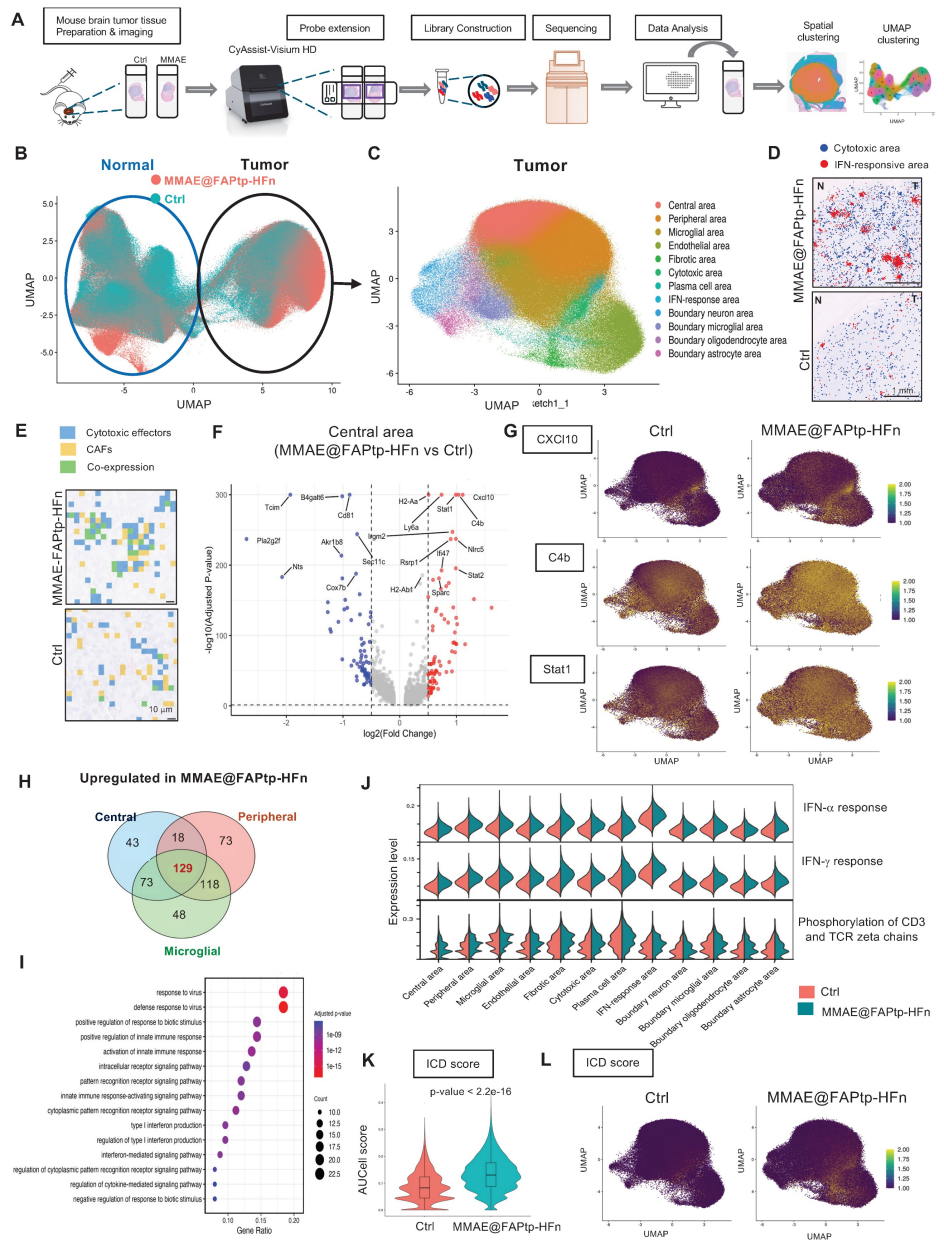
** Spatial transcriptomic analysis reveals the MMAE@FAPtp-HFn treatment-induced remodeling and immune activation in glioblastoma.** (A) Workflow of spatial transcriptomics analysis from tissue collection to data processing and cluster annotation using the 10x Visium HD platform. (B) UMAP plot of integrated spatial transcriptomic data from control and MMAE@FAPtp-HFn-treated brains, with circles indicating normal and tumor regions. (C) Identification of 12 spatial clusters within the tumor region, annotated by anatomical location and gene expression profiles. (D) Spatial distribution of cytotoxic (blue) and IFN-response (red) clusters in control and MMAE@FAPtp-HFn-treated tumors. Treated samples show increased representation of both clusters. 'N' = normal brain; 'T' = tumor. Scale bar: 1 mm. (E) Co-expression map of CAF markers (yellow) and cytotoxic genes (blue), with overlapping regions in green. MMAE@FAPtp-HFn-treated tumors show broader co-expression, suggesting enhanced immune-stromal interaction. Scale bar: 10 µm. (F) Volcano plot of DEGs (treated vs. control) in the central tumor region. Top 15 DEGs are labeled (FDR < 0.05, log₂FC > 0.3). (G) UMAP plots of selected treatment-upregulated genes: CXCL10, C4b, and Stat1. (H) Venn diagram of upregulated DEGs across central, peripheral, and microglia-rich regions. 129 genes are shared, including CXCL10, C4b, and Stat1. (I) Top 15 GO terms enriched among the 129 overlapping upregulated DEGs, highlighting immune-related processes such as viral response and interferon signaling. (J) Violin plots of AUCell scores for immune-related pathways across 12 tumor clusters in control (orange) and treated (green) groups. (K) Violin plots of AUCell-derived ICD scores in control and MMAE@FAPtp-HFn-treated groups. MMAE@FAPtp-HFn-treated samples show significantly higher ICD activity (***p < 0.0001, Wilcoxon test). (L) UMAP visualization of ICD scores, showing spatial enrichment of immunogenic cell death in treated tumors.

To investigate spatial and molecular remodeling in glioblastoma following therapy, we performed spatial transcriptomic profiling using the 10x Genomics Visium HD platform on FFPE brain sections from untreated and treated orthotopic GBM mouse models (Figure 7A). Quality control revealed a reduction in transcript capture within the tumor core, likely due to necrosis, hypoxia-induced stress, or RNA degradation—features consistent with GBM pathology (Figure S23).

Unsupervised clustering and reciprocal PCA integration clearly distinguished tumor and normal brain regions (Figure 7B), with spatial distributions aligning closely to anatomical structures (Figure S24). Within the tumor area, 12 spatially distinct clusters characterized by specific gene expression patterns were identified (Figure 7C and Figure S25), while the normal brain displayed 15 functionally diverse clusters (Figure S26). The MMAE@FAPtp-HFn treatment substantially altered tumor composition (Figure S27): in control tumors, the core occupied 40% of the tumor area, which dropped to 12% post-treatment. Conversely, peripheral tumor areas, microglia-enriched zones, and endothelial-rich regions increased from 32%, 15%, and 8% to 38%, 20%, and 11%, respectively, suggesting structural remodeling and peripheral expansion.

### Spatial Co-Localization of CAFs and Cytotoxic Immune Cells is Enhanced After Treatment

In the MMAE@FAPtp-HFn treated group, two prominent immune-related clusters emerged (Figure 7D). The first cluster expressed cytotoxic T cell markers (*Il2rb, Gzma, Gzmb*) was more condensed in the treated group, suggesting CD8⁺ T cell infiltration penetrating deep into the tumor core (blue spots in Figure 7D). The second displayed strong expressions of interferon-stimulated genes (e.g., *Cxcl10, Usp18, Rsad2, Isg15, Ifit3*) and was broadly distributed within the tumor core and more in the treated group, indicative of Type I interferon signaling (red spots in Figure 7D). Furthermore, spatial co-expression analysis revealed that co-localization of CAFs markers with cytotoxic effectors significantly increased from 13.85% to 30.37% upon treatment (*p* < 0.0001), suggesting that CAFs may play a role in modulating immune cell infiltration and activation (Figure 7E; Figure S28; Table S4).

### Regionalized Gene Expression Changes and Immune Activation

Volcano plots of the DEGs across four major compartments (core, periphery, microglia-rich, endothelial-rich) revealed predominant upregulation of anti-tumor immune and interferon-related genes in the treated group, such as *Cxcl10, C4b*, and *Stat1* (Figure 7F-G; Figure S29). Notably, *Cxcl10* expression in the treated group was approximately twofold higher than in controls, consistent with recent reports of its role in immunomodulation in cholangiocarcinoma 60. The Venn diagram showed 129 overlapping upregulation genes in the treated group from the DEGs in three regions (core, periphery, and microglia-rich) (Figure 7H). GO enrichment analysis of these 129 upregulated genes consistently highlighted immune-related processes, including “response to virus” and “response to interferon-gamma” (Figure 7I), indicating a therapy-induced, viral mimicry-type immune response.

AUCell-based gene set scoring confirmed strong induction of immune-related pathways after treatment, including interferon-α, and interferon-γ responses, as well as phosphorylation of CD3 and TCR zeta chains (Figure 7I; Figure S30). These changes were consistent with the DEGs analysis by upregulation of immune effector genes, collectively establishing a pro-inflammatory, anti-tumor microenvironment (Figure 7I). Since some ICD-related genes were upregulated in the treated group, the AUCell analysis of the ICD score was performed (Figure 7K-L). The results indicated that the treated group had a higher ICD score (*p* < 2.2 e-16). This indicates that while therapy promotes T cell activation and infiltration, converting cold immune into hot immune systems of the GBM environment.

### Treated Tumors Exhibit Enhanced CXCL10 Expression and Potent Cytotoxic T-cell Recruitment

**Figure 8:**
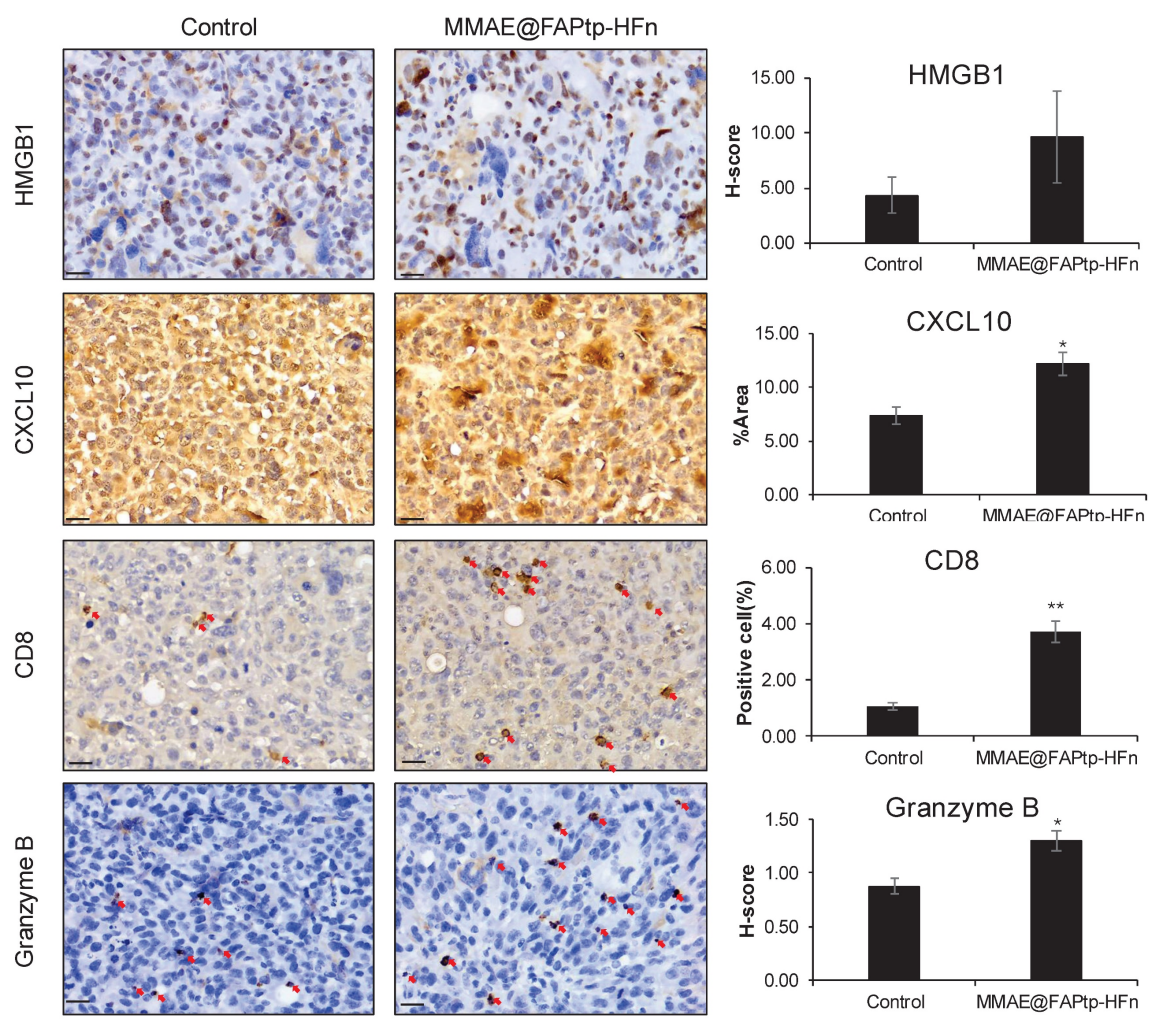
** Immunohistochemical evaluation of immunogenic cell death markers and immune cell infiltration in tumor tissues.** Representative IHC images of tumor sections from the Control and MMAE@FAPtp-HFn-treated groups stained for HMGB1, CXCL10, CD8α, and Granzyme B. Quantitative analyses are presented in the right panels. HMGB1 and Granzyme B expression was evaluated using H-score; CXCL10 was quantified by the percentage of positive area (%Area); and CD8α was quantified by the percentage of positive cells. Red arrows indicate positive staining for CD8α (cytotoxic T cells) and Granzyme B (cytotoxic lymphocytes). Scale bars: 20 μm. Data are presented as mean ± s.e.m. (n = 3). Statistical significance was determined using an unpaired two-tailed Student's t-test. *p < 0.05 and **p < 0.01 versus the Control group.

Before to evaluating the therapeutic mechanism, we validated the expression of the target antigen in our orthotopic model. Immunohistochemical (IHC) analysis confirmed that FAP is significantly upregulated in the GL261 and U-87MG tumor region, particularly at the invasive margin, compared to normal brain tissue (Figure S31-32). It is well-established that MMAE induces apoptosis, which can subsequently trigger ICD 61. To validate our spatial transcriptomics findings, we first assessed the expression of the chemoattractant *Cxcl10*. Consistent with the DEG analysis, immunohistochemical (IHC) staining confirmed a significant expansion of the CXCL10+ area (1.63-fold increase compared to controls,* p* < 0.05; Figure 8). This elevation in chemoattractant signaling was accompanied by a robust induction of immune-related pathways. The recruitment of effector cells was further quantified via CD8α IHC staining. In alignment with our transcriptomic data, the treatment group exhibited a significant increase in cytotoxic T cell infiltration, rising from a baseline of 1.0% in the control group to 3.7% in the treated group (*p* < 0.01; Figure 8). To evaluate the functional status of these recruited cells, we measured granzyme B levels, which were 1.49-fold higher in the treated tumors compared to controls (*p* < 0.05), indicating that the infiltrating T cells possessed potent cytotoxic activity.

Mechanistically, we investigated the role of ICD in driving this immune remodeling. AUCell analysis revealed significantly higher ICD scores in the treated group, a finding supported by the observed upregulation of cytoplasmic HMGB1—a hallmark DAMP (damage-associated molecular pattern) released during ICD. Taken together, these results demonstrate that the therapeutic intervention triggers ICD and subsequent HMGB1/CXCL10 signaling, which facilitates the recruitment and activation of functional cytotoxic T cells within the tumor microenvironment, likely serving as a primary driver of the observed therapeutic efficacy.

## Discussion

Traditional antibody-drug conjugates (ADCs) often employ random conjugation to lysine or cysteine residues, producing heterogeneous products with variable drug-to-antibody ratios (DARs) 62. This variability results in inconsistent pharmacokinetics and therapeutic outcomes 63. In contrast, site-specific conjugation techniques, such as cysteine rebridging, enzyme-mediated reactions, and click chemistry, have advanced ADC technology by enabling precise payload attachment at specific antibody sites, achieving uniform DARs (typically 2 or 4) 64. These advancements have revitalized ADCs as a potent platform for targeted cancer therapy by combining antibody specificity with cytotoxic agents. However, challenges remain, including limited drug-loading capacity, linker cleavage efficiency, premature payload release causing off-target toxicity, and antigen heterogeneity reducing efficacy 65.

To address these issues, FDCs have emerged as a promising alternative. Ferritin's natural nanocage structure supports high drug (as its free form) encapsulation efficiency and controlled release, making it an ideal platform for cancer therapy 47. In this study, a modular and programmable FDC platform was developed for glioblastoma-targeted imaging and therapy. Sortase A-mediated ligation was used to precisely attach synthetic FAP-binding peptides to the N-terminus of HFn nanocages, yielding a platform with robust tumor specificity, high drug payload capacity, biocompatibility, and adaptability for further modifications 55.

A critical element of this work is the optimized FAP-targeting PET tracer ligand, DOTA-Alb-FAPtp-02, which exhibited enhanced tumor accumulation. Additionally, a FAP-specific cleavable linker was integrated into the prodrug FAPtp-DOX to enable selective drug release in the tumor microenvironment. While PET imaging confirmed efficient tumor uptake and favorable pharmacokinetics, therapeutic efficacy was suboptimal, with tumor relapse occurring soon after treatment cessation. Potential reasons include incomplete enzymatic cleavage, steric hindrance from the bulky drug structure, or limited tissue penetration. These results highlight the challenges of tumor microenvironment-activated prodrugs and the need for improved linker design and pharmacodynamic optimization.

To improve outcomes, the FDC variant MMAE@FAPtp-HFn, incorporating a fluorinated FAP-binding peptide and encapsulated MMAE, was developed. This construct showed excellent structural stability, pH-responsive drug release, and enhanced tumor accumulation via dual targeting of FAP and TfR1. Traditionally, TfR1 has been recognized primarily for its role in mediating iron uptake on the cell surface. However, recent evidence by Hou et al. revealed that TfR1 can translocate to the nucleus, where it interacts with the tumor suppressor p53 and enhances the transcription of genes involved in nucleotide excision repair (NER) 66. This nuclear function promotes tumor cell survival and contributes to chemoresistance. Our dual-targeting FDCs strategy, which leverages both FAP and TfR1 targeting, may thus provide a more effective therapeutic approach by exploiting TfR1 overexpression and its nuclear regulatory functions to enhance drug efficacy and overcome treatment resistance. In orthotopic glioma models, it achieved superior tumor suppression, extended survival, and reduced systemic toxicity compared to free MMAE. This formulation's therapeutic profile underscores the potential of structurally defined, receptor-mediated nanocarriers for effective and selective glioma therapy.

It is noteworthy that Chen et al. recently developed a dual-targeting ferritin nanoplatform (HFAI, mHFn-FAPI@ATMi/ICG) to enhance immunotherapy in CAF-rich colorectal cancer 67. While their design focused on photothermal/photodynamic modulation of the tumor microenvironment, our approach differs substantially in both molecular engineering and application. Specifically, we introduce a sortase A-mediated site-specific conjugation of an optimized FAP-targeting ligand onto ferritin, achieving precise control of ligand density while preserving TfR1-binding affinity. This strategy provides a distinct advantage for targeted chemotherapy and theranostic applications in glioblastoma.

Because recombinant human ferritin was administered repeatedly in an immunocompetent murine glioma model, a xenogenic antibody response was anticipated and was indeed detected (Figure S33). Importantly, this immune recognition was not associated with overt toxicity or systemic adverse effects. Treated animals exhibited stable body weight, normal hepatic and renal function (Table S6), and no abnormal behavior. Pro-inflammatory cytokine analysis (Table S7) further supported systemic safety, as serum TNF-α remained low across all groups and IL-6 levels in the MMAE@FAPtp-HFn group were maintained at or below baseline levels, contrasting with the variability observed in non-targeted formulations. The favorable inflammatory profile is consistent with the low endotoxin content (0.5 EU/mg) of the formulation and effective tumor burden reduction. While these findings indicate good short-term immunological tolerability, especially in a cross-species setting, more comprehensive long-term immunogenicity studies will be required to support clinical translation.

The proposed FDC platform's modularity allows for flexible exchange of ligands and payloads, supporting compatibility with various therapeutic agents and targeting strategies. Compared to traditional ADCs and peptide-drug conjugates, sortase-based ferritin engineering offers a unique combination of biochemical precision, drug capacity, and biological performance.

The spatial transcriptomic analysis further reveals that FAP targeted therapy induces profound structural and immunological remodeling within the glioblastoma tumor microenvironment. One of the most notable changes is the reduction in tumor core dominance and a proportional increase in peripheral and stromal regions, reflecting both direct cytotoxic effects and compensatory spatial reorganization. This remodeling is accompanied by distinct region-specific transcriptomic changes, where gene downregulation in the core likely reflects tumor cell death or necrosis, while immune-related pathways, particularly interferon signaling and inflammatory gene expression, are upregulated in the periphery and vascular zones, suggesting heightened immune activity at these interfaces. Contrary to the traditional model of immune exclusion from the tumor center, we observed widespread activation of interferon-stimulated genes (ISGs) and deep infiltration of cytotoxic T cells into the tumor mass, indicating that therapy may overcome spatial immune barriers and facilitate immune cell access to formerly protected tumor regions. Validated by our protein-level analysis (Figure 8), the upregulation of HMGB1 and CXCL10 indicates an ICD response that actively recruits immune effectors into the tumor microenvironment. Importantly, the significant elevation of granzyme B within these CD8^+^ infiltrates demonstrates that the recruited cells are functionally competent executioners, directly contributing to tumor suppression via granule-mediated cytotoxicity. Additionally, therapy disrupted the spatial association between endothelial and fibrotic compartments, potentially pointing to vascular remodeling or fibrotic compartmentalization, which could inform future vascular- or stroma-targeted treatment strategies. Targeting CAFs via nanoparticle delivery not only reduced tumor burden and prolonged survival but also reprogrammed the tumor microenvironment toward a more immunogenic state. CAF-rich regions showed increased co-localization with cytotoxic immune cells and upregulation of cytolytic effector and interferon-related genes, suggesting that CAFs can be therapeutically reconditioned to promote antitumor immunity. Enhanced activation of T cell receptor signaling and cytokine-mediated pathways further supports the restoration of T cell function within the tumor. However, the concurrent upregulation of PD-1 signaling in treated tumors suggests the emergence of an adaptive resistance mechanism, wherein increased immune activation drives compensatory immune checkpoint expression. These findings underscore the potential of combining FAP-targeted therapy with immune checkpoint blockade to boost immune activation while counteracting resistance, thereby improving therapeutic efficacy in glioblastoma 68-70.

## Conclusion

In all, a rationally designed, site-specific ferritin-drug conjugation system has been developed for FAP-targeted therapy, offering clear advantages over existing peptide-drug conjugates and ADCs. This FDC platform provides a structurally tunable and functionally versatile approach to address the biological and delivery challenges of complex tumor microenvironments. However, limitations require further exploration. The variability of FAP expression across glioma subtypes calls for broader biological validation. The immunogenicity and long-term toxicity of ferritin-based constructs need evaluation in extended models. Ongoing research is investigating scalability, immune responses, and resistance mechanisms, with potential applications extending beyond glioma and in combination with other therapies.

## Supplementary Material

Supplementary materials and methods, figures and tables.

## Abbreviations

ADC: antibody-drug conjugate
BBB: blood-brain barrier
CAF: cancer-associated fibroblast
CNS: central nervous system
DAMP: damage-associated molecular pattern
DEG: differential gene expression
eSrt A: evolved sortase A
FAP: fibroblast activation protein
FDC: ferritin-based drug carrier
FITC: fluorescein isothiocyanate
GBM: glioblastoma multiforme
HFn: human ferritin heavy chain 1
ICD: immunogenic cell death
IHC: immunohistochemical
MMAE: monomethyl auristatin E
TEM: transmission electron microscopy
TfR1: transferrin receptor 1
