# Supplementary material for: Engineering a modular FAP-targeting ferritin-based drug nanocarrier for enhanced glioblastoma theranostics
Source: Theranostics. 2026 Jan 21;16(8):4169–89. doi: 10.7150/thno.125403 (PMC12905753; doi:10.7150/thno.125403)
Supplement: Supplementary file 1 — Supplementary materials and methods, figures and tables. [file thnov16p4169s1.pdf]

Supplementary Material

Engineering a modular FAP-targeting ferritin-based drug nanocarrier for enhanced glioblastoma theranostics

Yi-Hsiang Tseng[1], Jia-Yu Lin[1], Chia-Pao Chuang[1], Hsiao-Ching Su[1], Teh-Wei Wang[2], Kuo-Chen Wei[34], Feng-Ting Huang[1*], and Chiun-Wei Huang[5*]

1. Department of Biochemical Science & Technology, National Taiwan University, Taipei, 106319 Taiwan
2. Project Division of Generative AI Utilization Aging Cells, The Institute of Medical Science, The University of Tokyo, Tokyo, Japan
3. Neuroscience Research Center and Department of Neurosurgery, Linkou Chang Gung Memorial Hospital, Taoyuan, 33305 Taiwan
4. School of Medicine, Chang Gung University, Taoyuan, 33302 Taiwan
5. Department of Medical Research and Development, Linkou Chang Gung Memorial Hospital, Taoyuan, 33305 Taiwan

*Corresponding authors: Feng-Ting Huang (fthuang@ntu.edu.tw) and Chiun-Wei Huang (steincafe77@gmail.com)

Supplement materials and methods

Determination of Octanol-Water Partition Coefficient

To evaluate the lipophilicity of the developed peptide tracers, the octanol-water partition coefficient (log P) was determined. Briefly, the radiotracer (~111 kBq) was diluted in 500 µL of phosphate-buffered saline (PBS, pH 7.4) and mixed with an equal volume of 1-octanol (500 µL) in a microcentrifuge tube. The mixture was vigorously vortexed for 1 min at room temperature, followed by centrifugation at 12,500 rpm for 5 min to ensure phase separation. From both the aqueous (PBS) and organic (octanol) layers, 200 µL volumes were isolated. The radioactive content of these fractions was then quantified using a Cobra II gamma counter (Packard Instruments, Downers Grove, IL). All experimental trials were conducted in triplicate.

Cell Lysis and Protein Extraction

Cells were harvested and lysed in radioimmunoprecipitation assay (RIPA) buffer (25 mM Tris-HCl, pH 7.6, 150 mM NaCl, 0.5% NP-40, 1% sodium deoxycholate, and 0.1% SDS) supplemented with Halt™ protease and phosphatase inhibitor cocktail (Thermo Fisher Scientific, Waltham, MA, USA). After incubation on ice for 30 min, the lysates were centrifuged at 4 °C to remove cell debris. The supernatant was collected, and the protein concentration was quantified using the DC™ Protein Assay (Bio-Rad, Hercules, CA, USA). Subsequently, the protein samples were mixed with SDS loading buffer, denatured at 100 °C for 15 min, and stored at −20 °C until further analysis.

Western Blot Analysis

Cell lysates were resolved by 10% sodium dodecyl sulfate-polyacrylamide gel electrophoresis (SDS-PAGE) and transferred onto polyvinylidene difluoride (PVDF) membranes. The membranes were blocked with 5% skim milk in phosphate-buffered saline containing 0.5% Tween-20 (PBST) for 1 h at room temperature. Subsequently, the membranes were incubated overnight at 4 °C with primary antibodies against FAP (1:1000; ab207178, Abcam, Cambridge, UK), TfR1 (1:1000; #46222, Cell Signaling Technology, Danvers, MA, USA), GAPDH (1:10,000; #2118, Cell Signaling Technology), and α-tubulin (1:5000; ab7291, Abcam). Following three washes with PBST, the membranes were incubated with the corresponding horseradish peroxidase (HRP)-conjugated secondary antibodies for 1 h at room temperature. Protein bands were visualized using Pierce™ ECL Western Blotting Substrate (Thermo Fisher Scientific, Waltham, MA, USA) and captured using the Biospectrum Imaging System.

Albumin Binding Assay

To determine the albumin-binding capability of the radiotracer ^{68}Ga-Alb-FAPtp-01, human serum albumin (HSA; Sigma-Aldrich, St. Louis, MO, USA, A1653) was utilized. Briefly, HSA was serially diluted (two-fold) in phosphate-buffered saline (PBS: 10 mM Na_2HPO_4, 1.8 mM KH_2PO_4, 137 mM NaCl, 2.7 mM KCl, pH 7.4) supplemented with Tween-20. The diluted HSA samples were mixed with ^{68}Ga-Alb-FAPtp-01 (37 kBq/mL) at 37 °C for 30 min. Following incubation, the albumin-bound fraction was separated from the free radiotracer using a desalting column. The radioactivity associated with the high-molecular-weight fraction (albumin-bound) was quantified using a gamma counter (Pharmacia/LKB Nuclear, Inc.). Data were expressed as the percentage of protein-bound radioactivity relative to the total incubated dose.

In vivo Evaluation of Therapeutic Efficacy

Xenograft tumor models were established by implanting human glioma U-87 MG cells into the right flank of female athymic BALB/c nude mice (6–8 weeks old), as described above. When the average tumor volume reached approximately 100 mm^3, mice were randomly divided into treatment groups (n = 5 per group). The mice were intravenously injected via the lateral tail vein with either the FAPtp-DOX conjugate or free DOX at a DOX-equivalent dose of 250

nmol/kg, following the schedule outlined in Figure S6B. The control group received an equivalent volume of sterile saline (0.9% NaCl). Tumor dimensions and body weights were monitored twice weekly. Tumor volume (V) was calculated using the formula: $V = 0.5 \times \text{length} \times \text{width}^2$. To monitor therapeutic responses and tumor progression, dual-modality PET/CT imaging was performed at designated time points. Animals were euthanized if humane endpoints were reached, such as a weight loss greater than 15%, in accordance with the approved institutional animal care guidelines. Statistical analysis of treatment efficacy was performed using two-way ANOVA (GraphPad Prism 6).

DESI Mass Spectrometry Imaging (DESI-MSI)

Tumor-bearing mouse brains were harvested, embedded in carboxymethyl cellulose (CMC), and snap-frozen at -80 °C. The frozen tissue blocks were cryosectioned at a thickness of 10 µm using a cryostat (Leica Biosystems) and thaw-mounted onto glass slides. DESI-MSI analysis was performed using a Cyclic IMS™ mass spectrometer equipped with a DESI XS source. Mass spectrometry analysis was performed in positive ionization mode, scanning a mass-to-charge ratio (m/z) from 500 to 800. A solvent mixture comprising methanol/water/formic acid (98:2:0.01, v/v/v) was delivered at a flow rate of 0.25 µL/min. The nitrogen nebulizing gas pressure was set to 0.1 MPa, and the source temperature was maintained at 100 °C. Images were acquired at a spatial resolution of 50 µm with a scan speed of 0.33 s per pixel. Leucine enkephalin was used as the internal standard (lock mass) for real-time mass correction.

LC-MS/MS Method

Samples (10 µL) were injected into a Vanquish Flex UHPLC system (Thermo Fisher Scientific) equipped with an Agilent ZORBAX RRHD Eclipse Plus C18 column (1.8 µm, 2.1 × 100 mm). Chromatographic separation was executed at 0.4 mL/min using a mobile phase system of 0.1% formic acid in water (A) and acetonitrile (B). A linear gradient was applied, increasing the concentration of B from 1% to 98% over a 5-minute duration. Mass spectrometric detection was subsequently performed on a Stellar instrument (Thermo Scientific) utilizing positive electrospray ionization (ESI+). The method employed Full MS scanning (m/z 70–1050) followed by CID fragmentation (NCE 35%) to identify MMAE.

Serum Biochemistry and Cytokine Analysis

Blood Collection and Serum Preparation

Following the experimental period, blood was obtained from anesthetized subjects via terminal cardiac puncture and collected into tubes devoid of any anticoagulant. The samples were allowed to clot at room temperature for 30 minutes and then centrifuged at 3,000 g for 15 minutes at 4°C to separate the serum. The obtained serum was stored at -80°C until further analysis.

Serum Biochemical Analysis

To evaluate systemic toxicity, hepatic and renal functions were assessed by measuring serum biochemical parameters. Hepatotoxicity was determined by quantifying alanine aminotransferase (ALT) and aspartate aminotransferase (AST) levels. Kidney function was evaluated by measuring blood urea nitrogen (BUN) and creatinine (Cre) levels. All parameters were analyzed using a SPOTCHEM EZ SP-4430 automated biochemical analyzer (ARKRAY Inc., Kyoto, Japan). Serum samples were applied to SPOTCHEM II reagent strips, and analytes were quantified via reflectance photometry in accordance with the manufacturer's instructions. System accuracy was maintained through automated calibration and quality control using lot-specific calibration cards provided by the manufacturer.

Cytokine Analysis

Systemic inflammatory responses were evaluated by measuring the serum concentrations of tumor necrosis factor-alpha (TNF-α) and interleukin-6 (IL-6). The levels of these cytokines were quantified using ELISA MAX™ Standard Set Mouse TNF-α/IL-6 kits (BioLegend, San Diego, CA, USA) following the manufacturer's instructions. Briefly, serum samples were added to a 96-well plate pre-coated with specific capture antibodies and incubated for 2 hours at room temperature. After washing, a detection antibody was added, followed by the addition of a streptavidin-HRP solution. Following development with a substrate solution, absorbance was measured at 450 nm using a SpectraMax i3x microplate reader (Molecular Devices, San Jose, CA, USA). The concentrations of TNF-α and IL-6 were calculated based on standard curves generated from recombinant standards provided in the kits.

Assessment of Immunogenicity by ELISA

Indirect ELISA was performed to analyze the levels of anti-HFn antibodies in mouse serum. Briefly, 96-well high-binding plates were coated with recombinant HFn protein (2 μg/mL) in carbonate-bicarbonate buffer (pH 9.6) and incubated overnight at 4 °C. The plates were washed three times with PBS containing 0.05% Tween-20 (PBST) and blocked with PBS contain 1% BSA for 2 h at room temperature. Serum samples were diluted at 1:2000 in blocking buffer. A commercial mouse monoclonal anti-ferritin heavy chain antibody (clone B-12, sc-376594, Santa Cruz Biotechnology, Dallas, TX, USA) diluted at 1:500 served as the positive control, blocking buffer was used as the blank. Diluted samples (100 μL/well) were incubated for 2 h at room temperature. Following three PBST washes, horseradish peroxidase (HRP)-conjugated secondary antibody was added at a dilution of 1:20000 and incubated for 1 h at room temperature. After washing four times, 3,3',5,5'-tetramethylbenzidine (TMB) substrate (100 μL/well) was added. The reaction was developed for 5–15 min in the dark and stopped by the addition of 2N HCl (100 μL/well). Absorbance at 450 nm (OD450) was measured using a microplate reader.

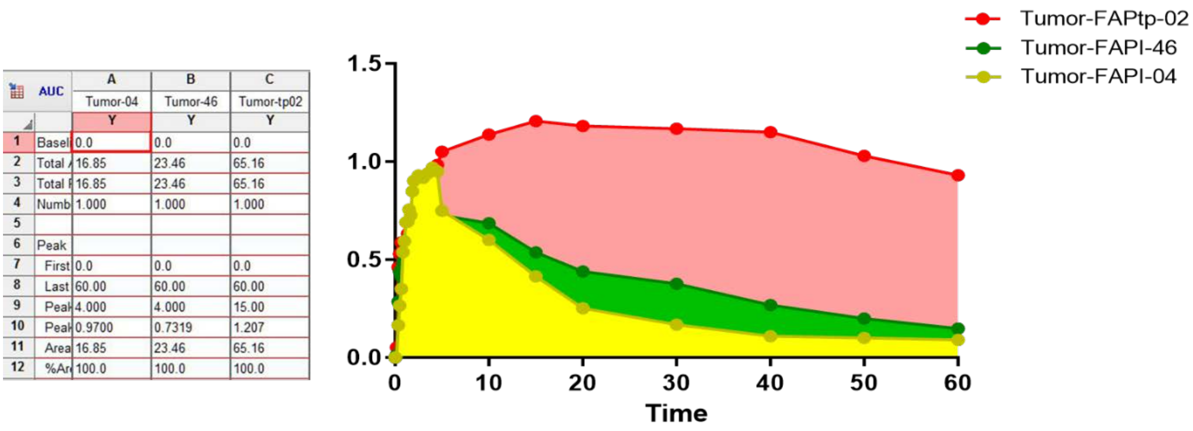

AUC	A	B	C
	Tumor-04	Tumor-46	Tumor-tp02
	Y	Y	Y
1 Basel	0.0	0.0	0.0
2 Total /	16.85	23.46	65.16
3 Total I	16.85	23.46	65.16
4 Numb	1.000	1.000	1.000
5			
6 Peak			
7 First	0.0	0.0	0.0
8 Last	60.00	60.00	60.00
9 Peal	4.000	4.000	15.00
10 Peal	0.9700	0.7319	1.207
11 Area	16.85	23.46	65.16
12 %Ar	100.0	100.0	100.0

Figure S1. *In vivo* dynamic PET imaging analysis compares the time-activity curves of FAP tracers for U-87 MG tumors and the activity coverage areas of each tracer.

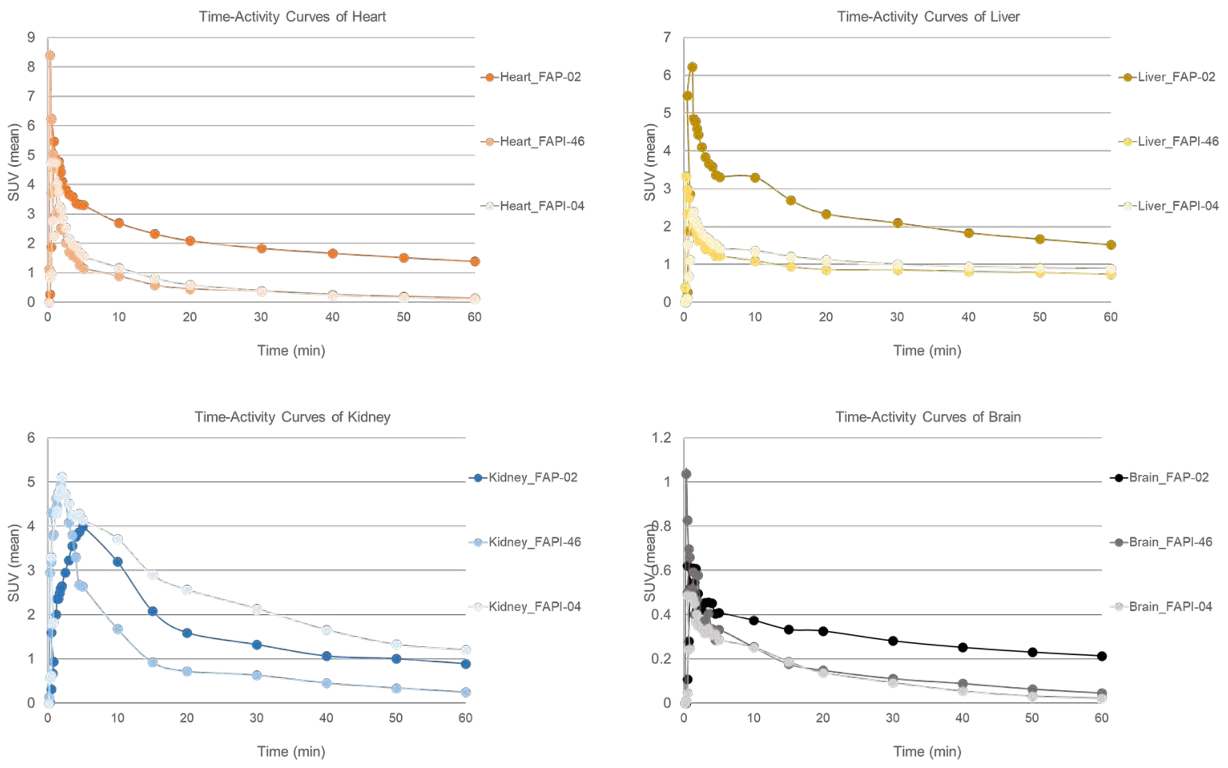

Figure S2. Comparison of the time-activity curves of and ^{68}Ga-Alb-FAPtp-02, ^{68}Ga-FAPI-46 and ^{68}Ga-FAPI-04 in major organs based on ROIs drawn from dynamic imaging data.

Proteomic analysis experiments

HPLC Analysis Report

Peptide ID: 992589

Name: FAPtp-DOX

Sequence: DOTA-Lys{4-(p-chlorophenyl)butyric acid)}-PEG8-Trp-Gly-Pro(4,4difluoro)-linker-(Doxorubicin)

Mobile Phase:

Solvent A: 0.1%TFA in 100%water

Solvent B: 0.09%TFA in (80%ACN+20%H20)

Flow: 1.0ml/min 48.0%-58.0% B buffer in 20min

Wavelength: 220nm

Column: PLRP-S 8u 300A 4.6*250mm A1389#

Volume: 10ul

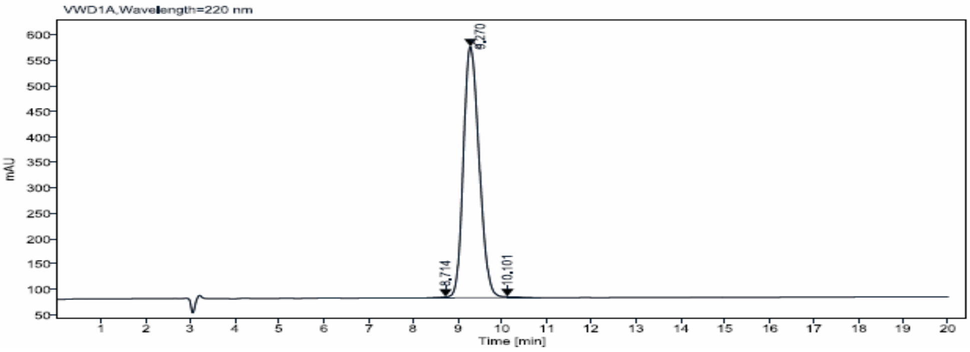

Signal: VWD1A,Wavelength=220 nm

RT [min]	Type	Width [min]	Height	Area	Area%
8,714	BV	1,120	1,718	26,007	0,213
9,270	VF	1,387	493,053	12154,346	99,496
10,101	VBA	0,721	1,986	35,509	0,291
			Sum	12215,8626	

MS Analysis Report

Peptide ID: 992589

Name : FAPtp-DOX

Sequence: DOTA-Lys{4-(p-chlorophenyl)butyric acid)}-PEG8-Trp-Gly-Pro(4,4difluoro)-linker-(Doxorubicin)

Lot No.: PEPC13399

MW.: 2309.90

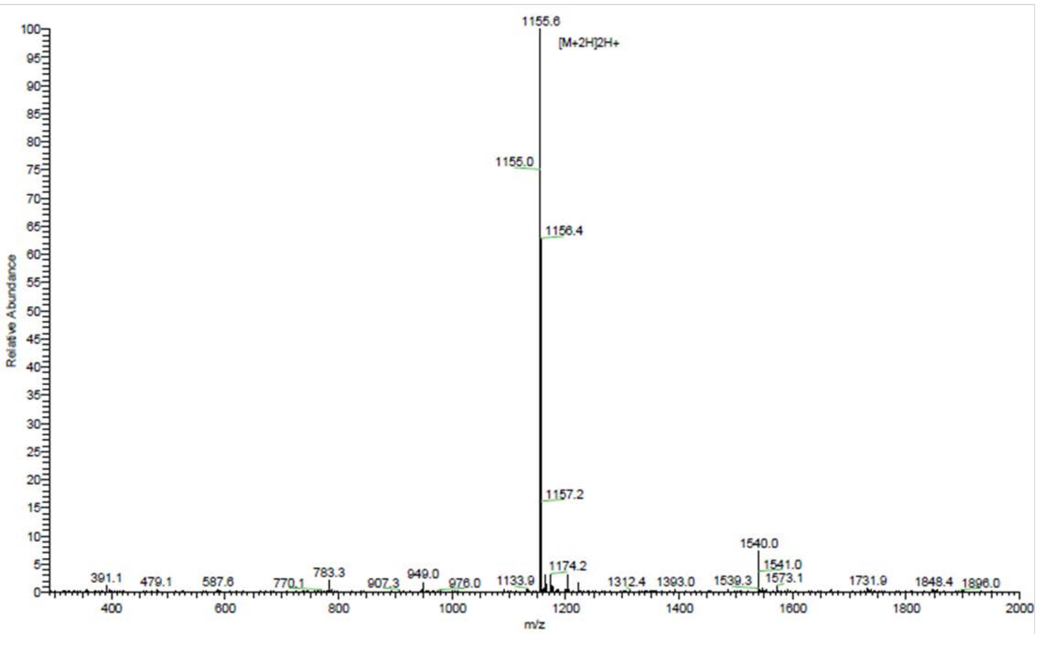

In vivo **PET/CT imaging and quantitative analysis of ^{68}Ga-Alb-FAPtp-DOX and ^{68}Ga-Alb-FAPtp-02**

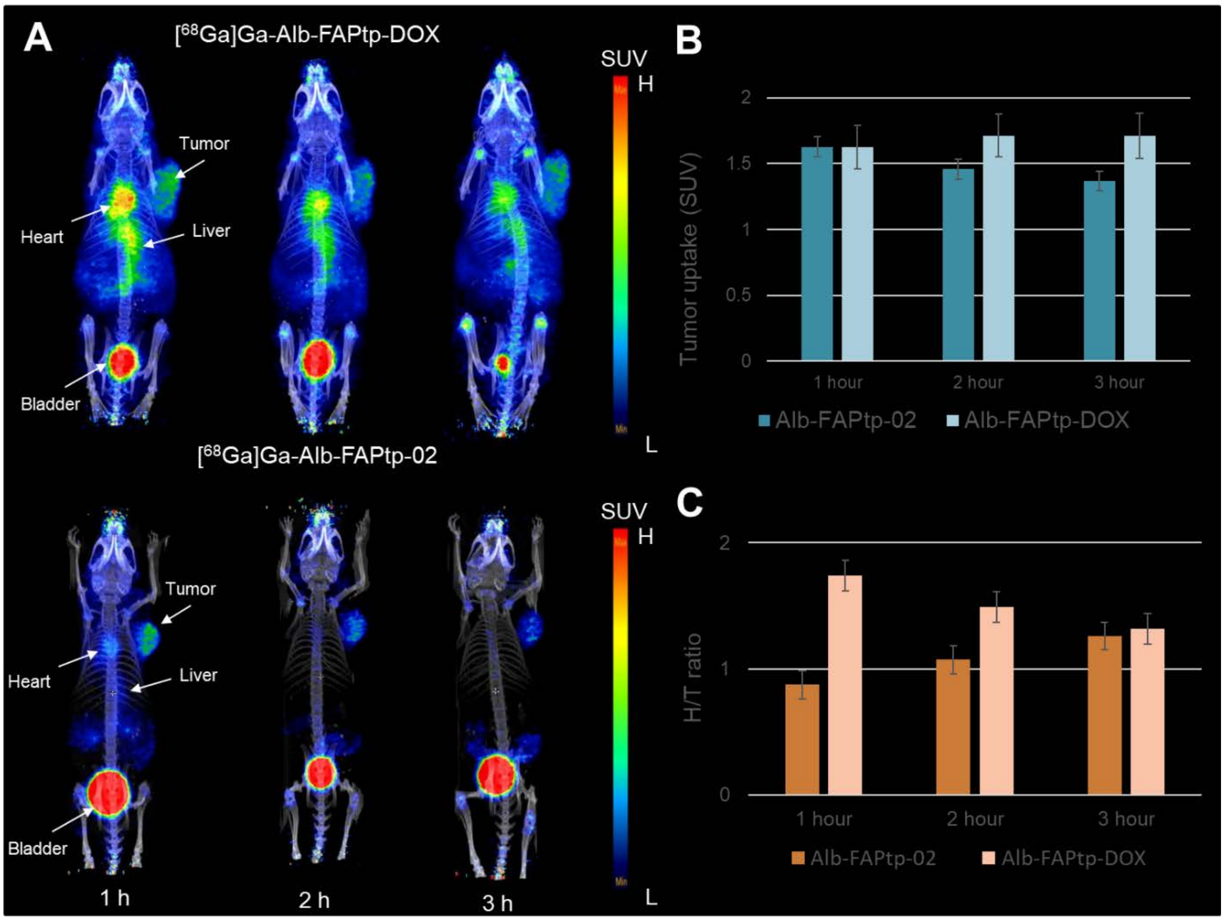

Figure S3. ***In vivo*** **PET/CT imaging and quantitative analysis of ^{68}Ga-Alb-FAPtp-DOX and ^{68}Ga-Alb-FAPtp-02 in tumor-bearing mice.** (A) Representative Micro-PET/CT Images: Coronal PET/CT images of mice at 1 h, 2 h, and 3 h post-injection of ^{68}Ga-Alb-FAPtp-DOX (top row) and ^{68}Ga-Alb-FAPtp-02 (bottom row). White arrows indicate the accumulation of the radiotracer in the tumor, heart, liver, and bladder. The color scale represents the Standardized Uptake Value (SUV), ranging from low (blue) to high (red). (B) Tumor Uptake: Quantitative analysis of tracer accumulation in the tumor expressed as SUV at 1, 2, and 3 h post-injection. Both tracers show sustained tumor uptake, with ^{68}Ga-Alb-FAPtp-DOX showing slightly higher retention at later time points. (C) Heart-to-Tumor (H/T) Ratio: Comparison of the H/T ratios for both compounds over time. The ^{68}Ga-Alb-FAPtp-DOX tracer exhibits a higher H/T ratio, particularly at the 1 h and 2 h marks, indicating higher blood pool activity compared to the 02 variant. Data are presented as mean ± s.d. (n = 3).

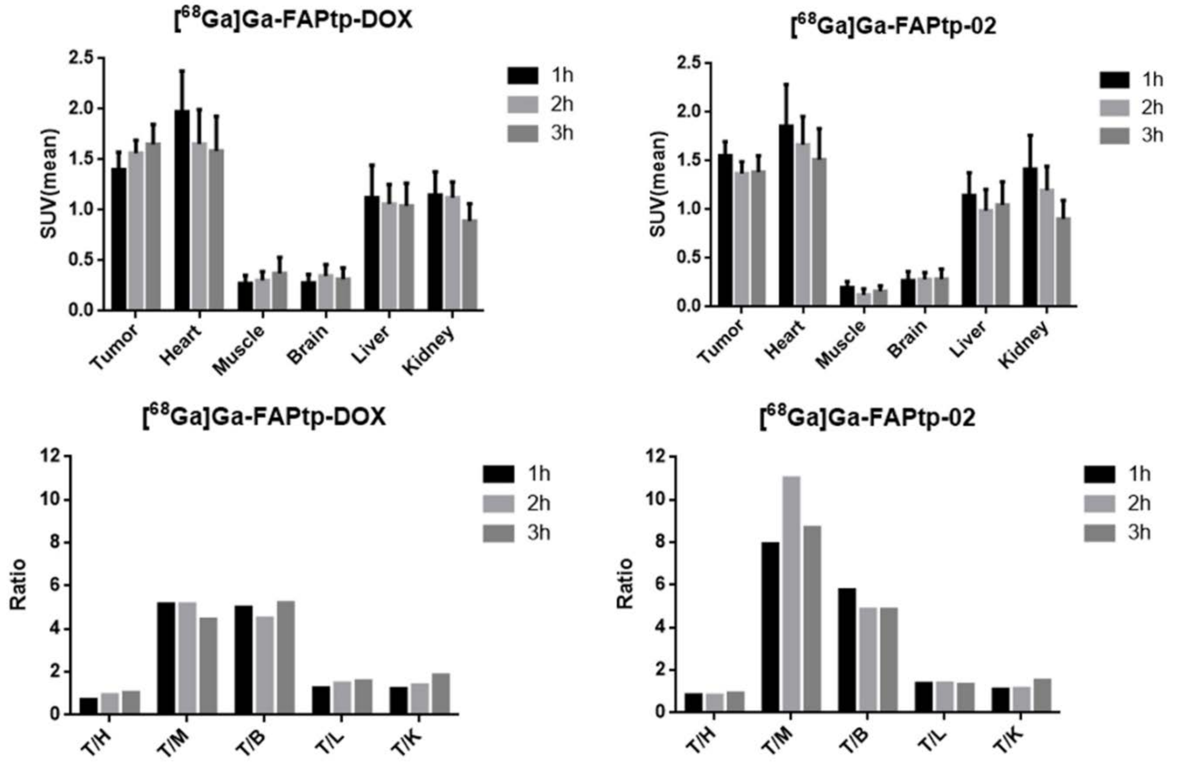

Figure S4. Quantitative analysis of ^{68}Ga-Alb-FAPtp-DOX and ^{68}Ga-Alb-FAPtp-02 in major organs.

Proteomic analysis experiments

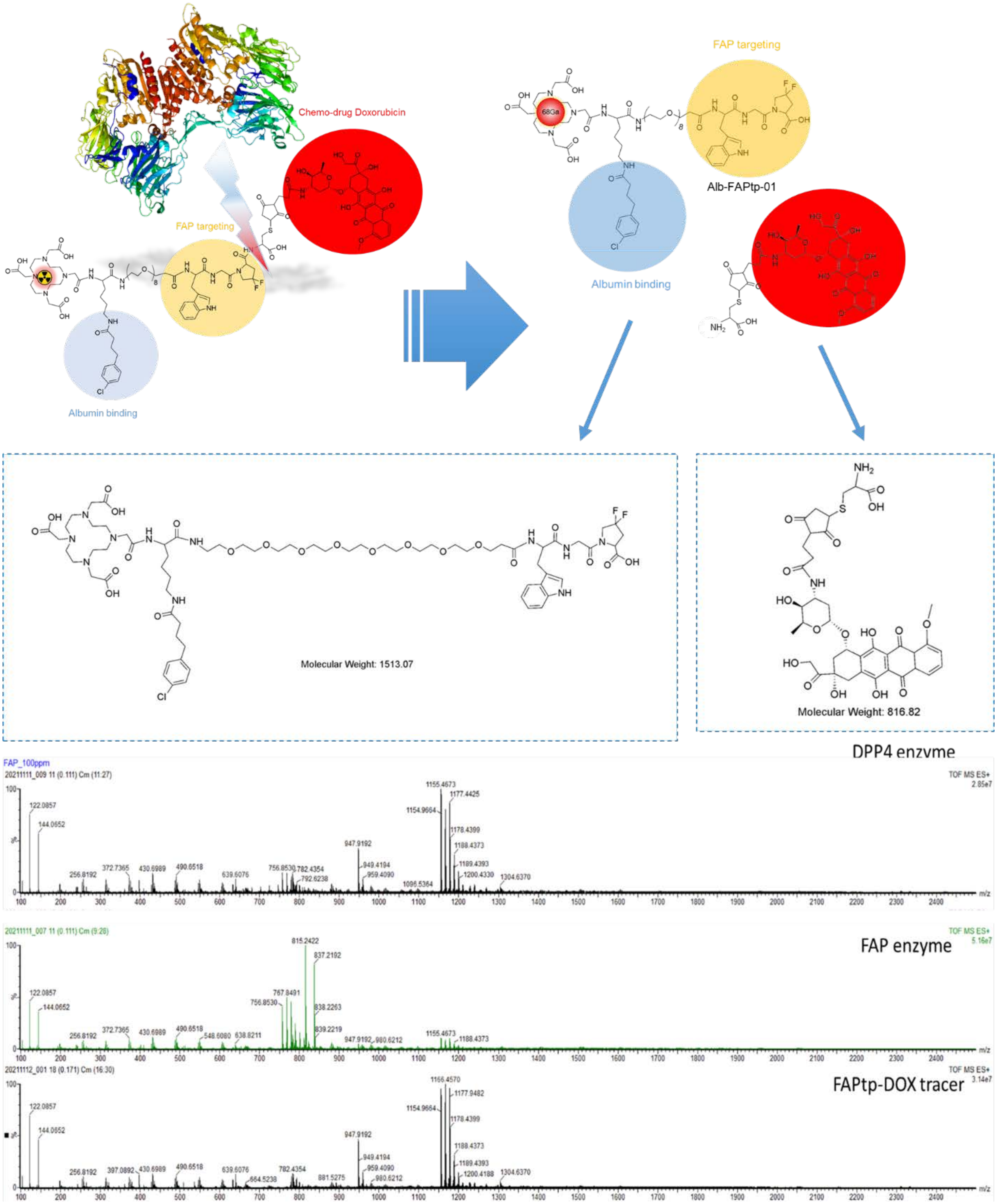

Figure S5. Proteomic analysis of cleavage specificity. Mass spectrometry results demonstrate that the FAPtp-DOX tracer is specifically cleaved into two fragments by the FAP enzyme, whereas no cleavage occurs with DPP4.

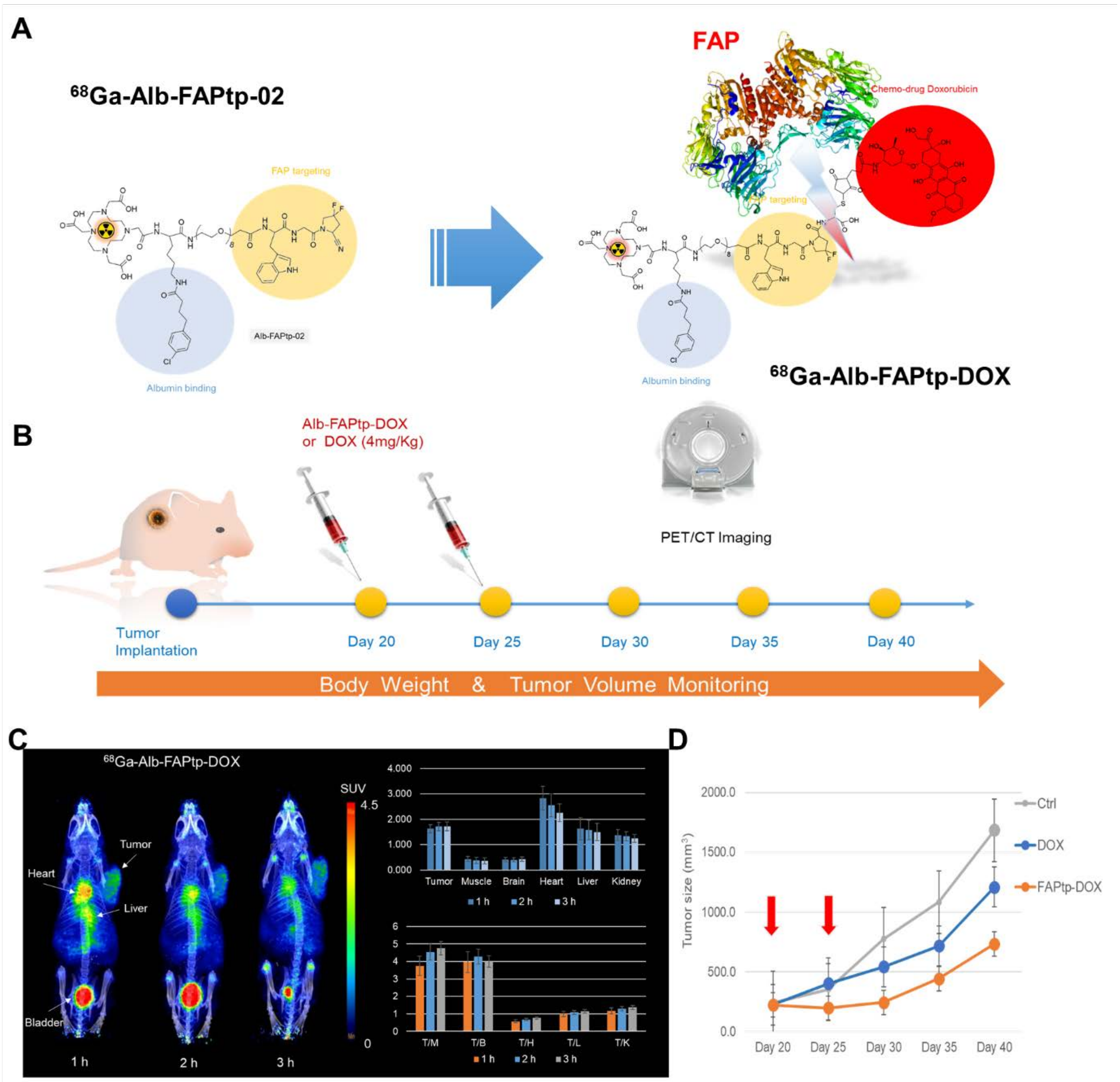

Figure S6. **Characterization, workflow, and *in vivo* evaluation of ⁶⁸Ga-Alb-FAPtp-DOX**. (A) Schematic illustration of the theranostic agent ⁶⁸Ga-Alb-FAPtp-DOX, in which the chemotherapeutic agent doxorubicin (DOX) is conjugated to a FAP-targeting scaffold for targeted drug delivery and PET imaging. (B) Schematic overview of the in vivo experimental design. Following tumor implantation, mice received two intravenous injections of Alb-FAPtp-DOX or free DOX (4 mg/kg) on Days 20 and 25 (indicated by red arrows in panel D). Longitudinal PET/CT imaging was performed, and body weight and tumor volume were monitored daily until Day 40. (C) Representative PET/CT images of tumor-bearing mice acquired at 1, 2, and 3 h post-injection of ⁶⁸Ga-Alb-FAPtp-DOX. Prominent tracer accumulation was observed in the tumor, with additional signal detected in the heart (blood pool) and liver, and renal clearance evident in the bladder. Quantitative biodistribution is presented as SUVmean values for major organs, demonstrating stable tumor retention over the 3-h imaging window. Tumor-to-background ratios are shown for muscle (T/M), brain (T/B), heart (T/H), liver (T/L), and kidney (T/K), with high T/M and T/B ratios highlighting favorable imaging contrast. (D) Therapeutic efficacy assessment showing tumor growth curves for the control (Ctrl), free doxorubicin (DOX), and Alb-FAPtp-DOX (FAPtp-DOX) treatment groups (n = 3/group). Alb-FAPtp-DOX exhibited a trend toward greater tumor growth inhibition compared with free DOX; however, the difference did not reach statistical significance ($P > 0.05$), indicating limited therapeutic benefit of this formulation. Data are presented as mean ± s.d..

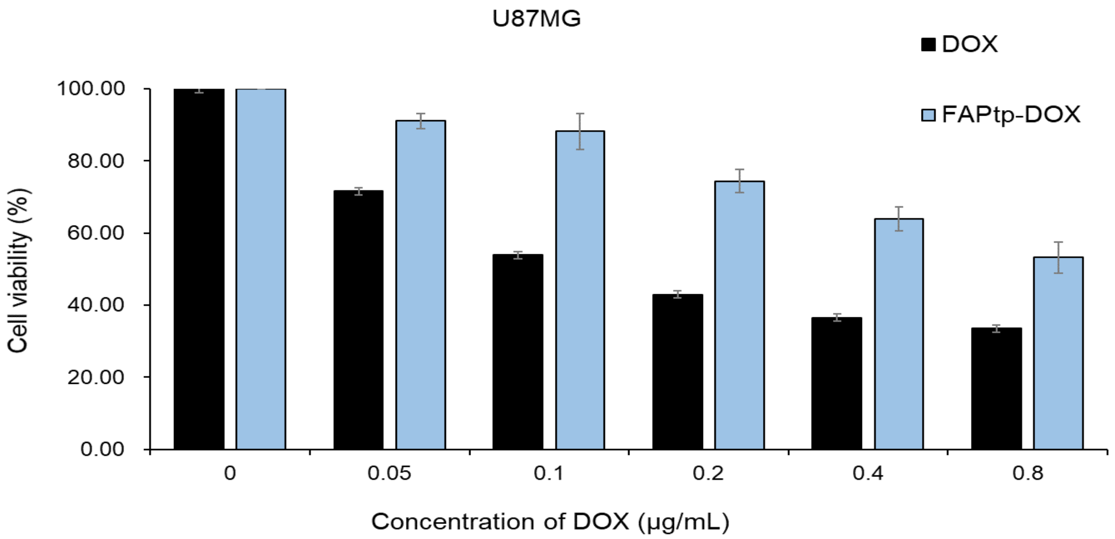

Figure S7. ***In vitro* cytotoxicity of free DOX and FAPtp–DOX prodrug.** Cells were treated with increasing concentrations of free DOX or FAPtp–DOX for 24 h, and cell viability was determined using a CCK-8 assay. Data are presented as mean ± s.d. (n = 3). The prodrug formulation exhibited reduced cytotoxicity compared to free DOX across the tested concentration range.

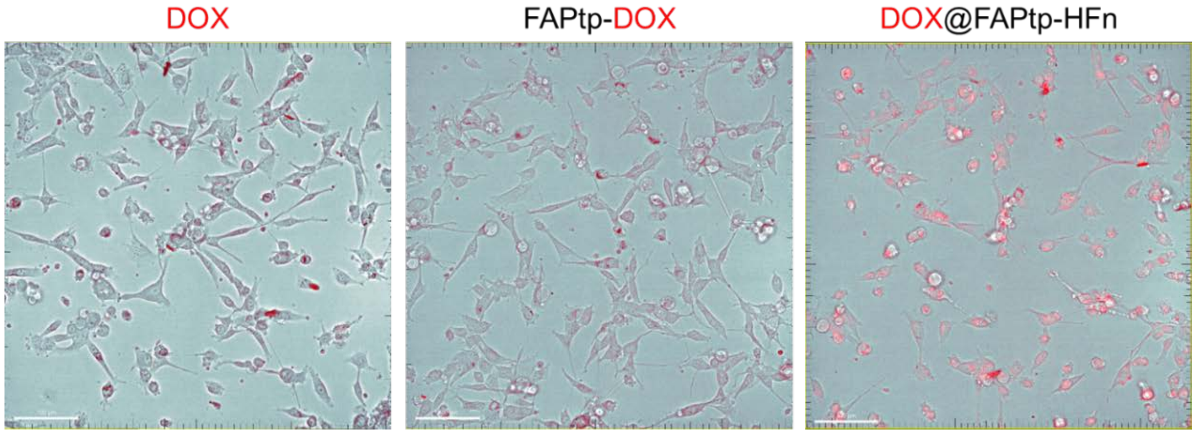

Figure S8. Comparative cellular uptake and subcellular localization.
Representative confocal microscopy images evaluating the intracellular distribution and availability of various doxorubicin (DOX) formulations in tumor cells after 30 minutes of incubation. The similar subcellular distribution indicating that inefficient linker cleavage design.

FAPtp-MMAE

Rationale for Transition to Ferritin-Based MMAE Delivery:

Although the FAPtp-DOX prodrug exhibited favorable tumor accumulation as confirmed by PET imaging, its therapeutic efficacy was limited, suggesting inefficient drug release at the tumor site. To enhance potency, a FAPtp-MMAE construct was subsequently developed; however, its highly hydrophobic nature led to poor biodistribution and diminished tumor uptake. These observations underscore a key limitation of small-molecule prodrug designs—namely, that the physicochemical properties of the payload can substantially compromise delivery efficiency and therapeutic outcome. Therefore, we shifted to a ferritin-based nanocarrier platform, which offers superior biocompatibility, enhanced payload versatility, and pH-responsive drug release properties suitable for efficient intracellular delivery of potent cytotoxins such as MMAE.

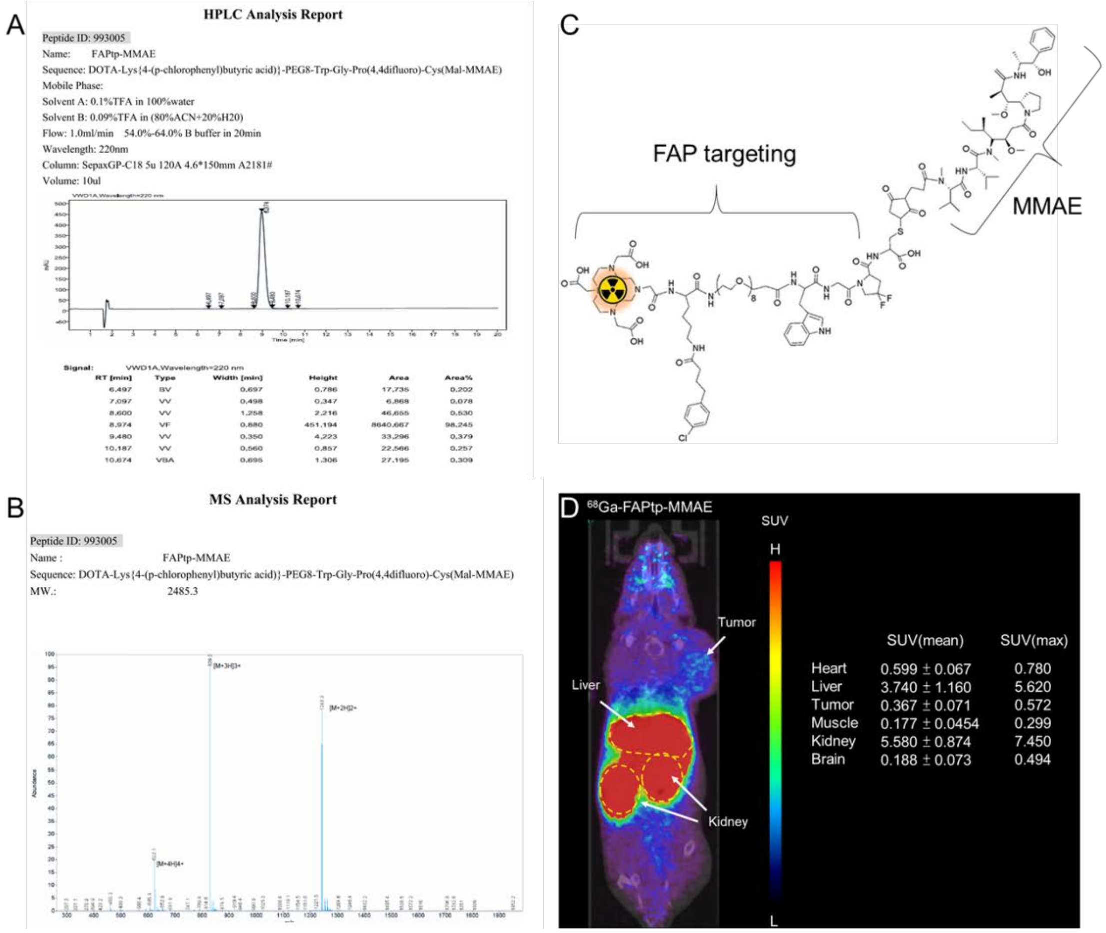

Figure S9. Characterization and *in vivo* imaging of ^{68}Ga-FAPtp-MMAE. (A) HPLC purity. (B) Mass spectrometry. (C) Chemical structure illustrating FAP targeting and payload. (D) PET/CT showing selective tumor and major organs accumulation with quantified SUVs.

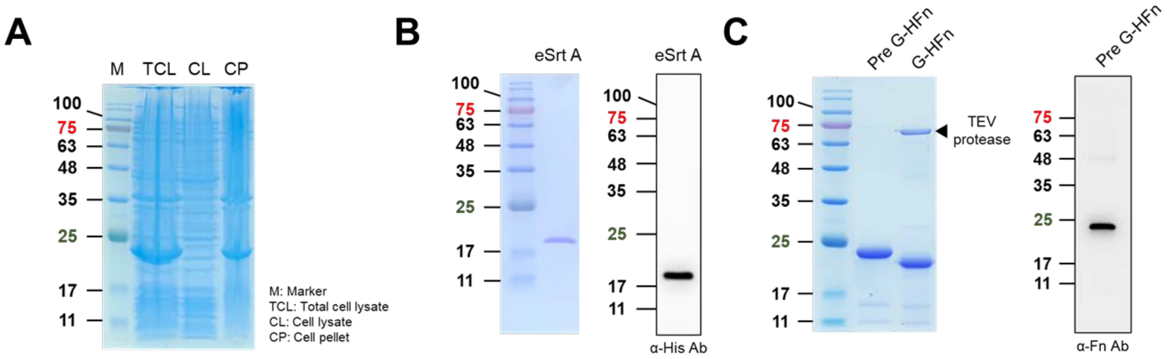

Figure S10. Protein expression and purification analyses related to HFn modification. (A) SDS-PAGE analysis of genetically modified W-HFn. (B) SDS-PAGE and Western blot analysis of purified eSortase A (eSrtA). (C) SDS-PAGE and Western blot analysis of Pre G-HFn and G-HFn.

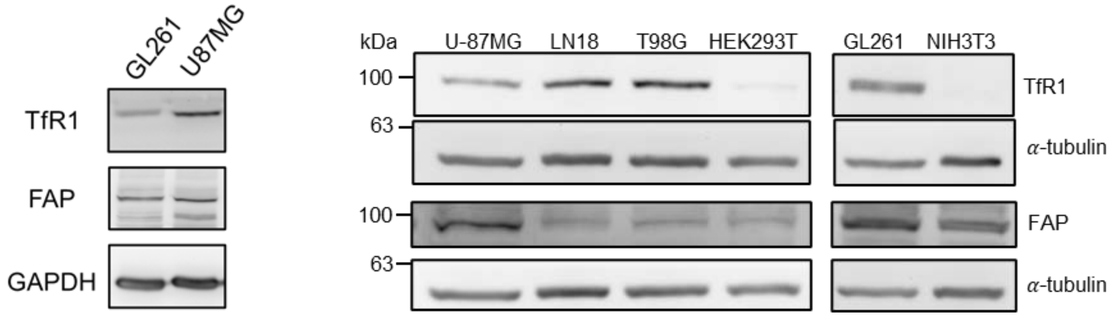

Figure S11. TfR1 and FAP Protein Expression. Western blot analysis of target expression across various cell lines. TfR1 and FAP protein levels were evaluated in human (U-87MG, LN18, T98G, HEK293T) and murine (GL261, NIH3T3) lines. High co-expression of both targets in glioma lines (U-87MG, GL261) validates the selection of these models for the dual-targeted strategy.

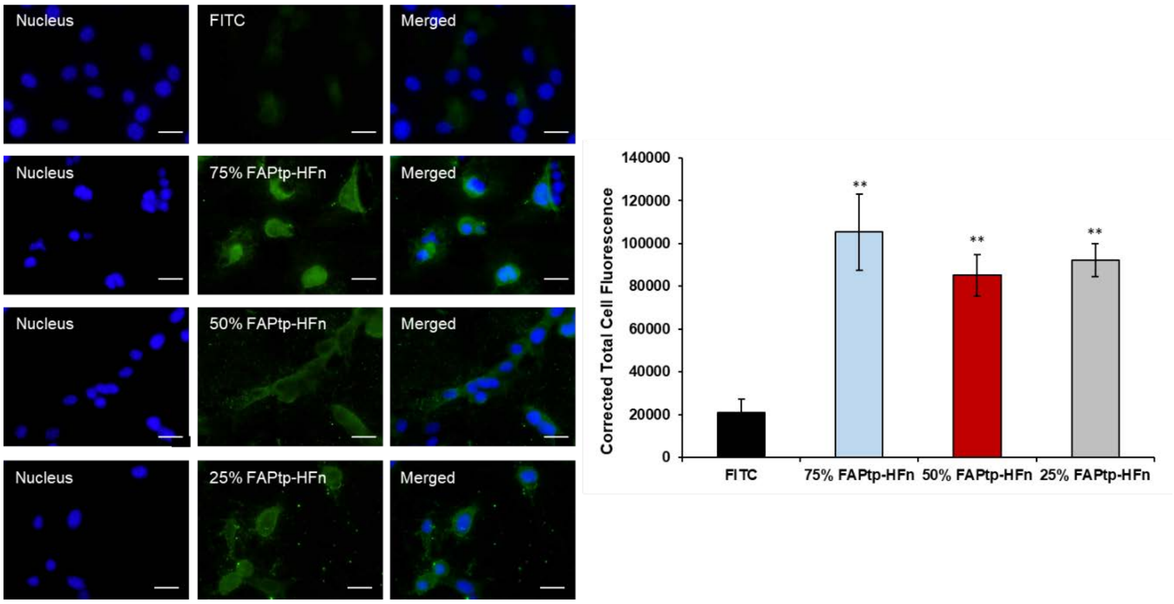

Figure S12. Cellular uptake analysis of FAPtp-HFn nanocarriers. (Left) Representative fluorescence images of cells incubated with free FITC (control) and different formulations of FAPtp-HFn (75%, 50%, and 25% modification density). Blue fluorescence indicates DAPI-stained nuclei; green fluorescence represents FITC-labeled nanocarriers. The rightmost column shows the merged images. Scale bars 20 µm. (Right) Quantitative analysis of cellular uptake based on Corrected Total Cell Fluorescence (CTCF). All FAPtp-HFn groups exhibited significantly higher internalization compared to the free FITC group (** $p < 0.01$). Data are presented as mean ± s.d..

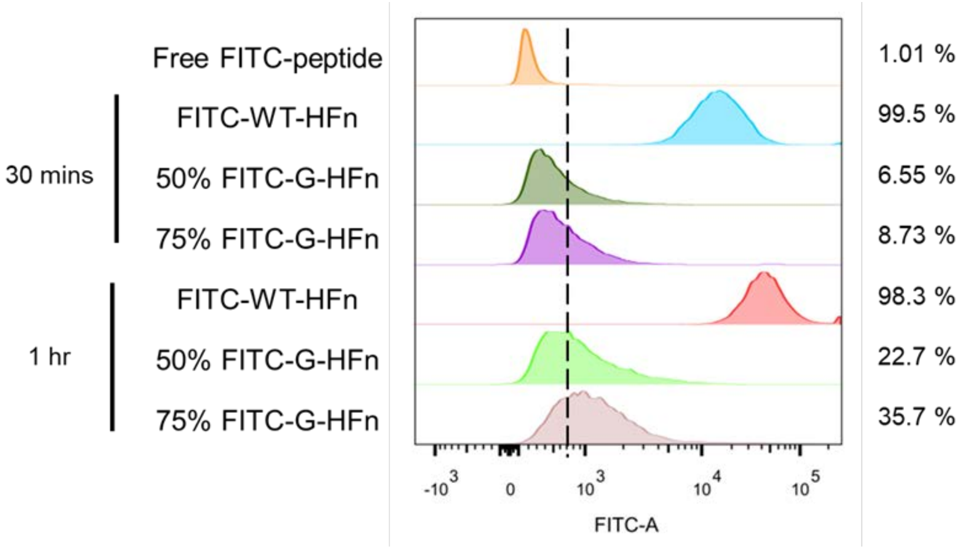

Figure S13. Evaluation of TfR1 binding affinity via flow cytometry. Surface modification of FITC-labeled non-targeting control peptide at the N-terminus of ferritin (HFn) results in a transient attenuation of TfR1-mediated cellular interaction followed by partial recovery.

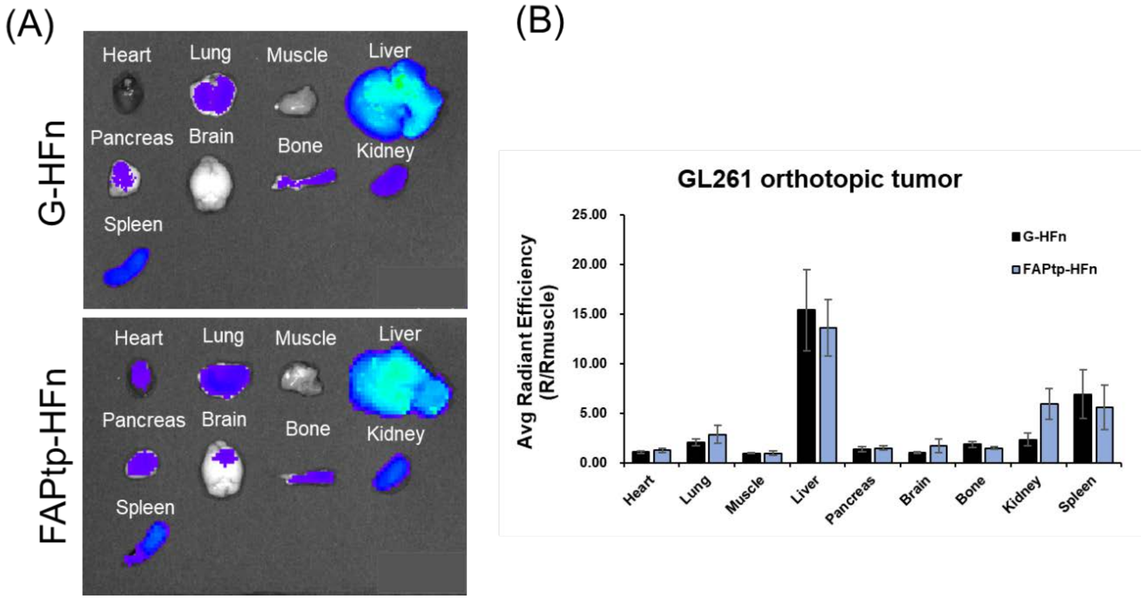

Figure S14. ***Ex vivo*** **biodistribution of ferritin nanocarriers in orthotopic GL261 tumor-bearing mice.** (A) Representative *ex vivo* fluorescence images of harvested organs (Heart, Lung, Muscle, Liver, Pancreas, Brain, Bone, Kidney, and Spleen) 24 hours post-injection of G-HFn or FAPtp-HFn. (B) Quantitative analysis of the average radiant efficiency (R/Rmuscle) in major organs. Data are presented as mean ± s.d. (n = 3). Consistent with subcutaneous models, high uptake was observed in RES-related organs (liver and spleen), while the FAPtp-HFn group exhibited superior accumulation in the brain tumor lesion site compared to the G-HFn group, supporting the efficacy of the dual-targeting strategy in the orthotopic setting.

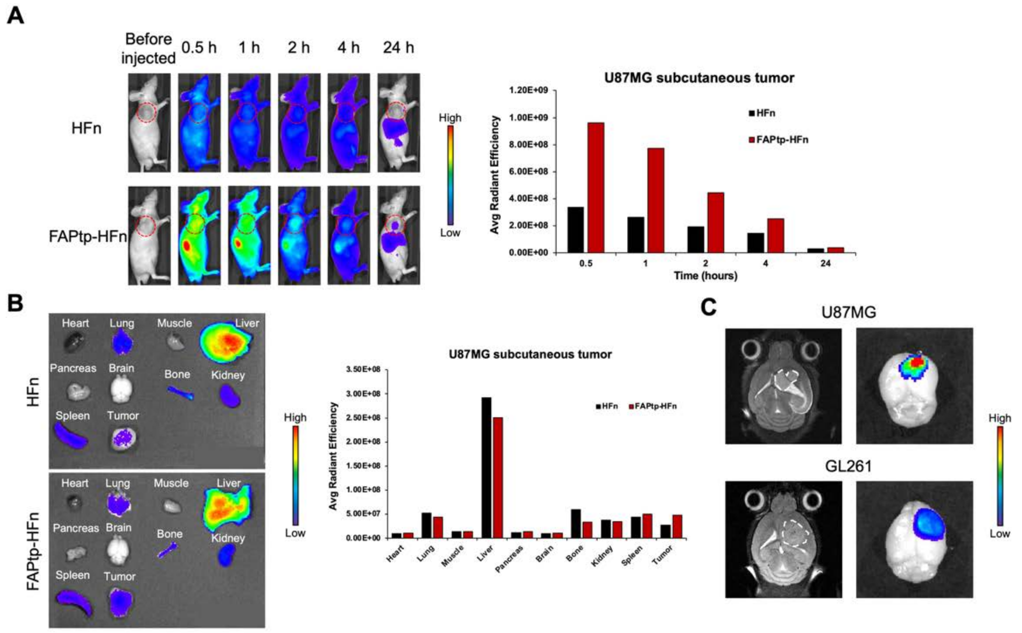

Figure S15. ***In vivo*** **tumor-targeting capability and biodistribution of FAPtp-HFn.** (A) In vivo tumor-targeting ability of FAPtp-HFn in a subcutaneous glioma mouse model. (B) Biodistribution of FAPtp-HFn in U-87MG tumor-bearing mice at 24 h post-intravenous injection. (C) Fluorescence signals in orthotopic tumors of U-87MG and GL261 tumor-bearing mice at 24 h post-injection.

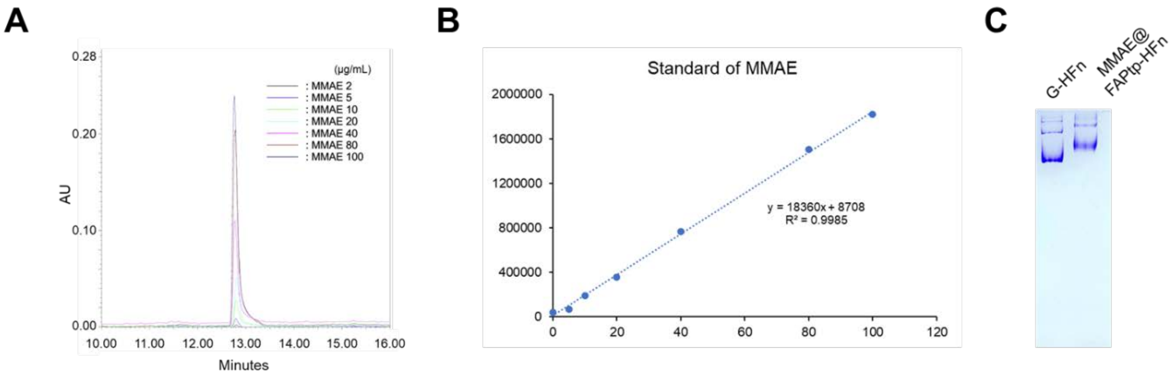

Figure S16. MMAE was quantified using high-performance liquid chromatography (HPLC). (A) Representative HPLC analysis of MMAE at different concentrations. (B) Standard calibration curve of MMAE at varying concentrations. (C) Native gel analysis of MMAE encapsulated within FAPtp-HFn.

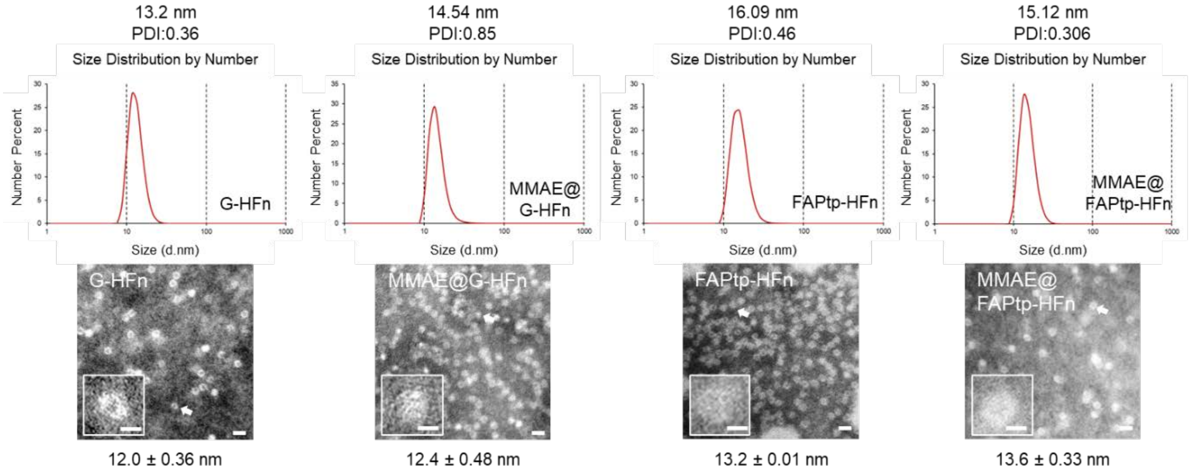

Figure S17: Physicochemical Characterization of Ferritin Nanocages using DLS and TEM

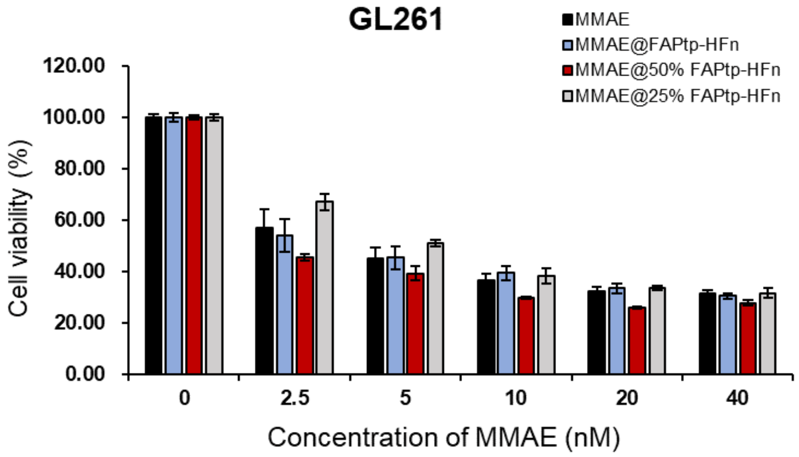

Figure S18. ***In vitro*** **cytotoxicity of MMAE formulations against GL261 cells.** Cell viability of GL261 cells incubated with free MMAE, MMAE@FAPtp-HFn, MMAE@50% FAPtp-HFn, and MMAE@25% FAPtp-HFn at various concentrations (0–40 nM) for 48 h. Cell viability was evaluated via the CCK-8 assay. Data are presented as mean ± s.d. (n = 3). Statistical comparisons were determined by one-way ANOVA with Tukey's HSD post-hoc test. No significant difference was observed between the treatment groups at the same equivalent MMAE concentrations ($P > 0.05$)

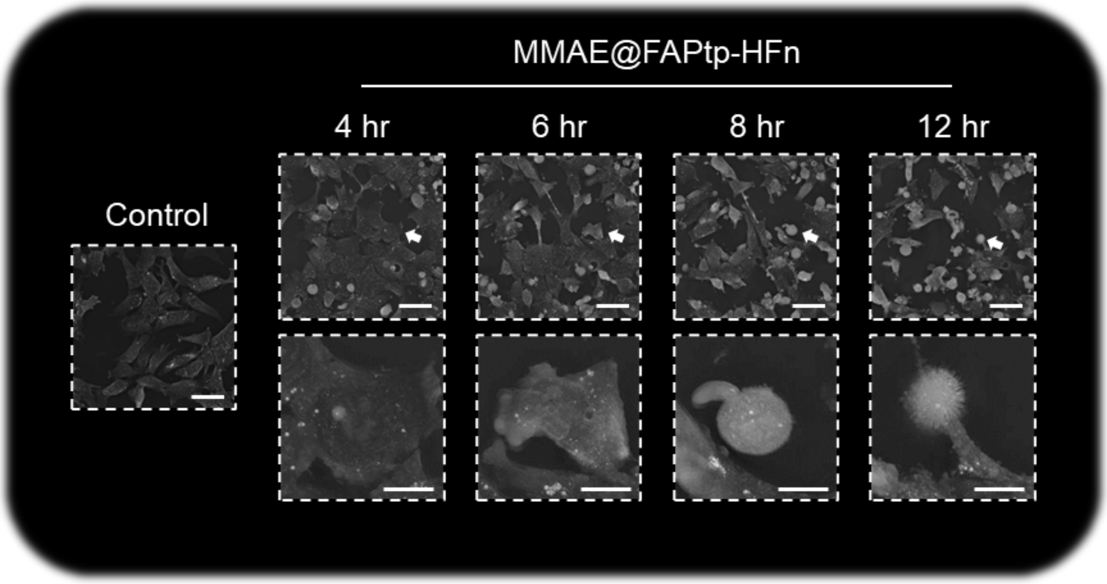

Figure S19. Nanolive imaging reveals time-dependent morphological changes and cell death induced by MMAE@FAPtp-HFn. Nano live cell images of GL261 cells, either untreated or treated with MMAE@FAPtp-HFn (40 n_M), showing progressive morphological alterations over time. Scale bars: 50 μm (original images); 10 μm (enlarged images).

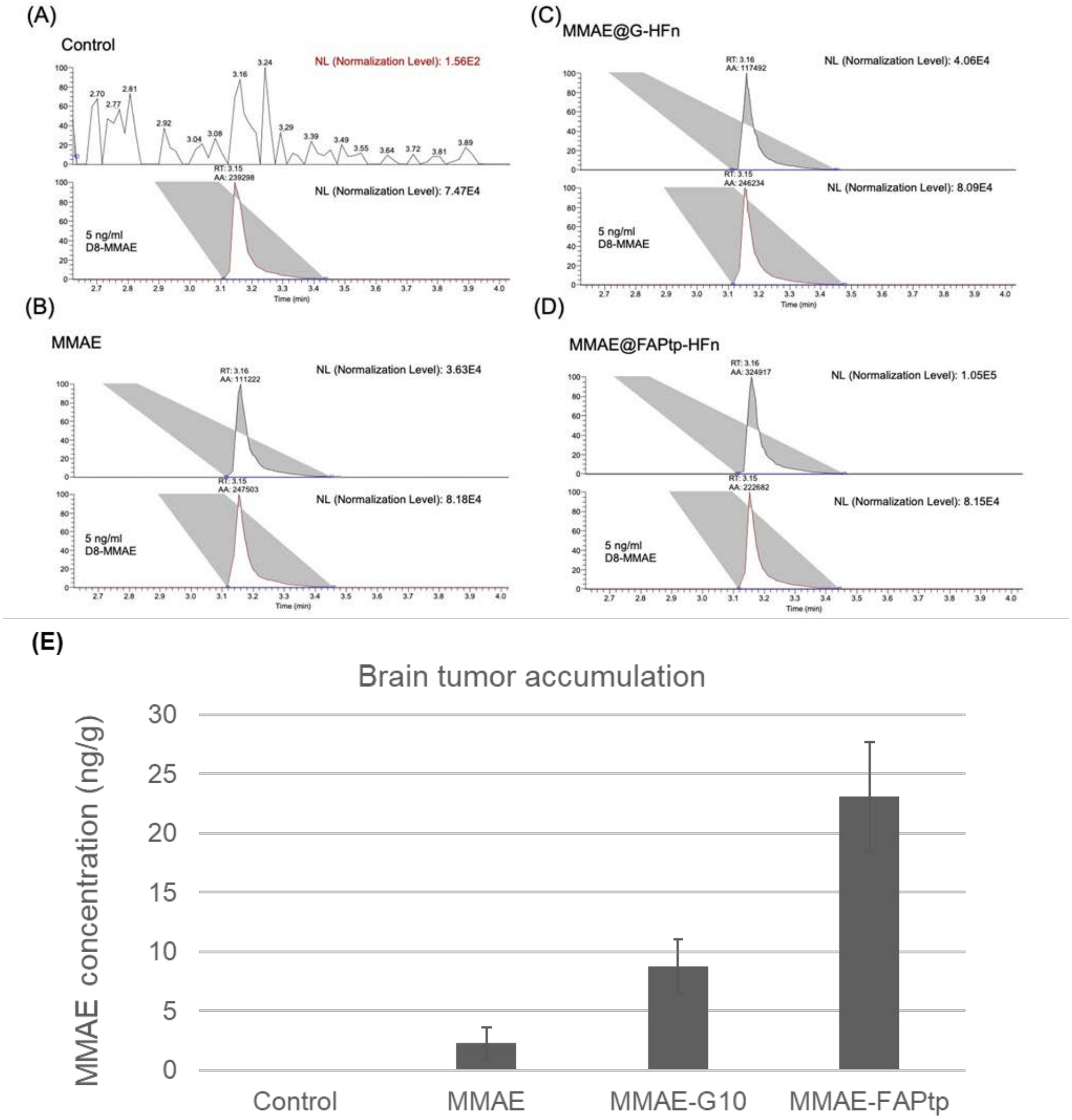

Figure S20. LC-MS/MS quantitative analysis of MMAE accumulation in brain tumor tissues. (A–D) LC-MS/MS extracted ion chromatograms (EICs) of MMAE and the internal standard d_8-MMAE, 5 ng/mL) from brain tumor lysates: (A) Control, (B) free MMAE, (C) MMAE@G-HFn, and (D) MMAE@FAPtp-HFn. RT indicates the retention time of the targeted analytes. (E) Quantification of MMAE concentration (ng/g) in the tumor tissue from a pilot study. The data illustrate the potential of FAP-targeting nanoparticles (MMAE@FAPtp-HFn) to deliver higher drug payloads to the tumor site compared to non-targeted controls.

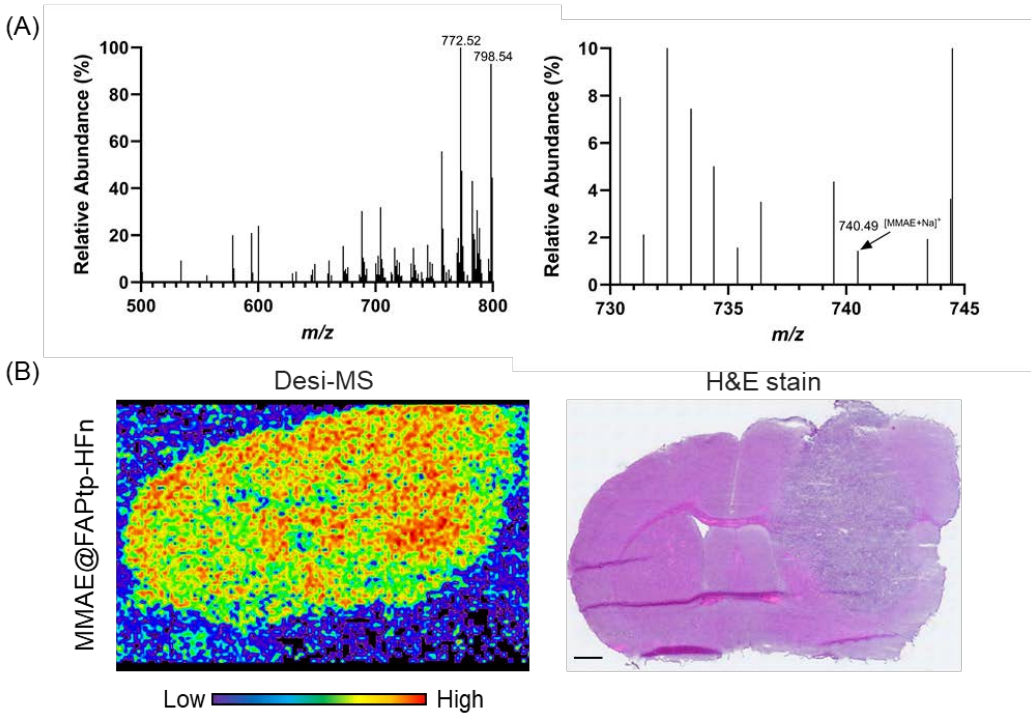

Figure S21. Characterization and spatial distribution of MMAE@FAPtp-HFn in brain tissues. (A) Representative DESI-MS spectra of MMAE@FAPtp-HFn. Left: Full-scan mass spectrum ranging from m/z 500 to 800. Right: Magnified mass spectrum (m/z 730–745) identifying the MMAE characteristic peak at m/z 740.49, corresponding to the $[MMAE+Na]^+$ adduct. All intensities are normalized to the base peak (100% relative abundance). (B) Spatial distribution analysis in GL261 tumor-bearing brain sections. Left: DESI-MS imaging illustrating the relative intensity and localization of MMAE. The heat map indicates intensity levels from low (blue) to high (red). Right: Corresponding H&E-stained histological section of the same brain tissue for anatomical correlation. Scale bar = 1 mm.

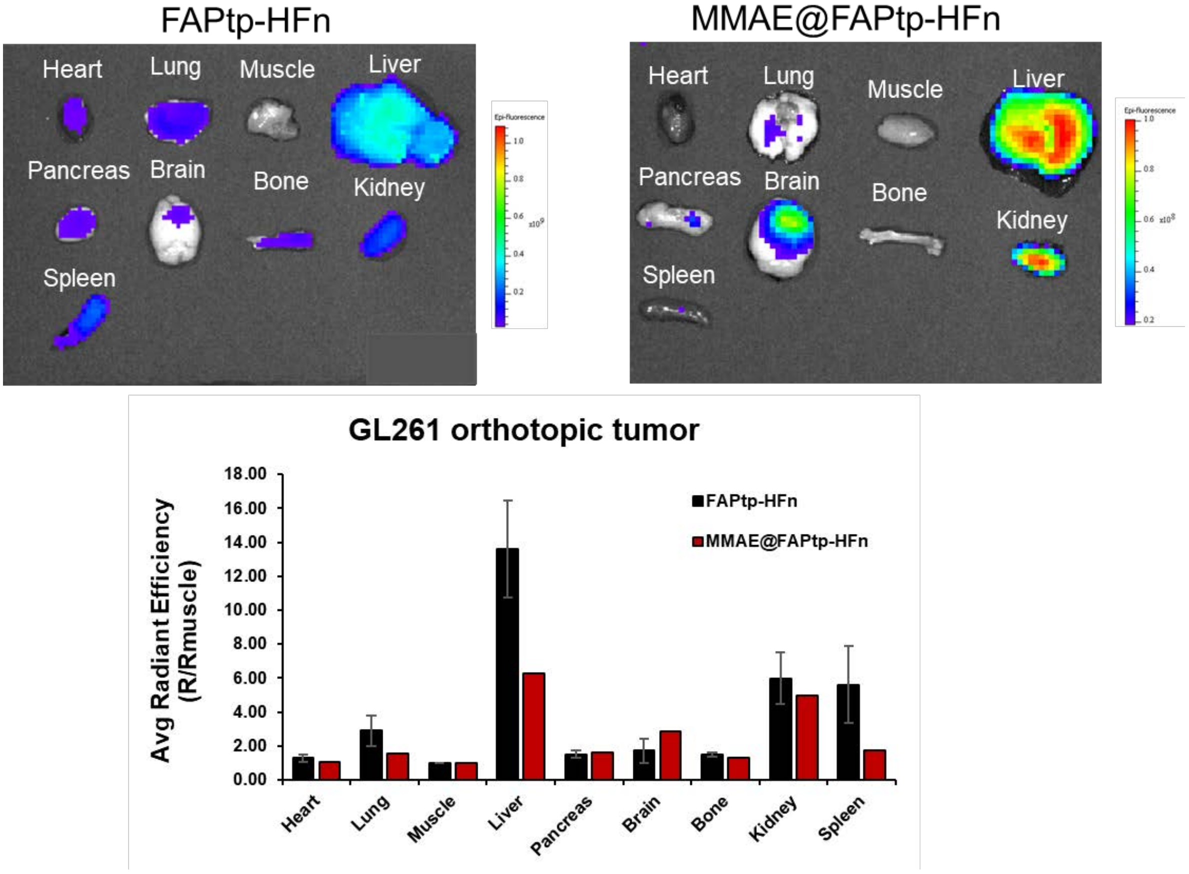

Figure S22. ***Ex vivo*** **biodistribution of FAPtp-HFn and MMAE@FAPtp-HFn in GL261 tumor-bearing mice.** (Top) Representative *ex vivo* fluorescence images of major organs harvested from mice treated with Cy7-labeled FAPtp-HFn or MMAE@FAPtp-HFn. Organs include heart, lung, muscle, liver, pancreas, brain, bone, kidney, and spleen. (Bottom) Quantitative analysis of organ fluorescence intensity. To normalize for variations in the injected dose, the average radiant efficiency of each organ was divided by the muscle signal of the same animal (R/Rmuscle). The results demonstrate that the encapsulation of MMAE maintains the intrinsic biodistribution profile of the HFn carrier, showing predominant accumulation in metabolic organs and successful localization in the brain.

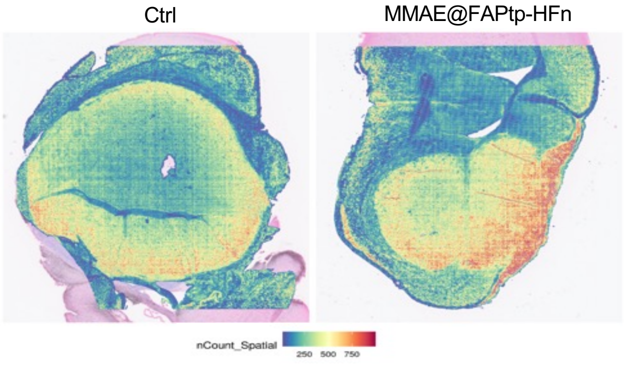

Figure S23. Spatial feature plot of total UMI counts (nCount) across brain sections from control and treatment groups. This plot displays the total number of transcripts (nCount) captured per spot, serving as a quality control metric for RNA capture efficiency and tissue integrity. Regions with higher nCount values indicate areas of increased RNA capture, often corresponding to dense cellular or transcriptionally active regions. In contrast, lower nCount values observed in the tumor core may reflect localized necrosis or RNA degradation.

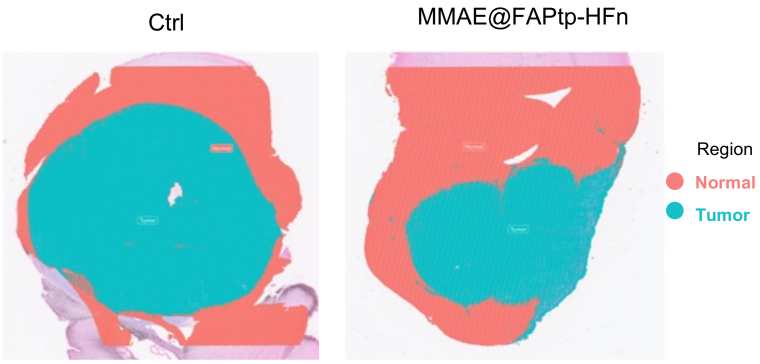

Figure S24. Spatial mapping of clusters from integrated UMAP analysis onto the brain tissue section. The spatial plot shows that the identified clusters correspond well with anatomical regions, clearly distinguishing tumor-infiltrated areas from normal brain tissue based on their spatial location.

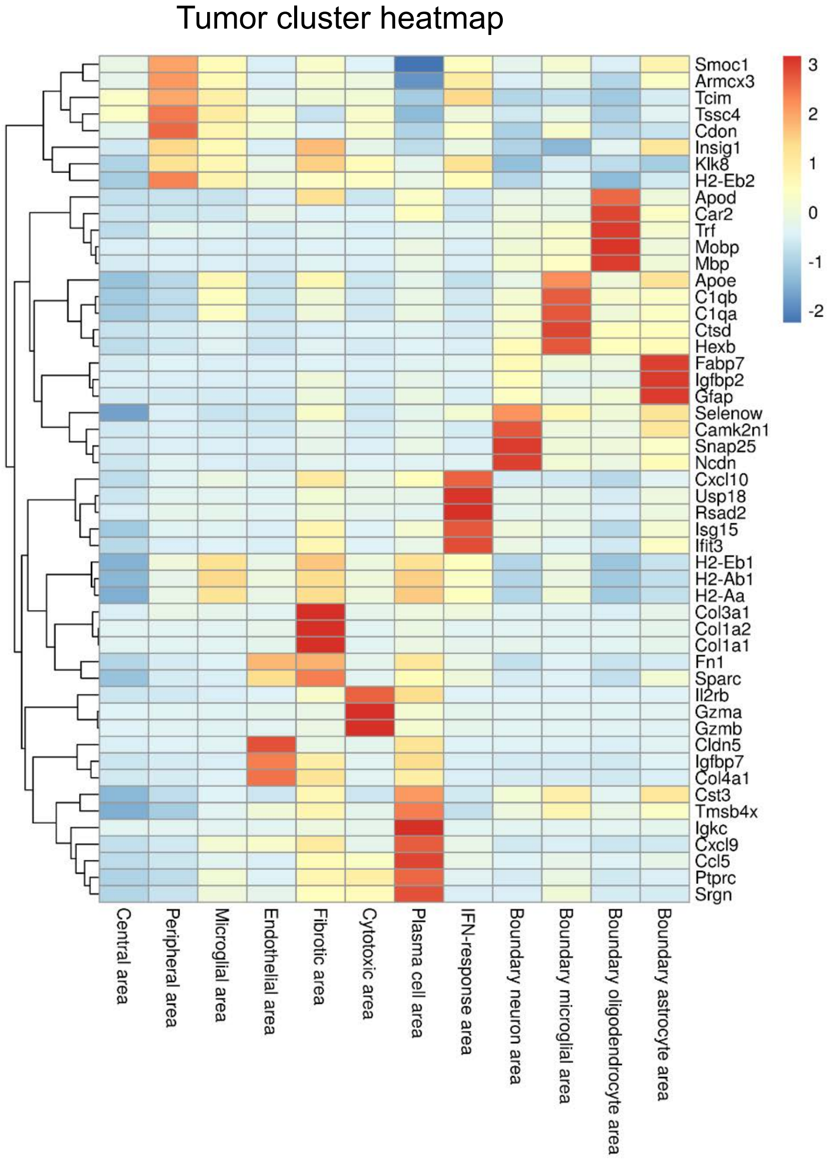

Figure S25. The heatmap of DEGs of these 12 clusters in the tumor region. From the global clustering of the spatial transcriptomics data, 12 distinct clusters were identified in the tumor area. These clusters represent transcriptionally and anatomically diverse regions of the tumor tissue.

(A) (B)

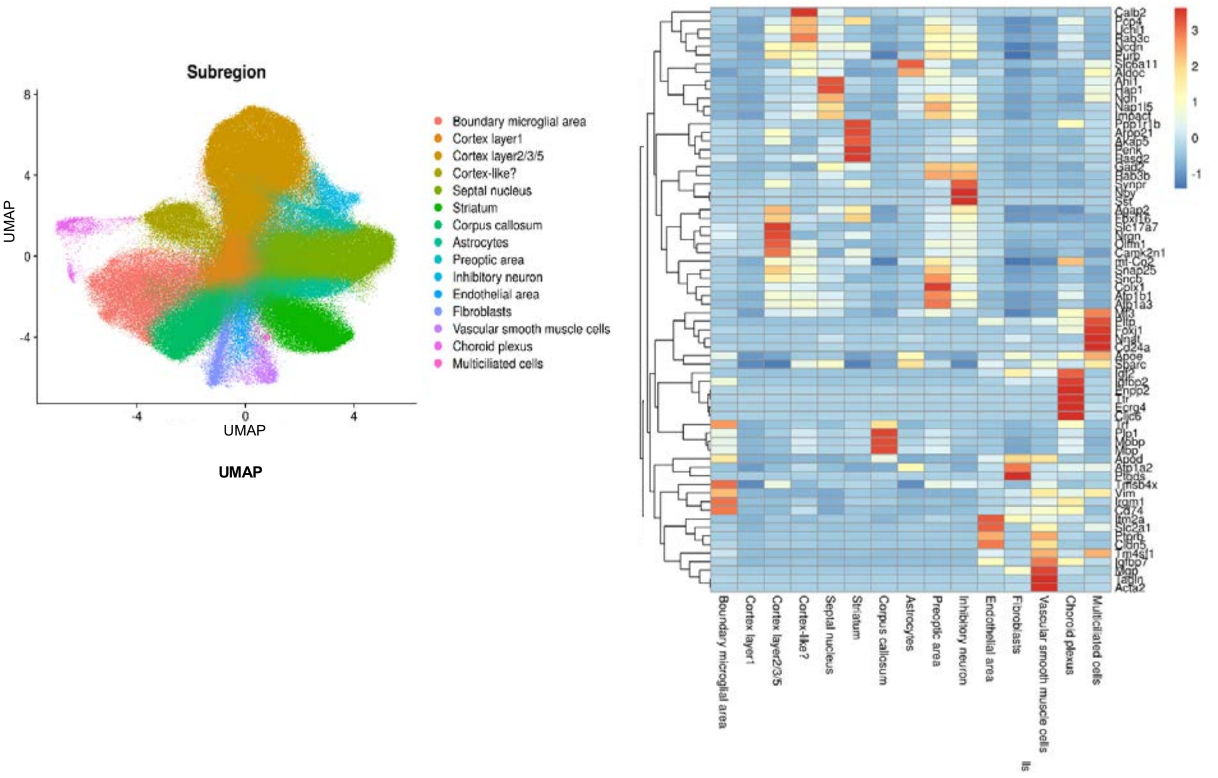

Figure S26. Identification of 15 clusters within the normal brain region. From the global clustering of the spatial transcriptomics data, 15 distinct clusters were identified in the normal brain area. These clusters represent transcriptionally and anatomically diverse regions of healthy brain tissue. These normal-region clusters serve as a reference for comparison with tumor-associated transcriptional changes. (A) UMAP plots for these 15 clusters. (B) The heatmap of DEGs of these 15 clusters.

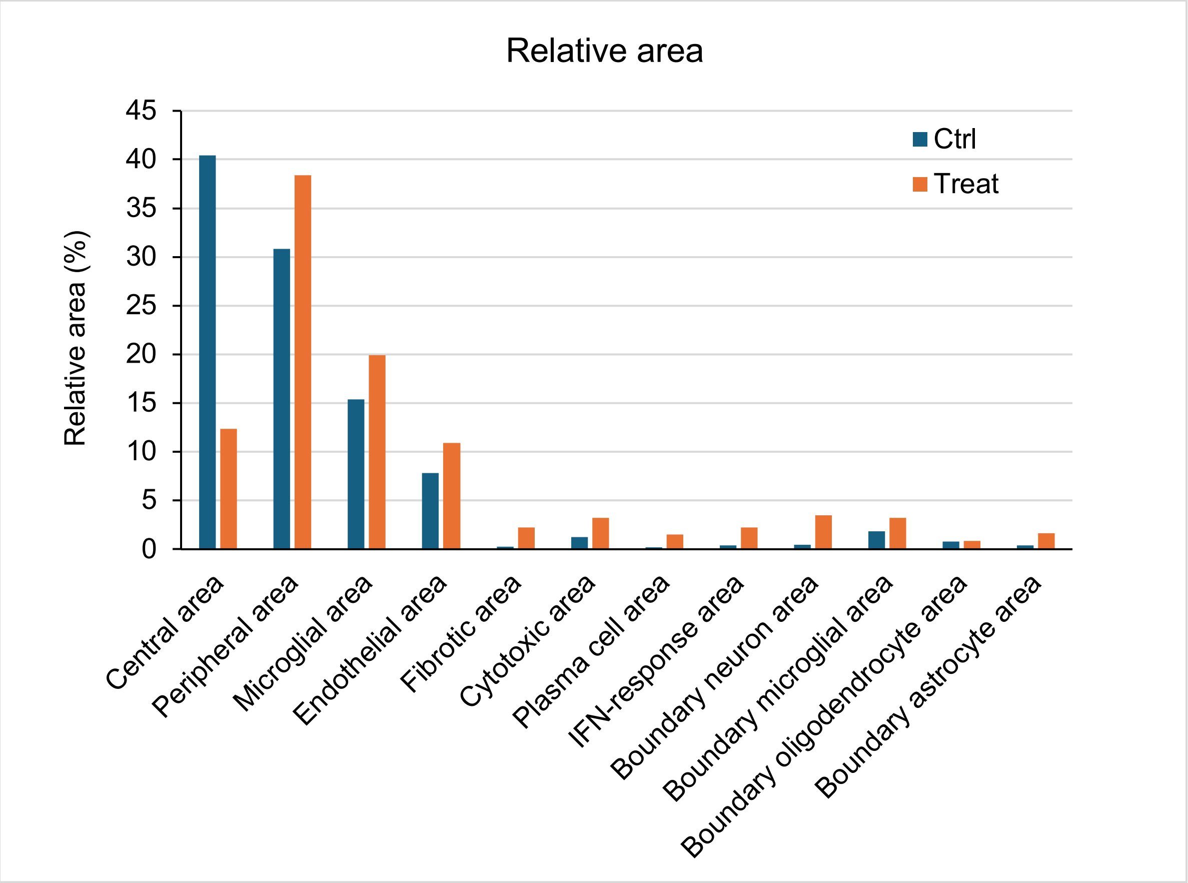

Figure S27. Spatial composition analysis of tumor regions in control and MMAE@FAPtp-HFn-treated groups. The bar graph displays the relative proportion of four dominant spatial clusters—central tumor area, peripheral tumor area, microglial cells, and endothelial cells—within tumor tissues from control and MMAE@FAPtp-HFn-treated samples. Treatment significantly reduced the central tumor area while increasing the representation of peripheral, microglial, and endothelial clusters, indicating a shift in tumor composition and microenvironmental architecture.

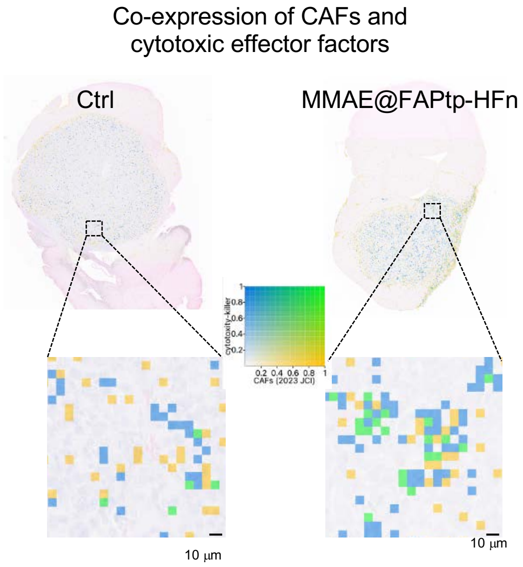

Figure S28. Spatial composition analysis of tumor regions in control and MMAE@FAPtp-HFn-treated groups. We identified 22,727 CAF-positive spots, 11,182 cytotoxic effector–enriched spots, and 1,549 co-expressed spots in the control group. In the MMAE@FAPtp-HFn-treated group, these numbers were 27,347 CAF-positive spots, 17,819 cytotoxic effector–enriched spots, and 5,412 co-expressed spots. Quantitative assessment revealed that 13.9% of cytotoxic effector-enriched spots co-expressed CAF markers in control tumors, which significantly increased to 30.3% in MMAE@FAPtp-HFn-treated tumors (Figure 7G).

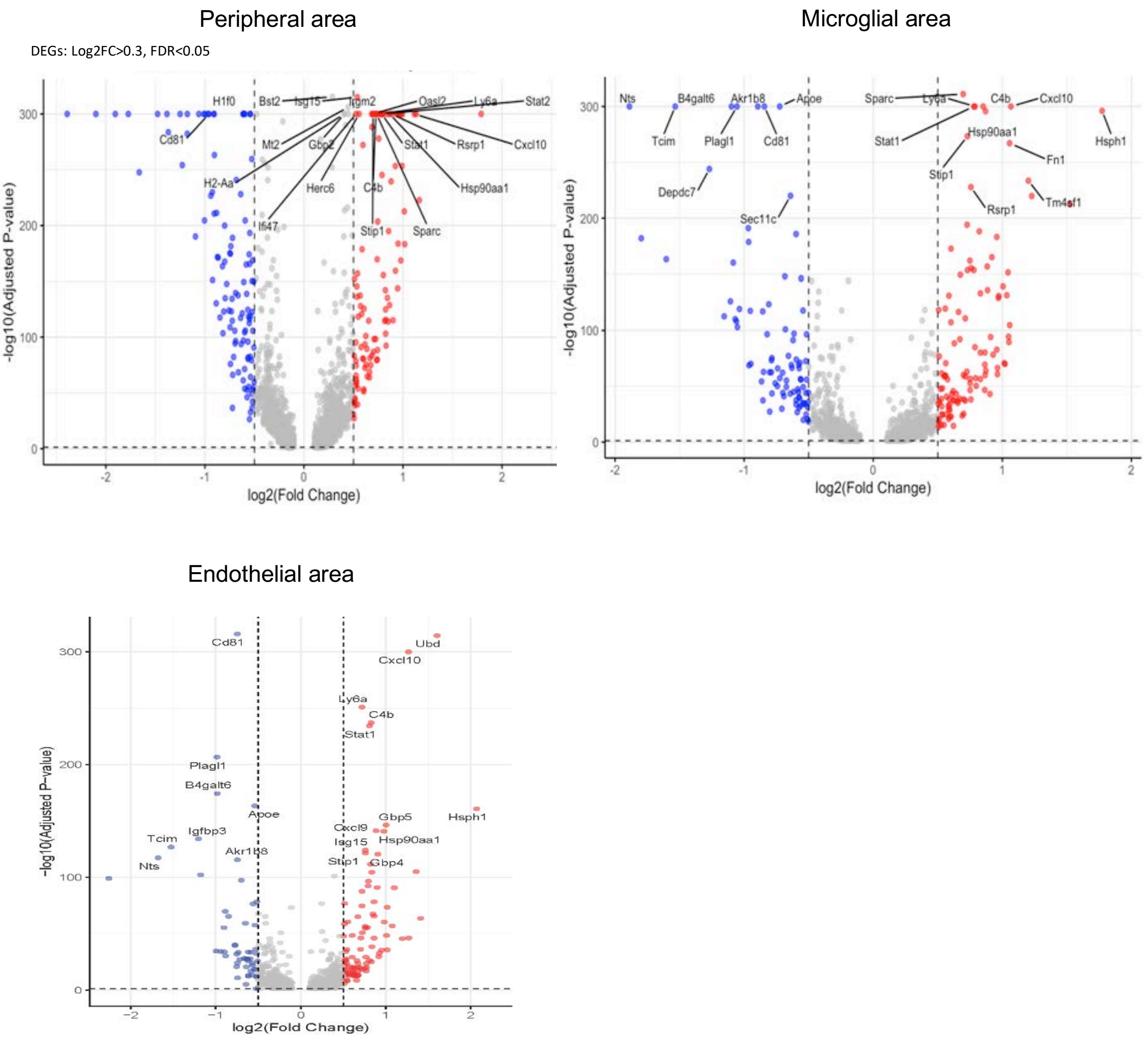

Figure S29. Volcano plot of the peripheral, microglial-rich and endothelial area of tumor regions in control and MMAE@FAPtp-HFn-treated groups. Significant treatment-upregulated DEGs are shown in red; significant treatment-downregulated DEGs are shown in blue. The top 15 DEGs are labeled by gene symbol (FDR < 0.05, $log_2FC > 0.3$).

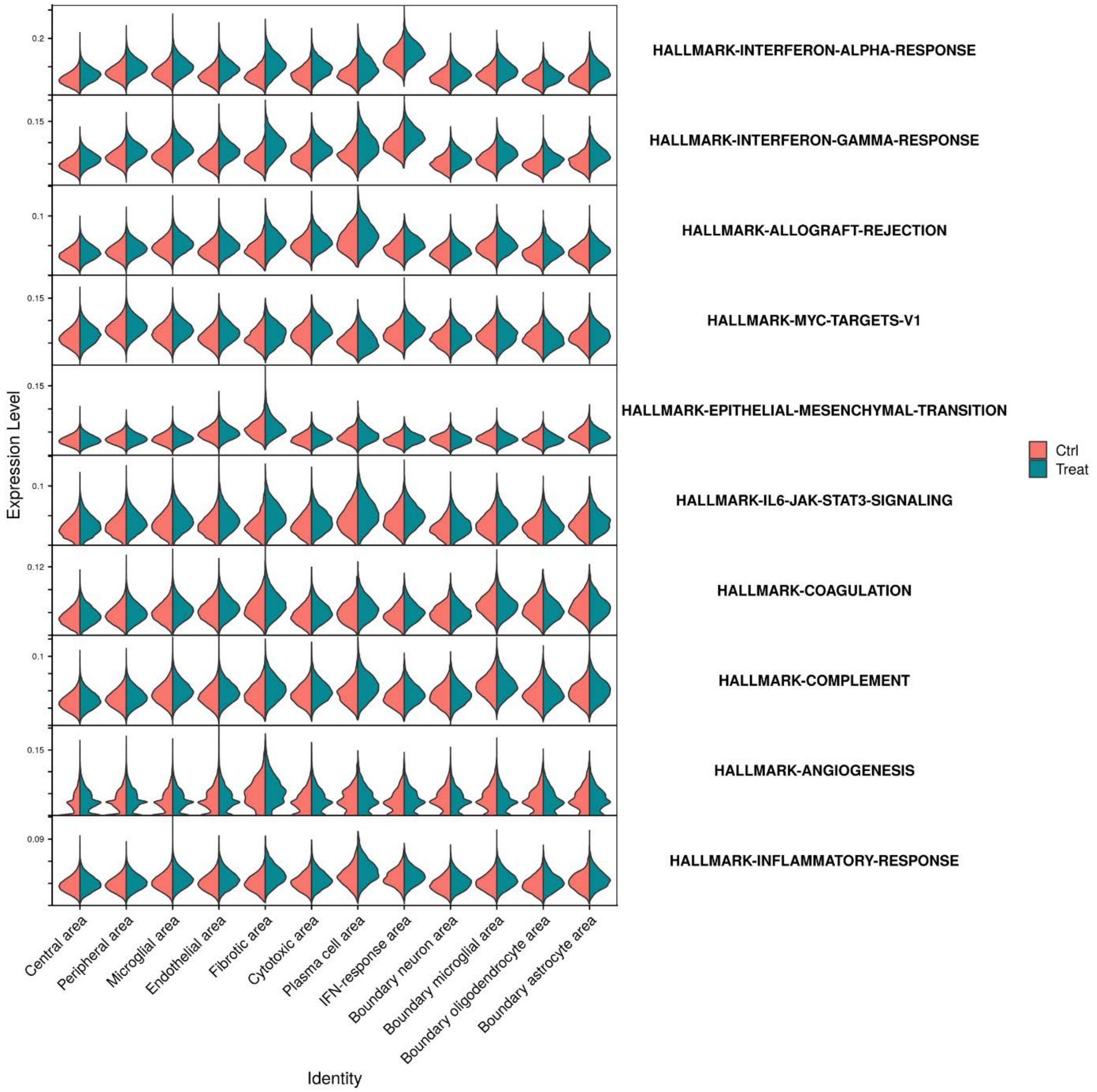

Figure S30. AUCell-based enrichment analysis of IFN-α and IFN-γ response pathways across tumor clusters.

Violin plots show AUCell scores for Hallmark IFN-α response and IFN-γ response gene sets across 12 transcriptionally defined clusters identified in the spatial transcriptomic analysis. Each violin represents the distribution of pathway activity scores within a cluster, including samples from both control and treatment groups. Notably, treatment-associated clusters exhibit higher enrichment scores for these immune-related pathways, indicating an enhanced pro-inflammatory and anti-tumor immune response following CAF-targeted nanoparticle therapy.

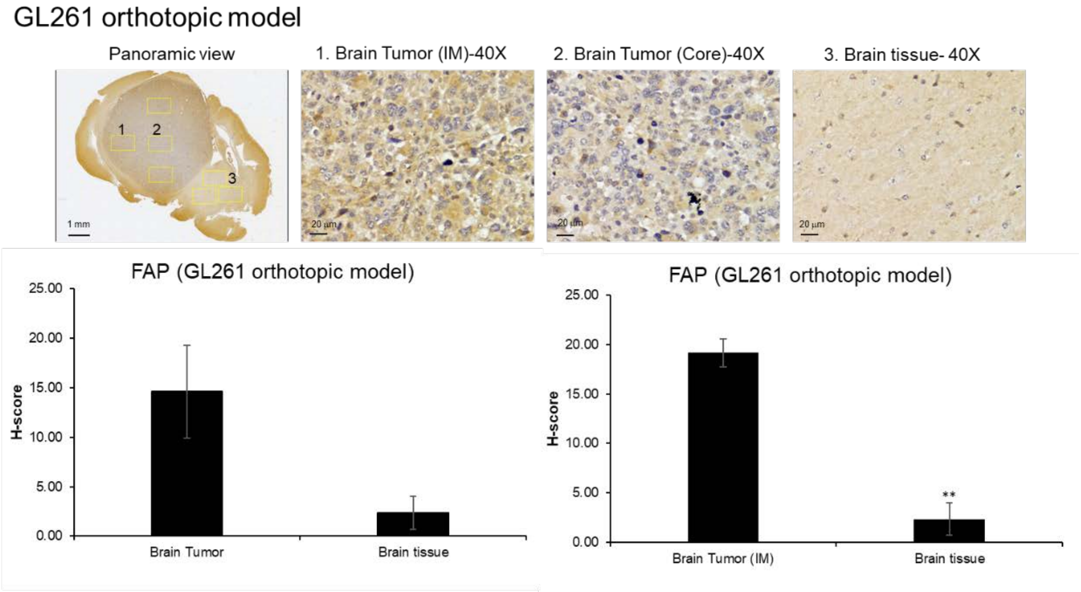

Figure S31. Spatial distribution and quantification of FAP in the GL261 orthotopic mouse model. (Top) Representative IHC images showing FAP expression in the GL261 orthotopic glioma model. The panoramic view (scale bar = 1 mm) illustrates the tumor's location within the brain. High-magnification images reveal distinct FAP staining patterns in the invasive margin (IM), tumor core, and surrounding normal brain tissue (scale bar = 20 µm). (Bottom) H-score quantification comparing FAP expression between tumor regions and normal brain tissue. Significant upregulation of FAP was observed in the tumor area, particularly at the invasive margin (IM), compared to healthy brain tissue (** $p < 0.01$). Data are expressed as mean ± s.d..

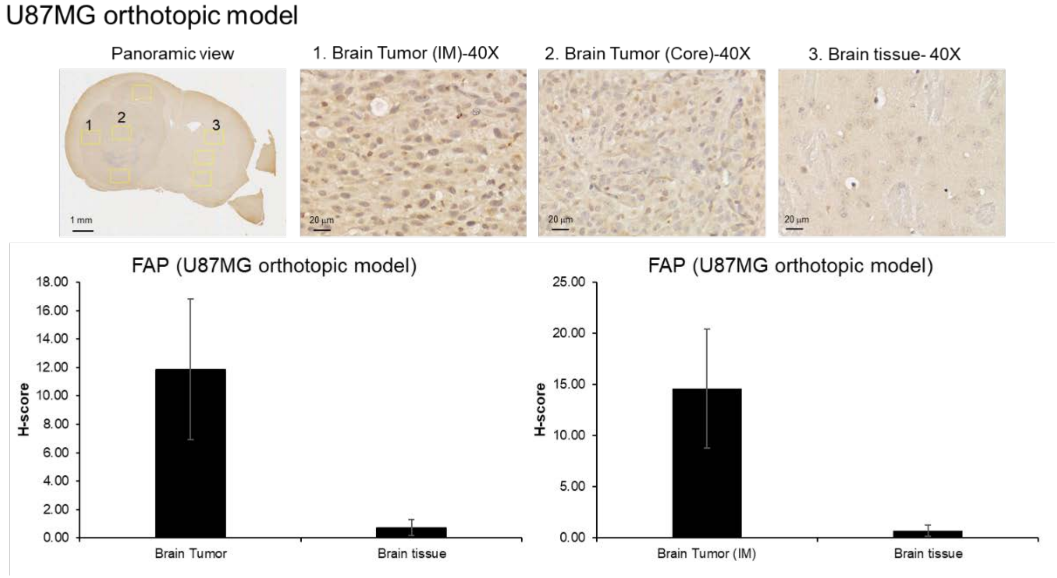

Figure S32. **Spatial distribution and quantification of FAP in the U-87MG orthotopic mouse model.** (Top) Representative IHC images of FAP in the U-87MG orthotopic model. The panoramic view (scale bar = 1 mm) illustrates the tumor's location within the brain. Magnified images provide cellular details of FAP staining in the tumor invasive margin (IM), tumor core, and surrounding normal brain tissue (scale bars = 20 µm). (Bottom) Quantitative analysis of FAP expression (H-score) in different regions. Although higher mean FAP levels were observed in the tumor regions (including the IM) compared to normal brain tissue. No significant differences detected. Data shown as mean ± s.d..

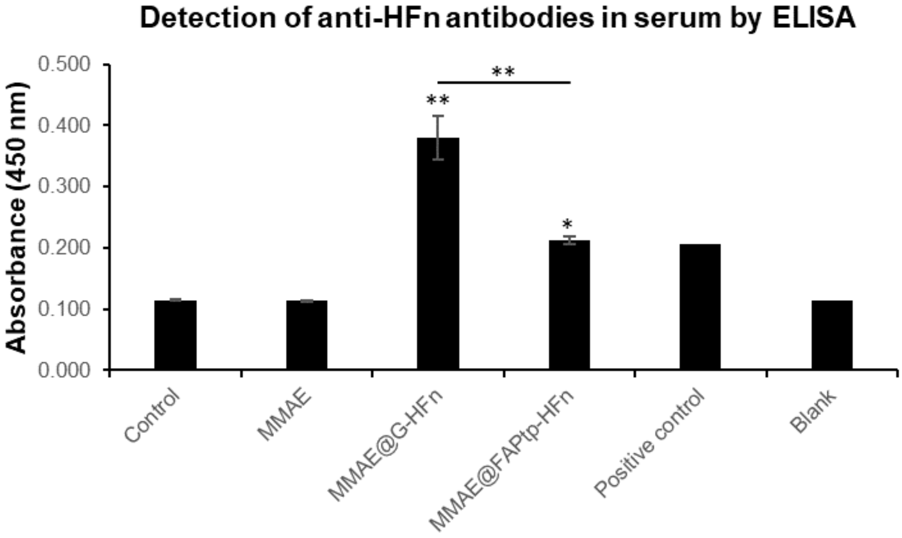

Figure S33. ELISA analysis of serum anti-ferritin IgG levels at different dilutions.
Mice were treated with various MMAE-loaded ferritin formulations. Serum immunogenicity was evaluated against a recombinant ferritin antigen at a 1:2000 dilution. A commercial anti-ferritin antibody (sc-376594) served as the positive control. Data represent mean ± s.d. (n = 3). Statistical analysis was performed using One-way ANOVA followed by Tukey's HSD post-hoc test. *$p < 0.05$, **$p < 0.01$.

Table S1. Characteristics of Radiolabeled Ligands

Tracers	Radiochemical purity	Specific activity (MBq/nmol)	Log P (n=3)
^{68}Ga-FAPI-04	> 95%	10.76-4.31	-2.92 ± 0.05
^{68}Ga-FAPI-46	> 97%	10.93-4.37	-2.74 ± 0.08
^{68}Ga-Alb-FAPtp-02	> 97%	18.43-7.37	-2.88 ± 0.12

Quality Control Result
Gallium ^{68}Ga-FAP Injection

Test Item	Specification	Result
Appearance	Clear, particulate-free solution	Pass
Ethanol Content	≦ 5%	Pass
pH Value	4.0 – 8.0	6.3
Radiochemical purity	≧ 90%	100%
Chemical Impurity	≦ 3.06 µg/mL	Pass
Chemical identity (API)	RRT = 8.9 ± 0.13	RRT= 8.97
Radiochemical impurity (^{68}Ga(III) ion)	≦ 2%	0%
Radiochemical impurity (^{68}Ga in colloidal form)	≦ 3%	0%
Radionuclidic identity (^{68}Ga)	62 min ≦ $T_{1/2}$ ≦ 74 min	68.78 min
Radioactive Concentration	≧ 0.33 mCi/mL	10 mCi/mL
Radionuclidic Purity	≧99.9% at 0.511 MeV. 1.077MeV, 1.022MeV. 1.883 MeV and Compton scatter	100%
Radionuclidic impurity (Long-lived isotopes)[a]	≦ 0.001%	0%
Endotoxin	≦ 11.6 EU/mL	< 8 EU/mL
Sterility	Pass	Pass

[a] The preparation is retained for at least 48 h to examine long-lived radionuclidic impurities.

Table S2. Quality control specifications for the ^{68}Ga-labeled tracer. The final product must comply with these release criteria, established in accordance with the European Pharmacopoeia 8, prior to use in preclinical or clinical PET studies.

Table S3. Storage stability and physicochemical properties of various ferritin nanocarriers. The table summarizes the polydispersity index (PDI), hydrodynamic diameter (DLS), and zeta potential of G-HFn, MMAE@G-HFn, FAPtp-HFn, and MMAE@FAPtp-HFn. Stability was assessed at -20 °C and 4 °C for up to 3 weeks. Zeta potential values are presented as mean ± s.d. (n = 3). MMAE: monomethyl auristatin E; HFn: human ferritin heavy chain; DLS: dynamic light scattering; PDI: polydispersity index.

Temperature	Time	Nanocarrier	PDI	DLS (d.nm)	Zeta potential (mV)
**-20 °C**	Initial	G-HFn	0.36	13.2	-15.13 ± 0.58
	2 weeks	MMAE@G-HFn	0.85	14.54	-13.33 ± 0.27
	3 weeks		0.52	30.37 / 54.61	-12.13 ± 0.28
	Initial	FAPtp-HFn	0.46	16.09	-13.03 ± 0.48
	2 weeks	MMAE@FAPtp-HFn	0.30	15.12	-10.99 ± 0.62
	3 weeks		0.28	12.76	-10.63 ± 0.43
**4 °C**	1 week	MMAE@G-HFn	0.43	58.9	-12.77 ± 0.09
	1 week	MMAE@FAPtp-HFn	0.25	13.6	-11.30 ± 0.26

Table S4. Spot counts and co-expression analysis of CAF markers and cytotoxic effector genes. This table summarizes the absolute counts and proportions of spots expressing cytotoxic effector genes and CAF markers in the orthotopic GL261 brain tumor model analyzed by Visium HD spatial transcriptomics. Cytotoxic effector-positive spots are defined as those with expression greater than zero of any cytotoxic effector gene (*Gzmb, Gzma, Gzmk, Prf1, Nkg7, Ifng*). CAF marker-positive spots are defined as those with expression greater than zero of any CAF-associated gene (*Acta2, Dcn, Col1a1, Col1a2*). Co-expression spots refer to spots positive for both cytotoxic effector genes and CAF markers. Statistical analysis using the Chi-square test showed a highly significant difference in the proportion of co-expression spots between the control and treated groups ($p < 0.0001$).

Group	Co-expression spots (CAF+ & cytotoxic+)	Only cytotoxic+ spots (CAF-)	Total cytotoxic+ spots	Proportion of co-expression (%)
Control	1549	9633	11182	13.85
MMAE@FAPtp-HFn	5412	12407	17819	30.37

Table S5: Spatial Transcriptomics Sequencing and Mapping Metrics

Summary of sequencing and mapping statistics for control and MMAE@FAPtp-HFn-treated glioblastoma mouse brain samples processed using the 10x Genomics Visium HD platform. Metrics include the number of spatial bins located under tissue, mean reads and UMIs per bin, total genes detected, and mapping percentages to the probe set. High mapping rates and gene detection across both samples confirm the quality and reliability of the spatial transcriptomic data for downstream analyses.

Sample	Bins Under Tissue	Mean Reads per Bin	Mean UMIs per Bin	Total Genes Detected	Reads Mapped to Probe Set (%)	Reads Mapped Confidently to Probe Set (%)	Reads Mapped Confidently to Filtered Probe Set (%)	Half-Mapped Reads (%)	Split-Mapped Reads (%)
Control	546897	730.6	279.4	19033	98.4	98.1	97.8	0.2	0.1
Treated	456346	1014.6	319.8	19036	98.2	98	97.7	0.2	0

Table S6. Serum biochemical parameters following treatment

Values are presented as mean ± s.d. (n = 3).

Abbreviations: ALT, alanine aminotransferase; AST, aspartate aminotransferase; BUN, blood urea nitrogen. No statistically significant abnormalities in liver or renal function markers were observed among treatment groups ($P > 0.05$), indicating no overt systemic toxicity under the dosing conditions tested.

Treatment Group	ALT (GPT) (U/L)	AST (GOT) (U/L)	BUN (mg/dL)	Creatinine (mg/dL)
Control	11.0 ± 8.5	56.5 ± 36.1	25.0 ± 4.2	0.25 ± 0.07
MMAE@G10-HFn	4.5 ± 0.7	67.5 ± 51.6	22.5 ± 0.7	0.25 ± 0.07
MMAE@FAPtp-HFn	7.0 ± 0.0	55.0 ± 45.3	24.0 ± 5.7	0.25 ± 0.07

Table S7. Serum levels of pro-inflammatory cytokines following treatment

Serum concentrations of interleukin-6 (IL-6) and tumor necrosis factor-α (TNF-α) in mice treated with different MMAE formulations. Values are presented as mean ± s.d. (n = 3).

Abbreviations: IL-6, interleukin-6; TNF-α, tumor necrosis factor-α. No significant elevation of systemic pro-inflammatory cytokines was observed in the MMAE@FAPtp-HFn–treated group compared with controls, suggesting a low risk of acute inflammatory responses under the conditions tested.

Treatment Group	IL-6 (pg/mL)	TNF-α (pg/mL)
Control	9.61 ± 4.38	0.69 ± 0.78
MMAE@G10-HFn	42.18 ± 32.04	0.06 ± 0.10
MMAE@FAPtp-HFn	0.55 ± 0.49	0.36 ± 0.19
